# Hopf bifurcation without parameters in deterministic and stochastic modeling of cancer virotherapy, part II

**DOI:** 10.1016/j.jmaa.2022.126444

**Published:** 2022-06-23

**Authors:** Tuan Anh Phan, Jianjun Paul Tian

**Affiliations:** a Institute for Modeling Collaboration and Innovation, The University of Idaho, Moscow, Idaho 83844, USA; b Department of Mathematical Sciences, New Mexico State University, Las Cruces, NM 88001, USA

**Keywords:** Ergodic invariant probability measure, Stochastic Lyapunov exponent, Hormander’s theorem, Exponential ergodicity, Stochastic Hopf bifurcation without parameters, Infection value

## Abstract

In part II, we analyze our stochastic model which incorporates microenvironmental noises and uncertainties related to immune responses. Outcomes of the therapy in our model are largely determined by the infectivity constant, the infection value, and stochastic relative immune clearance rates. The infection value is a universal critical value for immune-free ergodic invariant probability measures and persistence in all cases. Asymptotic behaviors of the stochastic model are similar to those of its deterministic counterpart. Our stochastic model displays an interesting dynamical behavior, stochastic Hopf bifurcation without parameters, which is a new phenomenon. We perform numerical study to demonstrate how stochastic Hopf bifurcation without parameters occurs. In addition, we give biological implications about our analytical results in stochastic setting versus deterministic setting.

## 1. Introduction

In part I [23], we proposed and analyzed a basic model for virotherapy which incorporates both innate and adaptive immune responses as following

(1) $$\frac{dx}{dt}=\lambda x\left(1-\frac{x+y}{C}\right)-\beta xy-k_{2}xz_{2},\;\frac{dy}{dt}=\beta xy-k_{1}yz_{1}-\delta y,\;\frac{dz_{1}}{dt}=s_{1}yz_{1}-c_{1}z_{1},\;\frac{dz_{2}}{dt}=s_{2}yz_{2}-c_{2}z_{2},$$

where *x* stands for the uninfected tumor population, *y* the infected tumor population, *z*_1_ and *z*_2_ the innate and adaptive immune cell populations, respectively. Tumor growth is modeled by logistic patterns with the growth rate *λ* and the carrying capacity *C*. The term *δy* represents the lysis rate of infected tumor cells. Our model does not include the free virus population explicitly. Release of virions by infected tumor cells and infection by free viruses are indirectly modeled by the term *βxy*. The anti-tumor adaptive immune response kills tumor cells at a rate *k*_2_ while the innate immune response kills infected tumor cells at a rate *k*_1_. Both innate and adaptive immune cells are stimulated through their interaction with infected tumor cells at rates of *s*_1_ and *s*_2_, and are cleared at rates of *c*_1_ and *c*_2_, respectively. Our analysis showed that the outcomes of the therapy are largely determined by the strength of viruses used in treatments which is captured by the parameter *β*, and the balance between the innate and adaptive immune cell recruitment ability through their interactions with infected tumor cells, which are represented by the ratios of clearance rate *c*_*i*_ to stimulation rate *s*_*i*_ of innate and adaptive immune cells (*i* = 1, 2). Specifically, the therapy can completely fail or partially succeed. For partial successes, the outcome can be immune free (without immune cells after a long period of time) or the outcome can have immune cells eventually. Our model also predicted three partially successful outcomes which have only innate immune cells, only adaptive immune cells, or have both innate and adaptive immune cells. For the outcome with tumor cells, infected tumor cells, and both innate and adaptive immune cells, called persistent state, the model predicted interesting phenomena, namely, Poincare-Andronov-Hopf bifurcations without parameters.

As explained in [23], we are concerned about how microenvironmental noises or uncertainties from immune responses will influence outcomes of the therapy in our model. We, therefore, proposed a system of Ito stochastic differential equations based on our deterministic model to incorporate microenvironmental noises and uncertainties as follows.

(2) $$dx=\left[\lambda x\left(1-\frac{x+y}{C}\right)-\beta xy-k_{2}xz_{2}\right]dt,\;dy=\left(\beta xy-k_{1}yz_{1}-\delta y\right)dt,\;dz_{1}=\left(s_{1}yz_{1}-c_{1}z_{1}\right)dt+\tau_{1}z_{1}dW_{1},\;dz_{2}=\left(s_{2}yz_{2}-c_{2}z_{2}\right)dt+\tau_{2}z_{2}dW_{2}.$$

In this part II, we analyze this stochastic model. In some sense, ergodic invariant probability measures in stochastic systems play similar roles as equilibrium states in deterministic systems. However, analyzing stochastic systems requires more and deeper knowledge from probability theory and other related theories. We use stochastic version of Lyapunov exponent theory [2,3] and boundary analysis [6,7,9]. Since our stochastic system is noise degenerated, to check hypoellipticity, we use Hörmander’s theorems [4,10,20]. To study ergodicity, for example, supports of invariant measures, we use geometric control theory [4,12,13]. Bifurcation theory for stochastic systems is still a developing area. In the book [1], there are two types of stochastic bifurcations. The first type is phenomenological bifurcation (or P-bifurcation), which is concerned with the change in the shape of density functions of a family of invariant probability measures in a stochastic system as one of its parameter changes. The second one is dynamical bifurcation (or D-bifurcation), which is characterized by sign changes of Lyapunov exponents of a family of invariant probability measures in a stochastic system as one of its parameter changes. As we know, so far, there is no theory or example about stochastic bifurcations without parameters. In general, bifurcations without parameters are ones that occur when state variables pass some values. We find our model undergoes stochastic Hopf bifurcations without parameters. This is the first stochastic system which has stochastic Poincare-Andronov-Hopf bifurcation without parameters.

The dynamical behaviors of our stochastic differential equation system correspond to those of its deterministic counterpart system. Particularly, the stochastic system has 5 ergodic invariant probability measures on the boundary of its almost sure invariant domain and a collection of invariant probability measures in the interior of its almost sure invariant domain. In a parallel manner, the deterministic system (2.1) in [23] has 5 equilibrium points on the boundary of its invariant domain, and a manifold of equilibria in the interior of its invariant domain. The stochastic subsystems also correspond to their deterministic counterparts. The stochastic model and their deterministic counterpart share similar asymptotic properties although in different settings. However, the stochastic system reveals more intrinsic properties of the therapy, for instance, the critical value for immune clearance rates, called the infection value, which is universal for partial successes without immune components.

The rest of this article is organized as follows. In section 2, we list main results and provide medical interpretations or implications. In Section 3, we present analysis to prove the main results. In section 4, we perform numerical studies to demonstrate stochastic bifurcation without parameters and discuss how the stochastic model helps to gain deep insights about tumor virotherapy. After Discussion, we give an Appendix to list theorems we cited related to hypoellipticity and Hörmander’s conditions, geometric control theory, and exponential ergodicity.

## 2. Notations and results

We non-dimensionalize the system (2) by setting $x=C\bar{x}$, *y* = *Cȳ*, $z_{1}=C{\bar{z}}_{1}$, $z_{2}=C{\bar{z}}_{2}$, $r=\frac{\lambda }{\delta }$, $a=\frac{\beta C}{\delta }$, $l_{i}=\frac{k_{i}C}{\delta }$, $e_{i}=\frac{s_{i}C}{\delta }$, $d_{i}=\frac{c_{i}}{\delta }$, and *T* = *δt*. After dropping all bars over the variables and writing *T* as *t*, the system (2) becomes

(3) $$dx=\left[rx(1-x-y)-axy-l_{2}xz_{2}\right]dt,\;dy=\left(axy-l_{1}yz_{1}-y\right)dt,\;dz_{1}=\left(e_{1}yz_{1}-d_{1}z_{1}\right)dt+\tau_{1}z_{1}dW_{1},\;dz_{2}=\left(e_{2}yz_{2}-d_{2}z_{2}\right)dt+\tau_{2}z_{2}dW_{2}.$$

Assume that we are working on a complete probability space $\left(\Omega ,ℱ,{\left\{{ℱ}_{t}\right\}}_{t\geq 0},\mathbb{P}\right)$ with a filtration {ℱ_*t*_}_*t*≥0_ satisfying the usual conditions. The process given by the solution of the system (3) will be denoted by *U* or *U*(*t*) = (*x*(*t*), *y*(*t*), *z*_1_(*t*), *z*_2_(*t*)), *t* ≥ 0. We denote the drift term and the diffusion term of the system (3), respectively, by

$$f(U)=\left[\begin{array}{c} rx(1-x-y)-axy-l_{2}xz_{2}\\ axy-l_{1}yz_{1}-y\\ e_{1}yz_{1}-d_{1}z_{1}\\ e_{2}yz_{2}-d_{2}z_{2} \end{array}\right],\text{ and }g(U)=\left[\begin{array}{cc} 0 & 0\\ 0 & 0\\ \tau_{1}z_{1} & 0\\ 0 & \tau_{2}z_{2} \end{array}\right].$$

Let ℒ be the infinitesimal generator of the process *U* and, for any smooth enough functions $F:{\mathbb{R}}_{+}^{4}\to \mathbb{R}$, the generator ℒ acts as

$$ℒF(U)≔F_{U}\cdot f(U)+\frac{1}{2}\text{trace}\left(g(U)g{\left(U\right)}^{T}F_{UU}\right)$$

where *F*_*U*_ is the gradient of *F* and *F*_*UU*_ is the Hessian matrix of *F*. We use ${\mathbb{P}}_{u}$ to denote the probability law on Ω when the solution path starts at *u* = (*x*, *y*, *z*_1_, *z*_2_) and $E_{u}$ is the expectation corresponding to ${\mathbb{P}}_{u}$.

It is straightforward to verify that the non-compact region

$$D=\left\{\left(x,y,z_{1},z_{2}\right):x\geq 0,y\geq 0,z_{1}\geq 0,z_{2}\geq 0,x+y\leq 1\right\}$$

is the a.s. (almost sure) non-negative invariant domain of the system (3) (for example, see [22]). We refer it as a global domain. To determine the dynamics, we define three biologically meaningful parameters, the stochastic relative innate immune clearance rate $h_{1}≔\frac{d_{1}}{e_{1}}+\frac{\tau_{1}^{2}}{2e_{1}}$, the stochastic relative adaptive immune clearance rate $h_{2}≔\frac{d_{2}}{e_{2}}+\frac{\tau_{2}^{2}}{2e_{2}}$, and the infection value $\theta ≔\frac{r(a-1)}{a(a+r)}$. Then, two Lyapunov exponents *λ*_*i*_ = *e*_*i*_(*θ*−*h*_*i*_), *i* = 1, 2. We also define *λ* = *θ*−*h* which is proportional to a Lyapunov exponent when *h*_1_ = *h*_2_ ≕ *h*. It turns out that there are three cases which we should consider as in the deterministic system.

**Case 1**. When *h*_1_ < *h*_2_, the adaptive immune cell population *z*_2_(*t*) decays to 0 a.s. as *t* → ∞, and so the 4-dimensional system (3) is reduced to the 3-dimensional SDE system

(4) $$dx=[rx(1-x-y)-axy]dt,\;dy=\left(axy-l_{1}yz_{1}-y\right)dt,\;dz_{1}=\left(e_{1}yz_{1}-d_{1}z_{1}\right)dt+\tau_{1}z_{1}dW_{1},$$

where *D*_1_ = {(*x*, *y*, *z*_1_): *x* ≥ 0, *y* ≥ 0, *z*_1_ ≥ 0, *x* + *y* ≤ 1} is its almost sure non-negative invariant domain. For the system (4), we work on a complete probability space $\left(\Omega^{1},{ℱ}^{1},{\left\{{ℱ}_{t}^{1}\right\}}_{t\geq 0},\mathbb{P}\right)$ (which is the projection of the complete probability space $\left(\Omega ,ℱ,{\left\{{ℱ}_{t}\right\}}_{t\geq 0},\mathbb{P}\right)$ on *z*_2_ = 0). With $u_{1}≔\left(x,y,z_{1}\right)\in D_{1}^{\circ }$ (the interior of *D*_1_), we denote by *U*_1_ or $U_{1}^{u_{1}}(t)≔\left(x(t),y(t),z_{1}(t)\right)$ the solution to the system (4) starting at *u*_1_. Our analysis in Section 3 indicates that there are 3 ergodic invariant measures for the system (4) on the boundary *∂D*_1_

$${\bar{\mu }}_{0}=\delta_{0}^{*}\times \delta_{0}^{*}\times \delta_{0}^{*},{\bar{\mu }}_{1}=\delta_{1}^{*}\times \delta_{0}^{*}\times \delta_{0}^{*},\text{ and }{\bar{\mu }}_{2}=\delta_{x_{1}^{*}}^{*}\times \delta_{y_{1}^{*}}^{*}\times \delta_{0}^{*}$$

where $x_{1}^{*}≔\frac{1}{a}$ and $y_{1}^{*}≔\frac{r(a-1)}{a(a+r)}$. Here $\delta_{0}^{*}$, $\delta_{1}^{*}$, $\delta_{x_{1}^{*}}^{*}$, and $\delta_{y_{1}^{*}}^{*}$ are Dirac measures with mass at 0, 1, $x_{1}^{*}$, and $y_{1}^{*}$, respectively. The complete picture of the stochastic dynamics of the system (4) is determined by the infectivity constant *a* and the parameter *λ*_1_, which is summarized in the following theorem.

**Theorem 2.1**. *Under the assumption h*_1_ < *h*_2_, *the long-term behaviors of the system* (3) *on the invariant domain D can be reduced to that of the system* (4) *on the invariant domain D*_1_. *With any initial condition u*_1_ = (*x*, *y*, *z*_1_) ∈ *D*_1_, *the system* (4) *has a unique a.s*. *continuous solution* $U_{1}^{u_{1}}(t)$ *that remains in D*_1_ *for all t* ≥ 0 *a.s*. *Also*, $U_{1}^{u_{1}}(t)$ *is a strong Markov process that possesses the Feller property*.

*On ∂D*_1_, *the system* (4) *has 3 ergodic invariant probability measures* ${\bar{\mu }}_{0}$, ${\bar{\mu }}_{1}$, *and* ${\bar{\mu }}_{2}$*; in* $D_{1}^{\circ }$ *the system* (4) *has a unique invariant probability measure* ${\bar{\mu }}_{3}$.

- ${\bar{\mu }}_{0}$ *is always a repeller for all values of a*.
- *If* 0 < *a* < 1, *then the system* (4) *has 2 ergodic invariant probability measures* ${\bar{\mu }}_{0}$ *and* ${\bar{\mu }}_{1}$ *on the boundary ∂D*_1_ *in which* ${\bar{\mu }}_{1}$ *is a global attractor*.
- *If a* > 1 *and λ*_1_ < 0, *then the system* (4) *has 3 ergodic invariant probability measures* ${\bar{\mu }}_{0}$, ${\bar{\mu }}_{1}$, *and* ${\bar{\mu }}_{2}$ *on the boundary ∂D*_1_ *where* ${\bar{\mu }}_{0}$ *and* ${\bar{\mu }}_{1}$ *are repellers and* ${\bar{\mu }}_{2}$ *is a global attractor*.
- *If a* > 1 *and λ*_1_ > 0, *then*, *besides* ${\bar{\mu }}_{0}$, ${\bar{\mu }}_{1}$, *and* ${\bar{\mu }}_{2}$, *there exists a unique invariant probability measure* ${\bar{\mu }}_{3}$ *in* $D_{1}^{\circ }$ *supported by the open line segment*
$$S_{1}≔\left\{\left(\frac{l_{1}z_{1}+1}{a},\frac{r\left(a-1-l_{1}z_{1}\right)}{a(a+r)},z_{1}\right):z_{1}\in \left(0,\frac{a-1}{l_{1}}\right)\right\}$$
  *and the solution* $U_{1}^{u_{1}}(t)$ *is exponentially ergodic with respect to* ${\bar{\mu }}_{3}$ *in the sense that the transition probability of the solution* $U_{1}^{u_{1}}(t)$ *converges to* ${\bar{\mu }}_{3}$ *exponentially in total variation norm*.

**Case 2**. When *h*_1_ > *h*_2_, the innate immune cell population *z*_1_(*t*) decays to 0 a.s. as *t* → ∞ and so the system (3) is reduced to the 3-dimensional SDE system

(5) $$dx=\left[rx(1-x-y)-axy-l_{2}xz_{2}\right]dt,\;dy=(axy-y)dt,\;dz_{2}=\left(e_{2}yz_{2}-d_{2}z_{2}\right)dt+\tau_{2}z_{2}dW_{2},$$

where *D*_2_ = {(*x*, *y*, *z*_2_): *x* ≥ 0, *y* ≥ 0, *z*_2_ ≥ 0, *x* + *y* ≤ 1} is its almost sure non-negative invariant domain. For the system (5), we work on a complete probability space $\left(\Omega^{2},{ℱ}^{2},{\left\{{ℱ}_{t}^{2}\right\}}_{t\geq 0},\mathbb{P}\right)$ (which is the projection of the complete probability space $\left(\Omega ,ℱ,{\left\{{ℱ}_{t}\right\}}_{t\geq 0},\mathbb{P}\right)$ on *z*_1_ = 0. With $u_{2}≔\left(x,y,z_{2}\right)\in D_{2}^{\circ }$ (the interior of *D*_2_), we denote by *U*_2_ or $U_{2}^{u_{2}}(t)≔\left(x(t),y(t),z_{2}(t)\right)$ the solution to the system (5) starting at *u*_2_. By the analysis in Section 3, there are 3 ergodic invariant measures for the system (5) on the boundary *∂D*_2_

$${\tilde{\mu }}_{0}=\delta_{0}^{*}\times \delta_{0}^{*}\times \delta_{0}^{*},{\tilde{\mu }}_{1}=\delta_{1}^{*}\times \delta_{0}^{*}\times \delta_{0}^{*},\text{ and }{\tilde{\mu }}_{2}=\delta_{x_{1}^{*}}^{*}\times \delta_{y_{1}^{*}}^{*}\times \delta_{0}^{*}.$$

With the parameters *a* and *λ*_2_, the complete dynamics of the system (5) is stated in the following theorem.

**Theorem 2.2**. *Assume that h*_1_ > *h*_2_. *The long-term dynamics of the system* (3) *on the invariant domain D is governed by that of the system* (5) *on the invariant domain D*^2^. *With any initial condition u*_2_ = (*x*, *y*, *z*_2_) ∈ *D*_2_, *the system* (5) *has a unique a.s*. *continuous solution* $U_{2}^{u_{2}}(t)$ *that remains in D*_2_ *for all t* ≥ 0 *a.s*. *Also*, $U_{2}^{u_{2}}(t)$ *is a strong Markov process that possesses the Feller property*.

*On ∂D*_2_, *the system* (5) *has 3 ergodic invariant probability measures* ${\tilde{\mu }}_{0}$, ${\tilde{\mu }}_{1}$, *and* ${\tilde{\mu }}_{2}$*; in* $D_{2}^{\circ }$ *the system* (5) *has a unique invariant probability measure* ${\tilde{\mu }}_{4}$.

- ${\tilde{\mu }}_{0}$ *is always a repeller for all values of a*.
- *If* 0 < *a* < 1, *then the system* (5) *has 2 ergodic invariant probability measures* ${\tilde{\mu }}_{0}$ *and* ${\tilde{\mu }}_{1}$ *on the boundary ∂D*_2_ *in which* ${\tilde{\mu }}_{1}$ *is a global attractor*.
- *If a* > 1 *and λ*_2_ < 0, *then the system* (5) *has 3 ergodic invariant probability measures* ${\tilde{\mu }}_{0}$, ${\tilde{\mu }}_{1}$, *and* ${\tilde{\mu }}_{2}$ *on the boundary ∂D*_2_ *where* ${\tilde{\mu }}_{0}$ *and* ${\tilde{\mu }}_{1}$ *are repellers and* ${\tilde{\mu }}_{2}$ *is a global attractor*.
- *If a* > 1 *and λ*_2_ > 0, *then*, *besides* ${\tilde{\mu }}_{0}$, ${\tilde{\mu }}_{1}$, *and* ${\tilde{\mu }}_{2}$, *there exists a unique invariant probability measure* ${\tilde{\mu }}_{4}$ *in* $D_{2}^{\circ }$ *supported by the open line segment*
$$S_{2}≔\left\{\left(\frac{1}{a},\frac{r(a-1)}{a(a+r)}-\frac{l_{2}z_{2}}{a+r},z_{2}\right):z_{2}\in \left(0,\frac{r(a-1)}{al_{2}}\right)\right\}$$
  *and the solution* $U_{2}^{u_{2}}(t)$ *is exponentially ergodic with respect to* ${\tilde{\mu }}_{4}$ *in the sense that the transition probability of the solution* $U_{2}^{u_{2}}(t)$ *converges to* ${\tilde{\mu }}_{4}$ *exponentially in total variation norm*.

**Case 3.** When *h*_1_ = *h*_2_, both types of immune responses are stimulated simultaneously and coexist as time goes by. Under certain conditions, there exists a collection of invariant probability measures in *D*° such that the solution of the system (3) is exponentially ergodic with respect to each of these measures. This interesting property is similar to the Poincare-Andronov-Hopf bifurcation without parameters that the deterministic counterpart system of (3) undergoes (see part I [23]). The results are stated in the following theorem.

**Theorem 2.3**. *Suppose that h*_1_ = *h*_2_ ≕ *h*. *On the boundary ∂D*, *the system* (3) *has 5 ergodic invariant probability measures*

$\mu_{0}=\delta_{0}^{*}\times \delta_{0}^{*}\times \delta_{0}^{*}\times \delta_{0}^{*}$, $\mu_{1}=\delta_{1}^{*}\times \delta_{0}^{*}\times \delta_{0}^{*}\times \delta_{0}^{*}$, $\mu_{2}=\delta_{x_{1}^{*}}^{*}\times \delta_{y_{1}^{*}}^{*}\times \delta_{0}^{*}\times \delta_{0}^{*}$ *μ*_3_ *on* {*z*_2_ = 0} *supported by*

$$S(0)≔\left\{\left(\frac{l_{1}z_{1}+1}{a},\frac{r\left(a-1-l_{1}z_{1}\right)}{a(a+r)},z_{1},0\right):z_{1}\in \left(0,\frac{a-1}{l_{1}}\right)\right\},$$

*μ*_4_ *on* {*z*_1_ = 0} *supported by*

$$S(\infty ):=\left\{\left(\frac{1}{a},\frac{r(a-1)}{a(a+r)}-\frac{l_{2}z_{2}}{a+r},0,z_{2}\right):z_{2}\in \left(0,\frac{r(a-1)}{al_{2}}\right)\right\}.$$

*The complete dynamics of the system* (3) *is determined by the parameters a and λ*.

- *μ*_0_ *is always a repeller for all values of a*.
- *If* 0 < *a* < 1, *then the system* (3) *has only 2 ergodic invariant probability measures μ*_0_ *and μ*_1_ *on ∂D in which μ*_1_ *is a global attractor*.
- *If a* > 1 *and λ* < 0, *then the system* (3) *has only 3 ergodic invariant probability measures μ*_0_, *μ*_1_, *and μ*_2_ *on ∂D where μ*_1_ *is a repeller and μ*_2_ *is a global attractor*.
- *If a* > 1 *and λ* > 0, *then*, *besides μ*_0_, *μ*_1_, *and μ*_2_, *there exists a collection of invariant probability measures* {*π*(*k*)}_k∈[0,∞]_ *for the system* (3). *For each k* ∈ (0, ∞), *π*(*k*) *is supported by*
$$S(k)≔\left\{\left(\frac{l_{1}z_{1}+1}{a},\frac{r\left(a-1-l_{1}z_{1}\right)}{a(a+r)}-\frac{kl_{2}z_{1}^{\rho }}{a+r},z_{1},kz_{1}^{\rho }\right):z_{1}\in \left(0,\frac{a-1}{l_{1}}\right)\right\},$$
  *where* $\rho =\frac{e_{2}}{e_{1}}$, *and the solution U*^*u*^(*t*) *of the system* (3) *is exponentially ergodic with respect to π*(*k*) *whenever the initial value u is in D*° ∩*P*_*k*_*; here P*_*k*_ *denotes the invariant surface* $z_{2}=kz_{1}^{\rho }$. *When k* = 0, *π*(0) ≡ *μ*_3_ *and U*^*u*^(*t*) *is exponentially ergodic with respect to μ*_3_ *in the interior of D* ∩ {*z*_2_ = 0}. *When k* = ∞, *π*(∞) ≡ *μ*_4_ *and U*^*u*^(*t*) *is exponentially ergodic with respect to μ*_4_ *in the interior of D*∩{*z*_1_ = 0}.

**Interpretation 2.1**. *The dynamical behaviors of our stochastic differential equation system correspond to those of its deterministic counterpart system* [23] *as our notations indicate*. *Particularly*, *the system* (3) *has 5 ergodic invariant probability measures on the boundary of its almost sure invariant domain*, *μ*_0_, *μ*_1_, *μ*_2_, *μ*_3_, *μ*_4_, *and a collection of invariant probability measures* {*π*(*k*)}_*k*∈[0,∞]_ *in the interior of its almost sure invariant domain*. *In a parallel manner*, *the deterministic system* (*2.1*) *in* [23] *has 5 equilibrium points on the boundary of its invariant domain*, *E*_0_, *E*_1_, *E*_2_, *E*_3_, *E*_4_, *and a manifold of equilibria M in the interior of its invariant domain*. *The stochastic subsystems also correspond to their deterministic counterparts*. *Importantly*, *the stochastic model and their deterministic counterpart share similar asymptotic properties although in different settings*.

*It is reasonable that the ergodic invariant probability measures μ*_0_, ${\bar{\mu }}_{0}$, *and* ${\tilde{\mu }}_{0}$ *are always repellers for any positive parameter values*. *Since we only consider noises and uncertainties related to immune cells*, *these uncertainties do not affect the infectivity constant a*. *So*, *as interpreted in* [23], *the ergodic invariant probability measures μ*_1_, ${\bar{\mu }}_{1}$, *and* ${\tilde{\mu }}_{1}$ *are global attractors when* 0 < *a* < 1. *That means the therapy completely fails*. *When a* > 1, *our stochastic model predicts two partial successes for the virotherapy as its deterministic counterpart*, *one is immune free*, *and another one has immune components*. *However*, *the conditions to distinguish these two partial successes are Lyapunov exponents*, *which have medical implications*.

*The stochastic relative immune clearance rates* $h_{i}=\frac{d_{i}}{e_{i}}+\frac{\tau_{i}^{2}}{2e_{i}}(i=1,2)$ *play the similar role as the relative immune clearance rates* $\frac{d_{i}}{e_{i}}(i=1,2)$ *in classifying the overall dynamics*. *However*, *the stochastic relative immune clearance rates are the sum of the relative immune clearance rate and a term containing the uncertainty variance* $\tau_{i}^{2}$ *made by each immune cells or received by each immune cell from their microenvironment*. *This also contributes to our understanding about robustness of the virotherapy outcomes obtained from the deterministic model*. *As interpretations in the deterministic model*, *according to the relation between two stochastic relative immune clearance rates*, *our stochastic model is reduced to three sub-models*. *When h*_1_ < *h*_2_, *our stochastic model is reduced to a subsystem without adaptive immune cells* (4). *The Lyapunov exponent λ*_1_ < 0 *is equivalent to h*_1_ > *θ*. *If we consider the infection value θ to be a fixed value which is determined by the infectivity constant and tumor growth rate*, *then*, *when the stochastic relative innate immune clearance rate is greater than this fixed value*, *the innate immune cell population will eventually be cleared out*. *This is the case where the therapy reaches the immune-free ergodic invariant probability measure* ${\bar{\mu }}_{2}$. *The Lyapunov exponent λ*_1_ > 0 *is equivalent to h*_1_ < *θ*. *Therefore*, *the subsystem reaches the ergodic invariant probability measure* ${\bar{\mu }}_{3}$ *with innate immune components in its support*. *When h*_1_ > *h*_2_, *our stochastic model is reduced to a subsystem without innate immune cells* (5). *Similarly*, *when λ*_2_ < 0, *that is*, *h*_2_ > *θ*, *the adaptive immune cells will eventually be cleared out*, *and the subsystem reaches the immune-free ergodic invariant probability measure* ${\tilde{\mu }}_{2}$. *When λ*_2_ > 0, *that is*, *h*_2_ < *θ*, *the sub-system reaches the ergodic invariant probability measure* ${\tilde{\mu }}_{4}$ *with adaptive immune components in its support*. *When h*_1_ = *h*_2_, *we work on the full system*. *If λ* < 0, *meaning that h* > *θ*, *the system will reach the immune-free ergodic invariant probability measure μ*_2_. *If λ* > 0, *meaning that h* < *θ*, *the system will undergo stochastic Poincare-Andronov-Hopf bifurcation without parameters*. *We can see that the infection value θ is a universal critical value for understanding long-term behaviors and outcomes of the virotherapy*. *This value only is revealed in stochastic setting*.

## 3. Analysis of the model

This section is devoted to proving results in Section 2. Before giving the detailed proofs of the three main Theorems 2.1, 2.2, and 2.3, at first we do boundary analysis for the system (3). The purpose of this analysis is to investigate the set of ergodic invariant probability measures of the system (3) when its solutions start in *∂D*.

**A.** If *x*(0) = 0, then by the first equation of (3), *x*(*t*) ≡ 0 a.s. The second equation of (3) becomes *dy* = (−*l*_1_*yz*_1_ − *y*)*dt*, which follows that *y*(*t*) → 0 a.s. as *t* → ∞. By standard arguments, the long-term behavior of (3) is reduced to that of the following system

(6) $$dz_{1}=-d_{1}z_{1}dt+\tau_{1}z_{1}dW_{1},\;dz_{2}=-d_{2}z_{2}dt+\tau_{2}z_{2}dW_{2}.$$

This system is equivalent to

$$z_{1}(t)=z_{1}(0)\text{ exp }\left\{\left(-d_{1}-\tau_{1}^{2}/2\right)t+\tau_{1}W_{1}(t)\right\},$$

$$z_{2}(t)=z_{2}(0)\text{ exp }\left\{\left(-d_{2}-\tau_{2}^{2}/2\right)t+\tau_{2}W_{2}(t)\right\}.$$

So *z*_1_(*t*) → 0 a.s. and *z*_2_(*t*) → 0 a.s. as *t* → ∞. Thus, when the solution of (3) starts in {*x* = 0} ⊂ *∂D*, it converges to (0, 0, 0, 0) a.s. It follows that the transition probability of the solution *U*^*u*^(*t*) starting in {*x* = 0} ⊂ *∂D* converges to the ergodic invariant probability measure $\mu_{0}=\delta_{0}^{*}\times \delta_{0}^{*}\times \delta_{0}^{*}\times \delta_{0}^{*}$ in total variation norm.

**B.** Assume that *x*(0) > 0. By the first equation of (3), *x*(*t*) > 0 for all *t* ≥ 0 a.s. If *y*(0) = 0, then the second equation of (3) implies *y*(*t*) ≡ 0 a.s. Then, the last two equations of (3) become the system (6). By the same argument as above, *z*_1_(*t*) → 0 a.s. and *z*_2_(*t*) → 0 a.s. as *t* → ∞. So the long-term behavior of (3) is reduced to that of the equation *dx* = *rx*(1 − *x*)*dt* with initial condition *x*(0) > 0. It is easy to show that *x*(*t*) → 1 a.s. as *t* → ∞. So the transition probability of the solution *U*^*u*^(*t*) starting in {*y* = 0} ⊂ *∂D* converges to the ergodic invariant probability measure $\mu_{1}=\delta_{1}^{*}\times \delta_{0}^{*}\times \delta_{0}^{*}\times \delta_{0}^{*}$ in total variation norm.

**C.** Assume that *x*(0) > 0 and *y*(0) > 0. By the first two equations of (3), we get *x*(*t*) > 0 for all *t* ≥ 0 a.s. and *y*(*t*) > 0 for all *t* ≥ 0 a.s. To study long-term behaviors of (3), we look at the following 4 cases.

**C1.** If *z*_1_(0) = *z*_2_(0) = 0, then the last two equations of (3) imply *z*_1_(*t*) ≡ 0 a.s. and *z*_2_(*t*) ≡ 0 a.s. The long-term behavior of (3) is the same as that of the following system

(7) dx=[rx(1−x−y)−axy]dt,dy=(axy−y)dt.

It is straightforward that the a.s. non-negative invariant domain of this system is

Δ={(x,y):x≥0,y≥0,x+y≤1}.

The long-term behavior of (7) in Δ° depends on the parameter *a*. If 0 < *a* < 1 then, by the second equation of (7), *dy* ≤ (*a* − 1)*ydt* which implies that 0 ≤ *y*(*t*) ≤ *y*(0) exp{(*a* − 1)*t*} a.s. Since 0 < *a* < 1, *y*(*t*) → 0 a.s. as *t* → ∞. By standard arguments, the long-term behavior of (7) is reduced to that of the equation *dx* = *rx*(1 − *x*)*dt* with initial condition *x*(0) > 0. Hence *x*(*t*) → 1 a.s. as *t* → ∞. Therefore the transition probability of the solution *U*^*u*^(*t*) starting in {*z*_1_ = 0, *z*_2_ = 0} ⊂ *∂D* converges to *μ*_1_ in total variation norm. If *a* > 1, we consider the function

$$V_{1}(x,y)=x-x_{1}^{*}-x_{1}^{*}\text{ log }\frac{x}{x_{1}^{*}}+\frac{r+a}{a}\left(y-y_{1}^{*}-y_{1}^{*}\text{ log }\frac{y}{y_{1}^{*}}\right)$$

where $\left(x_{1}^{*},y_{1}^{*}\right)≔\left(\frac{1}{a},\frac{r(a-1)}{a(a+r)}\right)\in \Delta^{\circ }$. It is easy to check that $\frac{dV_{1}}{dt}=-r{\left(x-x_{1}^{*}\right)}^{2}\leq 0$. Using Lasalle’s principle, we can conclude that $(x(t),y(t))\to \left(x_{1}^{*},y_{1}^{*}\right)$ a.s. as *t* → ∞. Thus, the transition probability of the solution *U*^*u*^(*t*) starting in {*z*_1_ = 0, *z*_2_ = 0} converges to the ergodic invariant probability measure $\mu_{2}=\delta_{x_{1}^{*}}^{*}\times \delta_{y_{1}^{*}}^{*}\times \delta_{0}^{*}\times \delta_{0}^{*}$ in total variation norm.

**C2.** Assume that *z*_1_(0) > 0 and *z*_2_(0) = 0. By the last two equations of (3), *z*_1_(*t*) > 0 for all *t* ≥ 0 a.s. and *z*_2_(*t*) ≡ 0 a.s. This implies that the long-term behavior of (3) in *D* is the same as that of (4) in *D*_1_. By boundary analysis for the system (4) on the boundary *∂D*_1_, we obtain the following

- If the solution $U_{1}^{u_{1}}(t)$ of (4) starts in {*x* = 0} ⊂ *∂D*_1_, then its transition probability converges to ${\bar{\mu }}_{0}=\delta_{0}^{*}\times \delta_{0}^{*}\times \delta_{0}^{*}$ in total variation norm.
- If the solution $U_{1}^{u_{1}}(t)$ of (4) starts in {*y* = 0} ⊂ *∂D*_1_, then its transition probability converges to ${\bar{\mu }}_{1}=\delta_{1}^{*}\times \delta_{0}^{*}\times \delta_{0}^{*}$ in total variation norm.
- If the solution $U_{1}^{u_{1}}(t)$ of (4) starts in {*z*_1_ = 0} ⊂ *∂D*_1_, then its transition probability converges to ${\bar{\mu }}_{2}=\delta_{x_{1}^{*}}^{*}\times \delta_{y_{1}^{*}}^{*}\times \delta_{0}^{*}$ in total variation norm.

Now assume that the initial value *u*_1_ of the solution $U_{1}^{u_{1}}(t)$ is in $D_{1}^{\circ }$, the interior of *D*_1_. To investigate the long-term behavior of the solution $U_{1}^{u_{1}}(t)$ starting in $D_{1}^{\circ }$, we compute the Lyapunov exponents of the ergodic invariant probability measures ${\bar{\mu }}_{0}$, ${\bar{\mu }}_{1}$, and ${\bar{\mu }}_{2}$ of (4) on the boundary *∂D*_1_

$$\lambda_{1}\left({\bar{\mu }}_{0}\right)=r,\;\lambda_{1}\left({\bar{\mu }}_{1}\right)=0,\;\lambda_{1}\left({\bar{\mu }}_{2}\right)=0,$$

$$\lambda_{2}\left({\bar{\mu }}_{0}\right)=-1,\;\lambda_{2}\left({\bar{\mu }}_{1}\right)=a-1,\;\lambda_{2}\left({\bar{\mu }}_{2}\right)=0,$$

$$\lambda_{3}\left({\bar{\mu }}_{0}\right)=-d_{1}-\frac{\tau_{1}^{2}}{2},\;\lambda_{3}\left({\bar{\mu }}_{1}\right)=-d_{1}-\frac{\tau_{1}^{2}}{2},\;\lambda_{3}\left({\bar{\mu }}_{2}\right)=e_{1}y_{1}^{*}-d_{1}-\frac{\tau_{1}^{2}}{2}≕\lambda_{1}.$$

Since $\lambda_{1}\left({\bar{\mu }}_{0}\right)>0$, ${\bar{\mu }}_{0}$ is always a repeller in the sense that whenever the solution $U_{1}^{u_{1}}(t)$ is close to the support of ${\bar{\mu }}_{0}$ (which is $\text{supp}\left({\bar{\mu }}_{0}\right)=\left\{\left(0,0,0\right)\right\}$), it goes away. If 0 < *a* < 1 then, from the second equation of (4), we can easily show that *y*(*t*) → 0 a.s. as *t* → ∞. The behavior of the last equation of (4) is the same as that of the equation *dz*_1_ = −*d*_1_*z*_1_*dt* + *τ*_1_*z*_1_*dW*_1_, which follows that *z*_1_(*t*) → 0 a.s. as *t* → ∞. By the first equation of (4), the behavior of *x*(*t*) is determined by the equation *dx* = *rx*(1 − *x*)*dt*. Hence *x*(*t*) → 1 a.s. as *t* → ∞. Thus the transition probability of the solution $U_{1}^{u_{1}}(t)\;$ starting in $D_{1}^{\circ }\;$ converges to ${\bar{\mu }}_{1}$ in total variation norm. Next, we suppose that *a* > 1. Then the long-term behavior of the solution $U_{1}^{u_{1}}(t)$ is determined by the combined parameter *λ*_1_, which is stated in the following two theorems whose proofs will be given in Subsection 3.1.

**Theorem 3.1**. *Let* $U_{1}^{u_{1}}(t)$ *be the solution to the system* (4) *with initial condition u*_1_ *in* $D_{1}^{\circ }$. *Assume that a* > 1 *and λ*_1_ > 0. *Then*

- There exists a unique invariant probability measure ${\bar{\mu }}_{3}$ supported by
$$S_{1}≔\left\{\left(\frac{l_{1}z_{1}+1}{a},\frac{r\left(a-1-l_{1}z_{1}\right)}{a(a+r)},z_{1}\right):z_{1}\in \left(0,\frac{a-1}{l_{1}}\right)\right\}.$$
- *There are a γ* > 0 *and a positive function* $H\left(u_{1}\right):D_{1}^{\circ }\to {\mathbb{R}}_{+}$ *such that*
$${\left\|P\left(t,u_{1},\cdot \right)-{\bar{\mu }}_{3}\left(\cdot \right)\right\|}_{TV}\leq H\left(u_{1}\right)e^{-\gamma t}$$
  *for all t* ≥ 0 *and for all* $u_{1}\in D_{1}^{\circ }$ *in which* ∥·∥_*TV*_ *is the total variation norm and P*(*t*, *u*_1_, ·) *is the transition probability of the solution* $U_{1}^{u_{1}}(t)$. *That is*, $U_{1}^{u_{1}}(t)$ *is exponentially ergodic with respect to* ${\bar{\mu }}_{3}$.
- *Furthermore*, *for all* ${\bar{\mu }}_{3}$-*integrable function f and for all* $u_{1}\in D_{1}^{\circ }$ *we get*
$$\underset{t\to \infty }{\text{lim}}\;\frac{1}{t}\int_{0}^{t}f\left(U_{1}^{u_{1}}(s)\right)ds=\int_{D_{1}^{\circ }}f\left(u_{1}\right){\bar{\mu }}_{3}\left(du_{1}\right)\;a.s.$$
  *This is called the strong law of large number for* ${\bar{\mu }}_{3}$.

**Theorem 3.2**. *Let* $U_{1}^{u_{1}}(t)$ *be the solution to the system* (4) *with initial condition u*_1_ *in* $D_{1}^{\circ }$. *Assume that a* > 1 *and λ*_1_ < 0. *Then* $U_{1}^{u_{1}}(t)\to \left(x_{1}^{*},y_{1}^{*},0\right)$ *a.s*. *as t* → ∞. *Furthermore*,

$$\underset{t\to \infty }{\text{lim}}\;\frac{\text{log }z_{1}(t)}{t}=\lambda_{1}<0\;a.s.$$

*for any initial condition* $u_{1}\in D_{1}^{\circ }$.

**C3.** Suppose that *z*_1_(0) = 0 and *z*_2_(0) > 0. By the last two equations of (3), *z*_1_(*t*) ≡ 0 a.s. and *z*_2_(*t*) > 0 for all *t* ≥ 0 a.s. This follows that the long-term behavior of (3) in *D* is the same as that of (5) in *D*_2_. By analyzing the system (5) on the boundary *∂D*_2_, we obtain the following

- If the solution $U_{2}^{u_{2}}(t)$ of (5) starts in {*x* = 0} ⊂ *∂D*_2_, then its transition probability converges to ${\tilde{\mu }}_{0}=\delta_{0}^{*}\times \delta_{0}^{*}\times \delta_{0}^{*}$ in total variation norm.
- If the solution $U_{2}^{u_{2}}(t)$ of (5) starts in {*y* = 0} ⊂ *∂D*_2_, then its transition probability converges to ${\tilde{\mu }}_{1}=\delta_{1}^{*}\times \delta_{0}^{*}\times \delta_{0}^{*}$ in total variation norm.
- If the solution $U_{2}^{u_{2}}(t)$ of (5) starts in {*z*_2_ = 0} ⊂ *∂D*_2_, then its transition probability converges to ${\tilde{\mu }}_{2}=\delta_{x_{1}^{*}}^{*}\times \delta_{y_{1}^{*}}^{*}\times \delta_{0}^{*}$ in total variation norm.

Now suppose that the initial value *u*_2_ of the solution $U_{2}^{u_{2}}(t)$ is in $D_{2}^{\circ }$, the interior of *D*_2_. To look into the long-term behavior of the solution $U_{2}^{u_{2}}(t)$ starting in $D_{2}^{\circ }$, we calculate the Lyapunov exponents of the ergodic invariant probability measures ${\tilde{\mu }}_{0}$, ${\tilde{\mu }}_{1}$, and ${\tilde{\mu }}_{2}$ of (5) on the boundary *∂D*_2_

$$\begin{array}{c} \lambda_{1}\left({\tilde{\mu }}_{0}\right)=r,\;\lambda_{1}\left({\tilde{\mu }}_{1}\right)=0,\;\lambda_{1}\left({\tilde{\mu }}_{2}\right)=0, \end{array}$$

$$\lambda_{2}\left({\tilde{\mu }}_{0}\right)=-1,\;\lambda_{2}\left({\tilde{\mu }}_{1}\right)=a-1,\;\lambda_{2}\left({\tilde{\mu }}_{2}\right)=0,$$

$$\lambda_{3}\left({\tilde{\mu }}_{0}\right)=-d_{2}-\frac{\tau_{2}^{2}}{2},\;\lambda_{3}\left({\tilde{\mu }}_{1}\right)=-d_{2}-\frac{\tau_{2}^{2}}{2},\;\lambda_{3}\left({\tilde{\mu }}_{2}\right)=e_{2}y_{1}^{*}-d_{2}-\frac{\tau_{2}^{2}}{2}≕\lambda_{2}.$$

Since $\lambda_{1}\left({\tilde{\mu }}_{0}\right)>0$, ${\tilde{\mu }}_{0}$ is always a repeller in the sense that whenever the solution $U_{2}^{u_{2}}(t)$ gets close to the support of ${\tilde{\mu }}_{0}$ (which is $\text{supp}\left({\tilde{\mu }}_{0}\right)=\left\{\left(0,0,0\right)\right\}$), it repels away from the boundary *∂D*_2_. If 0 < *a* < 1 then, by the same arguments as in C2, the transition probability of the solution $U_{2}^{u_{2}}(t)$ starting in $D_{2}^{\circ }$ converges to ${\tilde{\mu }}_{1}$ in total variation norm. Next, we suppose that *a* > 1. Then the long-term behavior of the solution $U_{2}^{u_{2}}(t)$ is determined by the combined parameter *λ*_2_, which is summarized in the following two theorems whose proofs will be given in Subsection 3.2.

**Theorem 3.3.** *Let* $U_{2}^{u_{2}}(t)$ *be the solution to the system* (5) *with initial condition u*_2_ *in* $D_{2}^{\circ }$. *Assume that a* > 1 *and λ*_2_ > 0. *Then*

- There exists a unique invariant probability measure ${\tilde{\mu }}_{4}$ supported by
$$S_{2}≔\left\{\left(\frac{1}{a},\frac{r(a-1)}{a(a+r)}-\frac{l_{2}z_{2}}{a+r},z_{2}\right):z_{2}\in \left(0,\frac{r(a-1)}{al_{2}}\right)\right\}.$$
- *There are a η* > 0 *and a positive function* $H\left(u_{2}\right):D_{2}^{\circ }\to {\mathbb{R}}_{+}$ *such that*
$${\left\|P\left(t,u_{2},\cdot \right)-{\tilde{\mu }}_{4}(\cdot )\right\|}_{TV}\leq H\left(u_{2}\right)e^{-\eta t}$$
  *for all t* ≥ 0 *and for all* $u_{2}\in D_{2}^{\circ }$ *in which* ∥·∥_*TV*_ *is the total variation norm and P*(*t*, *u*_2_, ·) *is the transition probability of the solution* $U_{2}^{u_{2}}(t)$. *That is*, $U_{2}^{u_{2}}(t)$ *is exponentially ergodic with respect to* ${\tilde{\mu }}_{4}$.
- *Furthermore*, *for all* ${\tilde{\mu }}_{4}$-*integrable function f and for all* $u_{2}\in D_{2}^{\circ }$ *we get*
$$\underset{t\to \infty }{\text{lim}}\frac{1}{t}\int_{0}^{t}f\left(U_{2}^{u_{2}}(s)\right)ds=\underset{D_{2}^{\circ }}{\int }f\left(u_{2}\right){\tilde{\mu }}_{4}\left(du_{2}\right)\;a.s.\;$$
  *This is called the strong law of large number for* ${\tilde{\mu }}_{4}$.

**Theorem 3.4**. *Let* $U_{2}^{u_{2}}(t)$ *be the solution to the system* (5) *with initial condition u*_2_ *in* $D_{2}^{\circ }$. *Assume that a* > 1 *and λ*_2_ < 0. *Then* $U_{2}^{u_{2}}(t)\to \left(x_{1}^{*},y_{1}^{*},0\right)$ *a.s*. *as t* → ∞. *Furthermore*,

$$\underset{t\to \infty }{\text{lim}}\frac{\text{log }z_{2}(t)}{t}=\lambda_{2}<0\;a.s.\;$$

*for any initial condition* $u_{2}\in D_{2}^{\circ }$.

**C4**. If *z*_1_(0) > 0 and *z*_2_(0) > 0 then we get *z*_1_(*t*) > 0 for all *t* ≥ 0 a.s. and *z*_2_(*t*) > 0 for all *t* ≥ 0 a.s. due to the last two equations of (3). Again, from these last two equations, using Ito’s formula gives

$$d\left(\text{log }z_{1}\right)=\left(e_{1}y-d_{1}-\frac{\tau_{1}^{2}}{2}\right)dt+\tau_{1}dW_{1},$$

$$d\left(\text{log }z_{2}\right)=\left(e_{2}y-d_{2}-\frac{\tau_{2}^{2}}{2}\right)dt+\tau_{2}dW_{2}.$$

These equations follow that

$$\frac{e_{1}}{e_{2}}d\left(\text{log }z_{2}\right)=d\left(\text{log }z_{1}\right)+e_{1}\left(\frac{d_{1}}{e_{1}}+\frac{\tau_{1}^{2}}{2e_{1}}-\frac{d_{2}}{e_{2}}-\frac{\tau_{2}^{2}}{2e_{2}}\right)+d\left(\frac{e_{1}}{e_{2}}\tau_{2}W_{2}-\tau_{1}W_{1}\right).$$

Integrate both sides from 0 to *t*, we get

$$\text{log }\left(\frac{z_{2}{\left(t\right)}^{\frac{e_{1}}{e_{2}}}}{z_{1}(t)}\right)=\text{log }\left(\frac{z_{2}{\left(0\right)}^{\frac{e_{1}}{e_{2}}}}{z_{1}(0)}\right)+\left[e_{1}\left(\frac{d_{1}}{e_{1}}+\frac{\tau_{1}^{2}}{2e_{1}}-\frac{d_{2}}{e_{2}}-\frac{\tau_{2}^{2}}{2e_{2}}\right)+\frac{e_{1}\tau_{2}}{e_{2}}\frac{W_{2}(t)}{t}-\tau_{1}\frac{W_{1}(t)}{t}\right]t.$$

Thus for a.s.

$$z_{2}{\left(t\right)}^{\frac{e_{1}}{e_{2}}}=Cz_{1}(t)\text{ exp }\left\{\left[e_{1}\left(\frac{d_{1}}{e_{1}}+\frac{\tau_{1}^{2}}{2e_{1}}-\frac{d_{2}}{e_{2}}-\frac{\tau_{2}^{2}}{2e_{2}}\right)+\frac{e_{1}\tau_{2}}{e_{2}}\frac{W_{2}(t)}{t}-\tau_{1}\frac{W_{1}(t)}{t}\right]t\right\}$$

where $C≔\text{log }\left(\frac{z_{2}{\left(0\right)}^{\frac{e_{1}}{e_{2}}}}{z_{1}(0)}\right)$. Consider 3 cases.

**C41.** If $\frac{d_{1}}{e_{1}}+\frac{\tau_{1}^{2}}{2e_{1}}<\frac{d_{2}}{e_{2}}+\frac{\tau_{2}^{2}}{2e_{2}}$, that is *h*_1_ < *h*_2_, then, by the same arguments as in the ODE analysis (see [23]), we can easily show that *z*_2_(*t*) → 0 a.s. as *t* → ∞. Hence the long-term behavior of the system (3) is reduced to that of the system (4) in which the complete dynamics of the system (4) is obtained in C2.

**C42**. If *h*_1_ > *h*_2_ then, by the same reasons as in the ODE analysis (see [23]), it can be shown that *z*_1_(*t*) → 0 a.s. as *t* → ∞. Hence the long-term behavior of the system (3) is reduced to that of the system (5) in which the complete dynamics of the system (5) is obtained in C3.

**C43**. Assume that *h*_1_ = *h*_2_. Then, let $\rho =\frac{e_{2}}{e_{1}}$, for *k* ∈ [0, ∞] we get

(8) $$z_{2}(t)=kz_{1}{\left(t\right)}^{\rho }\text{ exp}\left\{\tau_{2}W_{2}(t)-\rho \tau_{1}W_{1}(t)\right\}\;a.s.$$

Notice that, when *k* = 0, *z*_2_(*t*) ≡ 0 a.s. So the system (3) is reduced to the system (4). When *k* = ∞, *z*_1_(*t*) ≡ 0 a.s. Thus the system (3) is reduced to the system (5). Now we suppose that 0 < *k* < ∞. Then the long-term behavior of the system (3) is determined by the parameter *a* and the combined parameter $\lambda ≔y_{1}^{*}-h$ where *h* ≔ *h*_1_ = *h*_2_. From the boundary analysis of the system (3) in A, B, C1, C2, and C3, the system (3) has 5 ergodic invariant probability measures on the boundary *∂D* of *D* which are

$\mu_{0}=\delta_{0}^{*}\times \delta_{0}^{*}\times \delta_{0}^{*}\times \delta_{0}^{*}$ on {*x* = 0} with supp(*μ*_0_) = {(0, 0, 0, 0)},

$\mu_{1}=\delta_{1}^{*}\times \delta_{0}^{*}\times \delta_{0}^{*}\times \delta_{0}^{*}$ on {*y* = 0} with supp(*μ*_1_) = {(1, 0, 0, 0)},

$\mu_{2}=\delta_{x_{1}^{*}}^{*}\times \delta_{y_{1}^{*}}^{*}\times \delta_{0}^{*}\times \delta_{0}^{*}$ on {*z*_1_ = 0, *z*_2_ = 0} with $\text{supp}\left(\mu_{2}\right)=\left\{\left(x_{1}^{*},y_{1}^{*},0,0\right)\right\}$,

*μ*_3_ on {*z*_2_ = 0} with

$$\text{supp}\left(\mu_{3}\right)=\left\{\left(\frac{l_{1}z_{1}+1}{a},\frac{r(a-1)}{a(a+r)}-\frac{rl_{1}z_{1}}{a(a+r)},z_{1},0\right):z_{1}\in \left(0,\frac{a-1}{l_{1}}\right)\right\},$$

*μ*_4_ on {*z*_1_ = 0} with

$$\text{supp}\left(\mu_{4}\right)=\left\{\left(\frac{1}{a},\frac{r(a-1)}{a(a+r)}-\frac{l_{2}z_{2}}{a+r},0,z_{2}\right):z_{2}\in \left(0,\frac{r(a-1)}{al_{2}}\right)\right\}.$$

Note that the invariant probability measure ${\bar{\mu }}_{3}$ of the system (4) in $D_{1}^{\circ }$ is the projection of *μ*_3_ onto {*z*_2_ = 0} and the invariant probability measure ${\tilde{\mu }}_{4}$ of the system (5) in $D_{2}^{\circ }$ is the projection of *μ*_4_ onto {*z*_1_ = 0}. To study the long-term behavior of the system (3) starting in the interior *D*° of *D*, we compute the Lyapunov exponents of the ergodic invariant probability measures *μ*_0_, *μ*_1_, and *μ*_2_ as we did in C2 and C3. Since *λ*_1_(*μ*_0_) = *r* > 0, *μ*_0_ is always a repeller. If 0 < *a* < 1, then the same arguments as in C2 and C3 imply *μ*_1_ is global attractor. If *a* > 1, then the dynamics of the system (3) is determined by the combined parameter *λ*, which is summarized in the following two theorems whose proofs will be given in Subsection 3.3.

**Theorem 3.5**. *Suppose that h* ≔ *h*_1_ = *h*_2_. *Let U*^*u*^(*t*) *be the solution to the system* (3) *with initial condition u in D*°. *Assume that a* > 1 *and λ* > 0. *Then*

- *There exists a collection of invariant probability measures* {*π*(*k*)}_*k*∈(0,∞)_ *where each π*(*k*) *is supported by*
$$S(k)≔\left\{\left(\frac{l_{1}z_{1}+1}{a},\frac{r(a-1)}{a(a+r)}-\frac{rl_{1}z_{1}}{a(a+r)}-\frac{l_{2}kz_{1}^{\rho }}{a+r},z_{1},kz_{1}^{\rho }\right):z_{1}\in \left(0,\frac{a-1}{l_{1}}\right)\right\}.$$
- *For each k* ∈ (0, ∞), *the transition probability P*(*t*, *u*, ·) *of the solution U*^*u*^(*t*) *starting in* $\left\{z_{2}=kz_{1}^{\rho }\right\}$ *converges to π*(*k*) *exponentially fast in total variation norm*. *In other words*, *for each k* ∈ (0, ∞), *when starting in* $\left\{z_{2}=kz_{1}^{\rho }\right\}$, *the solution U*^*u*^(*t*) *is exponentially ergodic with respect to π*(*k*).
- *Moreover*, *for all π*(*k*)-*integrable function f and for all u* ∈ *D*° *we get*
$$\underset{t\to \infty }{\text{lim}}\frac{1}{t}\int_{0}^{t}f\left(U^{u}(s)\right)ds=\int_{D^{\circ }}f(u)\pi (k)(du)\;a.s.$$
  *This is called the strong law of large number for each π*(*k*).

**Theorem 3.6**. *Suppose that h* ≔ *h*_1_ = *h*_2_. *Let U*^*u*^(*t*) *be the solution to the system* (3) *with initial condition u in D*°. *Assume that a* > 1 *and λ* < 0. *Then* $U^{u}(t)\to \left(x_{1}^{*},y_{1}^{*},0,0\right)$ *a.s*. *as t* → ∞. *Furthermore*,

$$\underset{t\to \infty }{\text{lim}}\frac{\text{log }z_{1}(t)}{t}=e_{1}\lambda <0\;a.s.\;and\;\underset{t\to \infty }{\text{lim}}\frac{\text{log }z_{2}(t)}{t}=e_{2}\lambda <0\;a.s.$$

*for any initial condition u* ∈ *D*°.

### 3.1. Proof of Theorem 2.1

To complete the proof of Theorem 2.1, we give the detailed proofs of Theorem 3.1 and Theorem 3.2 in this subsection. Notice that we always assume *a* > 1.

First of all, since the noises of the system (4) are degenerate, we need to show the hypoellipticity (see Appendix A.1) of the solution *U*_1_(*t*) to the system (4), which makes sure that any positive solution state can move close to any other positive solution state in a finite time. In other words, there are sufficient noises in the system (4) that can locally push its dynamics in all directions. Indeed, we rewrite the system (4) in the Stratonovich form

(9) $$dx=[rx(1-x-y)-axy]dt,\;dy=\left(axy-l_{1}yz_{1}-y\right)dt,\;dz_{1}=\left(e_{1}y-d_{1}-\frac{\tau_{1}^{2}}{2}\right)z_{1}dt+\tau_{1}z_{1}\circ dW_{1}.$$

Let

$$A=\left(\begin{array}{c} A_{1}\left(u_{1}\right)\\ A_{2}\left(u_{1}\right)\\ A_{3}\left(u_{1}\right) \end{array}\right)=\left(\begin{array}{c} rx(1-x-y)-axy\\ axy-l_{1}yz_{1}-y\\ e_{1}yz_{1}-d_{1}z_{1}-\frac{1}{2}\tau_{1}^{2}z_{1} \end{array}\right)\text{ and }B=\left(\begin{array}{l} B_{1}\left(u_{1}\right)\\ B_{2}\left(u_{1}\right)\\ B_{3}\left(u_{1}\right) \end{array}\right)=\left(\begin{array}{c} 0\\ 0\\ \tau_{1}z_{1} \end{array}\right).$$

From Appendix A.1, the solutions *U*_1_(*t*) to the system (4) are said to satisfy Hörmander’s condition if the set of vector fields *B*, [*A*, *B*], [*A*, [*A*, *B*]], [*B*, [*A*, *B*]], ⋯ spans ${\mathbb{R}}^{3}$ at every point *u*_1_ = (*x*, *y*, *z*_1_) ∈ $D_{1}^{\circ }$ where [·, ·] is the Lie Bracket defined by [*A*, *B*] = ([*A*, *B*]_1_, [*A*, *B*]_2_, [*A*, *B*]_3_)^*T*^ where, for *j* = 1, 2, 3,

$${\left[A,B\right]}_{j}≔\left(A_{1}\frac{\partial B_{j}}{\partial x}-B_{1}\frac{\partial A_{j}}{\partial x}\right)+\left(A_{2}\frac{\partial B_{j}}{\partial y}-B_{2}\frac{\partial A_{j}}{\partial y}\right)+\left(A_{3}\frac{\partial B_{j}}{\partial z_{1}}-B_{3}\frac{\partial A_{j}}{\partial z_{1}}\right).$$

By computation,

$$C≔[A,B]=\left(\begin{array}{c} 0\\ \tau_{1}l_{1}yz_{1}\\ 0 \end{array}\right)\text{ and }D≔[A,[A,B]]=[A,C]=\left(\begin{array}{c} \tau_{1}l_{1}(r+a)xyz_{1}\\ \tau_{1}l_{1}yz_{1}\left(e_{1}y-d_{1}-\frac{1}{2}\tau_{1}^{2}\right)\\ -\tau_{1}l_{1}e_{1}yz_{1}^{2} \end{array}\right).$$

Clearly, the vectors *B*, *C*, and *D* span ${\mathbb{R}}^{3}$ for any $u_{1}=\left(x,y,z_{1}\right)\in D_{1}^{\circ }$. So Hörmander’s condition holds for the solutions to the system (4). Thus we proved the following lemma.

**Lemma 3.1**. *The solutions U*_1_(*t*) = (*x*(*t*), *y*(*t*), *z*_1_(*t*)) *to the system* (4) *in* $D_{1}^{\circ }$ *satisfy Hörmander’s condition*.

As a consequence of Lemma 3.1 (see Theorem 1 in Appendix A.1), the transition probability *P*(*t*, *u*_10_, ·) of the solutions *U*_1_(*t*) has density *p*(*t*, *u*_10_, *u*_1_) which is smooth in $\left(u_{10},u_{1}\right)\in D_{1}^{\circ }\times D_{1}^{\circ }$.

Next, we consider the control system corresponding to the system (9)

(10) $${\dot{x}}_{\phi }=rx_{\phi }\left(1-x_{\phi }-y_{\phi }\right)-ax_{\phi }y_{\phi },\;{\dot{y}}_{\phi }=ax_{\phi }y_{\phi }-l_{1}y_{\phi }z_{1\phi }-y_{\phi },\;{\dot{z}}_{1\phi }=\left(e_{1}y_{\phi }-d_{1}-\frac{\tau_{1}^{2}}{2}+\tau_{1}\phi \right)z_{1\phi },$$

where *ϕ* = *ϕ*(*t*) is from the set of piecewise continuous real-valued functions defined on ${\mathbb{R}}_{+}$. Let (*x*_*ϕ*_(*t*, *u*_1_), *y*_*ϕ*_(*t*, *u*_1_), *z*_1*ϕ*_(*t*, *u*_1_)) be the solution to the system (10) with control *ϕ* and initial value $u_{1}=\left(x,y,z_{1}\right)\in D_{1}^{\circ }$.

To study the ergodic properties of the process $U_{1}^{u_{1}}(t)$, we utilize the ideas in geometric control theory (see [13]) to investigate reachable sets of the control system (10). Roughly speaking, starting with initial point *u*_10_ = (*x*_0_, *y*_0_, *z*_10_) in $D_{1}^{\circ }$, the collection of all points

$$u_{1}=\left(x_{1},y_{1},z_{11}\right)=\left(x_{\phi }\left(t,u_{10}\right),y_{\phi }\left(t,u_{10}\right),z_{1\phi }\left(t,u_{10}\right)\right)$$

under all piecewise continuous controls *ϕ*(·), where time *t* is fixed, forms a reachable set of *u*_10_. In view of the support theorem (see Theorem 2 in Appendix A.2), we can obtain the desired properties of the transition probability *P*(*t*, *u*_10_, ·) and invariant probability measures of the system (4) by looking into the reachable sets of different initial values. For convenience, we let

$$f_{1}\left(x_{\phi },y_{\phi },z_{1\phi }\right)≔rx_{\phi }\left(1-x_{\phi }-y_{\phi }\right)-ax_{\phi }y_{\phi },$$

$$f_{2}\left(x_{\phi },y_{\phi },z_{1\phi }\right)≔ax_{\phi }y_{\phi }-l_{1}y_{\phi }z_{1\phi }-y_{\phi },$$

$$f_{3}\left(x_{\phi },y_{\phi },z_{1\phi }\right)≔\left(e_{1}y_{\phi }-d_{1}-\frac{\tau_{1}^{2}}{2}\right)z_{1\phi },$$

then the system (10) is equivalent to

$${\dot{x}}_{\phi }=f_{1}\left(x_{\phi },y_{\phi },z_{1\phi }\right),$$

$${\dot{y}}_{\phi }=f_{2}\left(x_{\phi },y_{\phi },z_{1\phi }\right),$$

$${\dot{z}}_{1\phi }=f_{3}\left(x_{\phi },y_{\phi },z_{1\phi }\right)+\tau_{1}\phi z_{1\phi }.$$

The results of the dynamics of the system (10) are given in the following claims.

**Claim 3.1**. *Let $\left(x_{0},y_{0},z_{10}\right)\in D_{1}^{\circ }$ and z*_11_ ∈ (0, ∞). *Then*, *for any ϵ* > 0, *there are a control ϕ*(·) *and a time T* > 0 *such that*

$$\left|x_{\phi }\left(T,x_{0},y_{0},z_{10}\right)-x_{0}\right|<\epsilon ,$$

$$\left|y_{\phi }\left(T,x_{0},y_{0},z_{10}\right)-y_{0}\right|<\epsilon ,\;and$$

$$z_{1\phi }\left(T,x_{0},y_{0},z_{10}\right)=z_{11}.$$

**Remark**. Claim 3.1 indicates that we can control the solution of the system (10) to move back and forth along the positive *z*_1_-direction while the other directions of the solution still remain within a small neighborhood of their initial values.

**Proof of Claim 3.1**. Let *u*_10_ ≔ (*x*_0_, *y*_0_, *z*_10_) and suppose *z*_10_ < *z*_11_. Let

$$\rho_{1}=\text{sup}\left\{\left|f_{1}\left(x,y,z_{1}\right)\right|,\left|f_{2}\left(x,y,z_{1}\right)\right|,\left|f_{3}\left(x,y,z_{1}\right)\right|:\left|x-x_{0}\right|\leq \epsilon ,\left|y-y_{0}\right|\leq \epsilon ,z_{10}\leq z\leq z_{11}\right\}.$$

We choose *ϕ*(*t*) ≡ *ρ*_2_ such that $0<z_{11}-z_{10}<\epsilon \left(\frac{\tau_{1}\rho_{2}z_{10}}{\rho_{1}}-1\right)$. This implies that *τ*_1_*ρ*_2_*z*_10_ −*ρ*_1_ > 0 and hence *ż*_1*ϕ*_(0, *u*_10_) = *f*_3_(*u*_10_) + *τ*_1_*ρ*_2_*z*_10_ ≥ *τ*_1_*ρ*_2_*z*_10_ − *ρ*_1_ > 0. After time 0, *z*_1*ϕ*_ is increasing from *z*_10_. Now suppose that there were the first time $t\in \left(0,\frac{\epsilon }{\rho_{1}}\right)$ so that $\left|x_{\phi }\left(t,u_{10}\right)-x_{0}\right|>\epsilon$. Then, by mean value theorem, we would have

$$\epsilon <\left|x_{\phi }\left(t,u_{10}\right)-x_{0}\right|=\left|{\dot{x}}_{\phi }\left(\eta ,u_{10}\right)\right|t=\left|f_{1}\left(U_{1}^{u_{10}}(\eta )\right)\right|t\leq \rho_{1}\cdot \frac{\epsilon }{\rho_{1}}=\epsilon ,$$

for some *η* ∈ (0, *t*), which is a contradiction. Thus |*x*_*ϕ*_(*t*, *u*_10_) − *x*_0_| ≤ *ϵ* for all $t\in \left(0,\frac{\epsilon }{\rho_{1}}\right)$ By similar arguments, |*y*_*ϕ*_(*t*, *u*_10_) − *y*_0_| ≤ *ϵ* for all $t\in \left(0,\frac{\epsilon }{\rho_{1}}\right)$. Next, if for all $t\in \left(0,\frac{\epsilon }{\rho_{1}}\right)$ we had *z*_1*ϕ*_(*t*, *u*_10_) < *z*_11_ then it would imply that $z_{1\phi }\left(\frac{\epsilon }{\rho_{1}},u_{10}\right)={\text{lim}}_{t\to \frac{\epsilon }{\rho_{1}}}z_{1\phi }\left(t,u_{10}\right)\leq z_{11}$. But then, by mean value theorem,

$$\frac{\epsilon }{\rho_{1}}\left(\tau_{1}\rho_{2}z_{10}-\rho_{1}\right)>z_{11}-z_{10}\geq z_{1\phi }\left(\frac{\epsilon }{\rho_{1}},u_{10}\right)-z_{1\phi }\left(0,u_{10}\right)\;={\dot{z}}_{1\phi }\left(\bar{\eta },u_{10}\right)\frac{\epsilon }{\rho_{1}}\geq \left(\tau_{1}\rho_{2}z_{10}-\rho_{1}\right)\frac{\epsilon }{\rho_{1}}$$

for some $\bar{\eta }\in \left(0,\frac{\epsilon }{\rho_{1}}\right)$, which is a contradiction. Therefore, there is a time $T\in \left(0,\frac{\epsilon }{\rho_{1}}\right)$ such that *z*_1*ϕ*_(*T*, *u*_10_) = *z*_11_. In the case *z*_10_ > *z*_11_, the control *ϕ*(·) is constructed similarly. So the proof is completed. □

**Claim 3.2.** *Let* (*x*_0_, *y*_0_) ∈ Δ° = {(*x*, *y*): *x* > 0, *y* > 0, *x* + *y* < 1} *and z*_10_ ∈ (0, ∞). *Suppose a* > 1 *such that* $\left(x^{*},y^{*}\right)≔\left(\frac{l_{1}z_{10}+1}{a},\frac{r\left(a-1-l_{1}z_{10}\right)}{a(a+r)}\right)\in \Delta^{\circ }$ *and let* $z^{*}=\frac{a-1}{l_{1}}$. *Then we have the following*

- *If z*_10_ ∈ (0, z*) *then for all ϵ* > 0 *there are a control ϕ*(·) *and a time T* > 0 *such that*
$$\left|x_{\phi }\left(T,x_{0},y_{0},z_{10}\right)-x^{*}\right|<\epsilon ,$$
$$\left|y_{\phi }\left(T,x_{0},y_{0},z_{10}\right)-y^{*}\right|<\epsilon ,\;and$$
$$z_{1\phi }\left(t,x_{0},y_{0},z_{10}\right)=z_{10}\forall t\in [0,T].$$
- *If z*_10_ ≥ z* *then for all ϵ* > 0 *there exist a control ϕ*(·) *and a time T* > 0 *such that*
$$1-x_{\phi }\left(T,x_{0},y_{0},z_{10}\right)<\epsilon ,$$
$$y_{\phi }\left(T,x_{0},y_{0},z_{10}\right)<\epsilon ,\;and$$
$$z_{1\phi }\left(t,x_{0},y_{0},z_{10}\right)=z_{10}\forall t\in [0,T].$$

**Remark**. Claim 3.2 shows that if we hold the *z*-direction of the solution of the system (10) then both the *x*- and *y*- directions will end up within a small neighborhood of a fixed point after a finite time. In other words, when the solution of the system (10) starts in $D_{1}^{\circ }$, it will concentrate around a line segment in *D*_1_ as time goes by. This claim helps us to describe exactly the support *S*_1_ of the invariant probability measure ${\bar{\mu }}_{3}$ of the system (4) in $D_{1}^{\circ }$.

**Proof of Claim 3.2**. Let $u_{10}=\left(x_{0},y_{0},z_{10}\right)\in D_{1}^{\circ }$ where *z*_10_ ∈ (0, ∞). Consider the ODE system

(11) $$\dot{x}=rx(1-x-y)-axy,\;\dot{y}=axy-l_{1}yz_{1}-y,\;{\dot{z}}_{1}=0,$$

with initial condition *u*_10_. The last equation of (11) implies *z*_1_(*t*) ≡ *z*_10_. So the system (11) is reduced to 2-dim ODE system

(12) $$\dot{x}=rx(1-x-y)-axy,\;\dot{y}=axy-l_{1}yz_{10}-y,$$

with initial condition (*x*_0_, *y*_0_) ∈ Δ°. Consider 2 cases:

- If *z*_10_ ∈ (0, z*) then it is clear that (x*, y*) ∈ Δ° is the unique positive equilibrium point of (12). Using the Lyapunov function
$$V_{2}(x,y)=x-x^{*}-x^{*}\text{ log }\frac{x}{x^{*}}+\frac{r+a}{r}\left(y-y^{*}-y^{*}\text{ log }\frac{y}{y^{*}}\right)$$
  and Lasalle’s principle, we can prove that (x*, y*) is globally asymptotically stable. Let $\left(\bar{x}(t),\bar{y}(t),{\bar{z}}_{1}(t)\right)$ be the solution to (11) with initial condition *u*_10_ ∈ Δ° × (0, z*). Then ${\bar{z}}_{1}(t)\equiv z_{10}$ and $(\bar{x}(t),\bar{y}(t))\to \left(x^{*},y^{*}\right)$ as *t* → ∞. With the feedback control *ϕ*_1_(·) satisfying $e_{1}\bar{y}(t)-d_{1}-\frac{\tau_{1}^{2}}{2}+\tau_{1}\phi_{1}(t)\equiv 0$, we have
$$(x_{\phi_{1}}(t),(y_{\phi_{1}}(t),\left(z_{1\phi_{1}}(t)\right)=\left(\bar{x}(t),\bar{y}(t),{\bar{z}}_{1}(t)\right)\;\forall t\geq 0$$
  where $\left(x_{\phi_{1}}(t),(y_{\phi_{1}}(t),(z_{1\phi_{1}}(t)\right)$ is the solution to (10) with the control *ϕ*_1_(·) above and initial condition *u*_10_ ∈ Δ° × (0, z*). Therefore the result follows.
- If *z*_10_ ≥ z* then, from the second equation of (11), $\dot{y}=axy-l_{1}yz_{10}-y\leq axy-ay$. Let $\left(\tilde{x}(t),\tilde{y}(t)\right)$ be the solution to
(13) $$\dot{x}=rx(1-x-y)-axy,\;\dot{y}=axy-ay,$$
  with initial condition $(\tilde{x}(0),\tilde{y}(0))=\left(x_{0},y_{0}\right)\in \Delta^{\circ }$. Use the Lyapunov function $V_{3}(x,y)=x-1-\text{log }x+\frac{r+a}{a}y$ and Lasalle’s principle, can show that (1, 0) is globally asymptotically stable equilibrium of (13). It follows that $\left(\tilde{x}(t),\tilde{y}(t))\to (1,0\right)$ as *t* → ∞. Let $\left(\underline{x}(t),\underline{y}(t),{\underline{z}}_{1}(t)\right)$ be the solution to (11) with initial condition *u*_10_ ∈ Δ° ×[z*, ∞). Clearly, ${\underline{z}}_{1}(t)\equiv z_{10}$. By comparison theorem for ODEs, since $1>\underline{x}(t)\geq \tilde{x}(t)$ and $0<\underline{y}(t)\leq \tilde{y}(t)$, letting *t* → ∞ yields $\;(\underline{x}(t),\underline{y}(t))\to (1,0)$. Then the result follows by choosing the feedback control *ϕ*_2_(·) satisfying $e_{1}\underline{y}(t)-d_{1}-\tau_{1}^{2}/2+\tau_{1}\phi_{2}(t)\equiv 0$. □

**Claim 3.3**. *For any* $u_{1}=\left(x,y,z_{1}\right)\in D_{1}^{\circ }$, *we can find a point* $\left(x_{2}^{*},y_{2}^{*},z_{1}^{*}\right)\in D_{1}^{\circ }$ *with the following properties: if* $0<\delta <\text{min}\left\{x_{2}^{*},y_{2}^{*},\frac{1}{\sqrt{2}}\left(x_{2}^{*}+y_{2}^{*}\right),z_{1}^{*}\right\}$ *and let*

$$V_{\delta }≔\left(x_{2}^{*}-\delta ,x_{2}^{*}+\delta \right)\times \left(y_{2}^{*}-\delta ,y_{2}^{*}+\delta \right)\times \left(z_{1}^{*}-\delta ,z_{1}^{*}+\delta \right),\;\text{then }$$

- *there are a control ϕ*(·) *and a time T* > 0 *so that* (*x*_*ϕ*_(*T*, *u*_1_), *y*_*ϕ*_(*T*, *u*_1_), *z*_1*ϕ*_(*T*, *u*_1_)) ∈ *V*_*δ*_,
- *there exist a neighborhood S*_*δ*_ ⊂ *V*_*δ*_ *and a control ϕ*(·) *such that S*_*δ*_ *is invariant under* (10), *that is*, *for all t* ≥ 0 *and u*_1_ ∈ *S*_*δ*_, (*x*_*ϕ*_(*t*, *u*_1_), *y*_*ϕ*_(*t*, *u*_1_), *z*_1*ϕ*_(*t*, *u*_1_)) ∈ *S*_*δ*_.

**Claim 3.4.** *For any* $u_{1}=\left(x,y,z_{1}\right)\in D_{1}^{\circ }\;$ *and for any* $0<\delta <\text{min}\left\{x_{1}^{*},y_{1}^{*},\frac{1}{\sqrt{2}}\left(x_{1}^{*}+y_{1}^{*}\right)\right\}$, *there are a control ϕ*(·) *and a time T* > 0 *such that*

$$\left(x_{\phi }\left(T,u_{1}\right),y_{\phi }\left(T,u_{1}\right),z_{1\phi }\left(T,u_{1}\right)\right)\in W_{\delta }≔\left(x_{1}^{*}-\delta ,x_{1}^{*}+\delta \right)\times \left(y_{1}^{*}-\delta ,y_{1}^{*}+\delta \right)\times (0,\delta ).$$

**Remark**. Claim 3.3 will be used in the proofs of Lemma 3.2 (see below) and Theorem 3.1 which establish the persistence for the system (4), while Claim 3.4 will be utilized in the proof of Theorem 3.2 which establishes the extinction of the system (4). Proofs of these two claims directly follow from Claim 3.1 and Claim 3.2.

To prove Theorem 3.1, we use Theorem 3 and Theorem 4 in Appendix A.3. Theorem 4 guarantees that there exists a unique invariant probability measure ${\bar{\mu }}_{3}$ in $D_{1}^{\circ }$ for the solution *U*_1_(*t*) and, no matter where the solution *U*_1_(*t*) starts in $D_{1}^{\circ }$, once it gets into a neighborhood of the support of ${\bar{\mu }}_{3}$ it will get trapped there forever. So Theorem 4 helps prove part (i) and part (ii) of Theorem 3.1. Since the unique existence of invariant probability measure ${\bar{\mu }}_{3}$ implies the boundedness in probability on average of *U*_1_(*t*), we can apply Theorem 3 to obtain part (iii) of Theorem 3.1. Thus, to utilize Theorem 3 and Theorem 4 in Appendix A.3, we need the following lemma.

**Lemma 3.2.** *The solution process* $U_{1}^{u_{1}}(t)$ *of the system* (4) *is a T-process*. *Moreover*, *every compact set* $K\subset D_{1}^{\circ }$ *is petite for the Markov chain* (*x*(*n*), *y*(*n*), *z*_1_(*n*)), n∈ℕ.

**Proof of Lemma 3.2.** Due to Lemma 3.1, the transition probability *P*(*t*, *u*_1_, ·) of the process $U_{1}^{u_{1}}(t)$ has a smooth density function *p*(*t*, ·, ·) on $D_{1}^{\circ }\times D_{1}^{\circ }$. By standard arguments, it can be shown that the resolvent kernel

$$R\left(u_{1},A\right)=\overset{\infty }{\underset{0}{\int }}e^{-t}P\left(t,u_{1},A\right)dt$$

is continuous function in *u*_1_ for each $A\in ℬ\left(D_{1}^{\circ }\right)$. With the probability measure ^*a*^1(*dt*) = *e*^−*t*^*dt* on ${\mathbb{R}}_{+}$, *R*(*u*_1_, *A*) is its own continuous component (see Theorem 3.3 p498 in [18]). Hence $U_{1}^{u_{1}}(t)$ is a *T*-process.

Next, consider the point $\left(x_{2}^{*},y_{2}^{*},z_{1}^{*}\right)\in D_{1}^{\circ }$ as in Claim 3.3. As $D_{1}^{\circ }$ is invariant under the system (4), so $P\left(1,\left(x_{2}^{*},y_{2}^{*},z_{1}^{*}\right),D_{1}^{\circ }\right)=1$. Hence, for some $\;\left(x_{3},y_{3},z_{3}\right)\in D_{1}^{\circ }$, $p\left(1,\left(x_{2}^{*},y_{2}^{*},z_{1}^{*}\right),\left(x_{3},y_{3},z_{3}\right)\right)>0$. In view of Claim 3.3(ii) and the smoothness of the density *p*(1, ·, ·) on $D_{1}^{\circ }\times D_{1}^{\circ }$, there are a neighborhood *S*_*δ*_ of $\left(x_{2}^{*},y_{2}^{*},z_{1}^{*}\right)$ in $D_{1}^{\circ }$, which is invariant under the system (10) with some control *ϕ*(·), and an open set *G* ∋ (*x*_3_, *y*_3_, *z*_3_) in $D_{1}^{\circ }$ such that

(14) $$p\left(1,\left(x,y,z_{1}\right),\left(x^{\prime },y^{\prime },z_{1}^{\prime }\right)\right)\geq m^{\prime }>0$$

for all (*x*, *y*, *z*_1_) ∈ *S*_*δ*_ and $\left(x^{\prime },y^{\prime },z_{1}^{\prime }\right)\in G$. Now suppose *K* is any compact set in $D_{1}^{\circ }$. Then, for any *u*_1_ ∈ *K*, it follows from Claim 3.3(i) that there are a control *ϕ*(·) and a time *T* > 0 such that

$$\left(x_{\phi }\left(T,u_{1}\right),y_{\phi }\left(T,u_{1}\right),z_{1\phi }\left(T,u_{1}\right)\right)\in S_{\delta }$$

Let $n_{u_{1}}\in {\mathbb{Z}}_{+}$ such that $n_{u_{1}}>T$. By Claim 3.3(ii), we can extend the control *ϕ*(·) after time *T* so that

$$\left(x_{\phi }\left(n_{u_{1}},u_{1}\right),y_{\phi }\left(n_{u_{1}},u_{1}\right),z_{1\phi }\left(n_{u_{1}},u_{1}\right)\right)\in S_{\delta }.$$

In light of the support theorem (see Theorem 2 in Appendix A.2),

$$P\left(n_{u_{1}},u_{1},S_{\delta }\right)≕2\rho_{u_{1}}>0.$$

For each *u*_1_ ∈ *K*, since $U_{1}^{u_{1}}(t)$ is a Feller process, there exists an open set $V_{u_{1}}∍u_{1}$ such that for all $u_{1}^{\prime }\in V_{u_{1}}$ we have $P\left(n_{u_{1}},u_{1}^{\prime },S_{\delta }\right)\geq \rho_{u_{1}}$. Since *K* is compact, there is a finite number of such open sets $V_{u_{1}^{i}}(i=1,\ldots ,l)$ that satisfies $K\subset \overset{l}{\underset{i=1}{\cup }}V_{u_{1}^{i}}$. Let $\rho_{K}=\underset{i=1,\ldots ,l}{\text{min}}\rho_{u_{1}^{i}}$, then, for each *u*_1_ ∈ *K*, there is a $n_{u_{1}^{i}}\in {\mathbb{Z}}_{+}$ such that

(15) $$P\left(n_{u_{1}^{i}},u_{1},S_{\delta }\right)\geq \rho_{K}.$$

By (14) and (15), for any *u*_1_ ∈ *K* and $u_{1}^{\prime }\in G$, there exists a $n_{u_{1}^{i}}\in {\mathbb{Z}}_{+}$ such that

$$p\left(n_{u_{1}^{i}}+1,u_{1},u_{1}^{\prime }\right)=\int_{D_{1}^{\circ }}p\left(n_{u_{1}^{i}},u_{1},u_{1}^{\prime \prime }\right)p\left(1,u_{1}^{\prime \prime },u_{1}^{\prime }\right)du_{1}^{\prime \prime }\;\geq \int_{S_{\delta }}m^{\prime }p\left(n_{u_{1}^{i}},u_{1},u_{1}^{\prime \prime }\right)du_{1}^{\prime \prime }=m^{\prime }P\left(n_{u_{1}^{i}},u_{1},S_{\delta }\right),$$

which implies that

(16) $$p\left(n_{u_{1}^{i}}+1,u_{1},u_{1}^{\prime }\right)\geq m^{\prime }\rho_{K}.$$

Define the probability measure *a* on ℕ as follows

$$a(n)=\{\begin{array}{ll} \frac{1}{l} & \text{if }n=n_{u_{1}^{i}}+1(i=1,\ldots ,l),\\ 0 & \text{otherwise,} \end{array}$$

and define the kernel, for *u*_1_ ∈ *K* and $Q\in ℬ\left(D_{1}^{\circ }\right)$,

$$K_{a}\left(u_{1},Q\right)=\overset{\infty }{\underset{n=0}{\sum }}P\left(n,u_{1},Q\right)a(n)=\frac{1}{l}\overset{l}{\underset{i=0}{\sum }}P\left(n_{u_{1}^{i}}+1,u_{1},Q\right).$$

Then it follows from (16) that, for all $Q\in ℬ\left(D_{1}^{\circ }\right)$,

$$K_{a}\left(u_{1},Q\right)=\frac{1}{l}\overset{l}{\underset{i=0}{\sum }}\int_{Q}p\left(n_{u_{1}^{i}}+1,u_{1},u_{1}^{\prime }\right)du_{1}^{\prime }\geq \frac{1}{l}\overset{l}{\underset{i=0}{\sum }}\int_{G\cap Q}p\left(n_{u_{1}^{i}}+1,u_{1},u_{1}^{\prime }\right)du_{1}^{\prime }\geq \rho_{K}m^{\prime }\mu (G\cap Q)$$

where *μ* is the Lebesgue measure on $ℬ\left(D_{1}^{\circ }\right)$. Let *ψ*(*Q*) ≔ *ρ*_*K*_*m*′*μ*(*G* ∩*Q*) for $Q\in ℬ\left(D_{1}^{\circ }\right)$ then it is clear that *ψ* is a nontrivial measure on $D_{1}^{\circ }$ and *K*_*a*_(*u*_1_, *Q*) ≥ *ψ*(*Q*) for all *u*_1_ ∈ *K* and for all $Q\in ℬ\left(D_{1}^{\circ }\right)$. By definition, the compact set *K* is petite for the 1-skeleton Markov chain $U_{1}^{u_{1}}(n)$, n∈ℕ. This completes the proof of Lemma 3.2. □

Now we have enough preparations to give the proofs of Theorem 3.1 and Theorem 3.2.

**Proof of Theorem 3.1.** From the system (4), it is not difficult to show that $\underset{t\to \infty }{\text{lim}}\;z_{1}(t)$ exists and is finite a.s. We claim that $\underset{t\to \infty }{\text{lim}}\;z_{1}(t)>0$ a.s. Indeed, if there were an *ω* ∈ Ω such that $\underset{t\to \infty }{\text{lim}}\;z_{1}(t,\omega )=0$ then it would be easy to see that $\underset{t\to \infty }{\text{lim}}\;y(t,\omega )=y_{1}^{*}$. So, for any *ϵ* ∈ (0, *λ*_1_), there exists a *T* > 0 so that *t* ≥ *T* implies $y(t,\omega )-y_{1}^{*}>-\frac{\epsilon }{e_{1}}$. But then, for all *t* ≥ *T*,

$$z_{1}(t,\omega )=z_{1}(T,\omega )\text{ exp }\left\{\int_{T}^{t}e_{1}\left(y(s,\omega )-y_{1}^{*}\right)ds+\lambda_{1}(t-T)+\tau_{1}\left(W_{1}(t,\omega )-W_{1}(T,\omega )\right)\right\}\;\geq z_{1}(T,\omega )\text{ exp }\left\{\left(\lambda_{1}-\epsilon \right)(t-T)+\tau_{1}\left(W_{1}(t,\omega )-W_{1}(T,\omega )\right)\right\},$$

which follows that $\underset{t\to \infty }{\text{lim}}\;z_{1}(t,\omega )>0$. This is a contradiction. Thus there is a δ* > 0 such that

(17) $$\underset{t\to \infty }{\text{lim}}z_{1}(t)\geq \delta^{*}\text{ a}.\text{s}.\;$$

Since $\lambda_{1}=e_{1}y_{1}^{*}-d_{1}-\frac{1}{2}\tau_{1}^{2}>0$, it is clear that *e*_1_ > *d*_1_ and there is a *q* ∈ (0, 1) small enough so that $e_{1}y_{1}^{*}-d_{1}-\frac{1}{2}\tau_{1}^{2}(q+1)>0$. Now consider the system (4) in the invariant domain

$${ℳ}_{1}=\left\{\left(x,y,z_{1}\right)\in D_{1}^{\circ }:z_{1}\geq \delta^{*}\right\}.$$

Denote by ℒ the generator of the diffusion corresponding to (4). For (*x*, *y*, *z*_1_) ∈ ℳ_1_, let

$$V_{4}\left(x,y,z_{1}\right)=\frac{e_{1}}{l_{1}}y+z_{1}^{-q}+z_{1}+1,$$

since $\underset{z_{1}\to \infty }{\text{lim}}\;V_{4}\left(x,y,z_{1}\right)=\infty$, *V*_4_ is a positive norm-like function on ℳ_1_. Furthermore,

(18) $$ℒV_{4}=-qz_{1}^{-q}\left[e_{1}y_{1}^{*}-d_{1}-\frac{1}{2}\tau_{1}^{2}(q+1)\right]-d_{1}z_{1}-\frac{e_{1}}{l_{1}}y+\frac{ae_{1}}{l_{1}}xy+qz_{1}^{-q}e_{1}\left(y_{1}^{*}-y\right)\leq -\theta_{1}V_{4}+\theta_{2}$$

where $\theta_{1}≔\text{min}\left\{q\left[e_{1}y_{1}^{*}-d_{1}-\frac{1}{2}\tau_{1}^{2}(q+1)\right],d_{1},1\right\}>0$ and $\theta_{2}≔\theta_{1}+\frac{ae_{1}}{l_{1}}+q\delta^{*-q}e_{1}y_{1}^{*}<\infty$. By Theorem 4 in Appendix A.3, it follows from Lemma 3.2 and (18) that the process $U_{1}^{u_{1}}(t)$ has a unique invariant probability measure ${\bar{\mu }}_{3}$ in ℳ_1_ such that for some *H*_0_ > 0 and *γ* > 0 we get

(19) $${\left\|P\left(t,u_{1},\cdot \right)-{\bar{\mu }}_{3}(\cdot )\right\|}_{TV}\leq H_{0}\left[V_{4}\left(u_{1}\right)+1\right]e^{-\gamma t}$$

for all *t* ≥ 0 and *u*_1_ = (*x*, *y*, *z*_1_) ∈ ℳ_1_. Moreover, by the support theorem, we obtain from Claims 3.1 and 3.2 that the support of ${\bar{\mu }}_{3}$ is *S*_1_, which proves Theorem 3.1(i). To show Theorem 3.1(ii), first we can easily get the estimate

$$ℒV_{4}\left(x,y,z_{1}\right)\leq \theta_{3}V_{4}\left(x,y,z_{1}\right)$$

for all $\left(x,y,z_{1}\right)\in D_{1}^{\circ }$ and for some *θ*_3_ > 0. Then, by standard arguments, we can show that there exist *H*_1_ > 0 and *γ*_1_ > 0 such that for all *t* > 0 and $u_{1}=\left(x,y,z_{1}\right)\in D_{1}^{\circ }$

(20) $$EV_{4}\left(x\left(t,u_{1}\right),y\left(t,u_{1}\right),z_{1}\left(t,u_{1}\right)\right)\leq H_{1}V_{4}\left(u_{1}\right)e^{\gamma_{1}t}.$$

By (17), for any $u_{10}\in D_{1}^{\circ }$, there is a non-random time *t*_0_ = *t*_0_(*u*_10_) > 0 such that (*x*(*t*, *u*_10_), *y*(*t*, *u*_10_), *z*_1_(*t*, *u*_10_)) ∈ ℳ_1_for all *t* ≥ *t*_0_ a.s. Thus, from (19) and (20), we obtain the following estimate

$$P\left(t+t_{0},u_{10},\cdot \right)-{\bar{\mu }}_{3}{\left(\cdot \right)}_{TV}={\left\|\underset{{ℳ}_{1}}{\int }P\left(t_{0},u_{10},du_{1}\right)P\left(t,u_{1},\cdot \right)-\underset{{ℳ}_{1}}{\int }P\left(t_{0},u_{10},du_{1}\right){\bar{\mu }}_{3}(\cdot )\right\|}_{TV}\;\leq \underset{{ℳ}_{1}}{\int }P\left(t_{0},u_{10},du_{1}\right){\left\|P\left(t,u_{1},\cdot \right)-{\bar{\mu }}_{3}(\cdot )\right\|}_{TV}\;\leq \underset{{ℳ}_{1}}{\int }p\left(t_{0},u_{10},u_{1}\right)H_{0}\left[V_{4}\left(u_{1}\right)+1\right]e^{-\gamma t}du_{1}\;=H_{0}e^{-\gamma t}\left[\underset{{ℳ}_{1}}{\int }p\left(t_{0},u_{10},u_{1}\right)V_{4}\left(u_{1}\right)du_{1}+\underset{{ℳ}_{1}}{\int }p\left(t_{0},u_{10},u_{1}\right)du_{1}\right]\;=H_{0}e^{-\gamma t}\left[EV_{4}\left(U_{1}^{u_{10}}\left(t_{0}\right)\right)+1\right]\;\leq H_{0}\left[H_{1}V_{4}\left(u_{10}\right)e^{\gamma_{1}t_{0}}+1\right]e^{-\gamma t}\;\forall t\geq 0.$$

Then Theorem 3.1(ii) is shown. Theorem 3.1(iii) is derived from Theorem 3 in Appendix A.3 since the convergence in total variation norm implies the boundedness in probability on average. □

**Proof of Theorem 3.2**. The detailed proof is carried out in the following steps.

- Use Lyapunov function method to show $\left(x_{1}^{*},y_{1}^{*},0\right)$ is an asymptotically stable in probability equilibrium of the system (4).
- Show the process $U_{1}^{u_{1}}(t)$ is recurrent relative to some compact set $\tilde{K}$ in $D_{1}^{\circ }$. Then use Claim 3.4 and the support theorem to show that when the solution $U_{1}^{u_{1}}(t)$ starts in $\tilde{K}$ it will get into a small neighborhood of $\left(x_{1}^{*},y_{1}^{*},0\right)$ after a finite time with positive probability.
- Use the strong Markov property of $U_{1}^{u_{1}}(t)$ and the support theorem to prove that once the solution $U_{1}^{u_{1}}(t)$ enters a small neighborhood of $\left(x_{1}^{*},y_{1}^{*},0\right)$ it will get trapped there forever. So we obtain the desired result.

First, we show for any *ϵ* > 0 there is a *δ* > 0 such that

(21) $${\mathbb{P}}_{u_{1}}\left\{\underset{t\to \infty }{\text{lim}}\left(x(t),y(t),z_{1}(t)\right)=\left(x_{1}^{*},y_{1}^{*},0\right)\right\}\geq 1-\epsilon$$

for any $u_{1}=\left(x,y,z_{1}\right)\in \left(x_{1}^{*}-\delta ,x_{1}^{*}+\delta \right)\times \left(y_{1}^{*}-\delta ,y_{1}^{*}+\delta \right)\times [0,\delta )$.

Since $\lambda_{1}=e_{1}y_{1}^{*}-d_{1}-\frac{1}{2}\tau_{1}^{2}<0$, there are $0<\delta <\text{min}\left\{x_{1}^{*},y_{1}^{*},\frac{1}{\sqrt{2}}\left(x_{1}^{*}+y_{1}^{*}\right)\right\}$ and *p* ∈ (0, 1) so that $e_{1}\left(y_{1}^{*}+\delta \right)-d_{1}-\frac{1}{2}\tau_{1}^{2}(1-p)<0$. Consider the Lyapunov function $V_{5}\left(x,y,z_{1}\right)=z_{1}^{p}$, which is twice differentiable $\left(x,y,z_{1}\right)\in D_{1}^{\circ }$. Then

$$ℒV_{5}=p\left[e_{1}\left(y-y_{1}^{*}\right)+e_{1}y_{1}^{*}-d_{1}-\frac{1}{2}\tau_{1}^{2}(1-p)\right]z_{1}^{p}.$$

If *z*_1_ = 0 then *z*_1_(*t*) ≡ 0 a.s. It is straightforward to show that $(x(t),y(t))\to \left(x_{1}^{*},y_{1}^{*}\right)$ as *t* → ∞ a.s. for any (*x*(0), *y*(0)) ∈ Δ°. Thus (21) is true. Hence we only need to show (21) for (*x*, *y*, *z*_1_) ∈ *W*_*δ*_ where $W_{\delta }=\left(x_{1}^{*}-\delta ,x_{1}^{*}+\delta \right)\times \left(y_{1}^{*}-\delta ,y_{1}^{*}+\delta \right)\times (0,\delta )$. Since $y-y_{1}^{*}\leq \left|y-y_{1}^{*}\right|<\delta$, ℒ*V*_5_ ≤ *θ*_4_*V*_5_ for any *u*_1_ = (*x*, *y*, *z*_1_) ∈ *W*_*δ*_ with $\theta_{4}≔e_{1}\left(y_{1}^{*}+\delta \right)-d_{1}-\frac{1}{2}\tau_{1}^{2}(1-p)<0$. By Theorem 2.3 p112 in [17], for any *ϵ* > 0, there is a *δ* > 0 such that for any *u*_1_ ∈ *W*_*δ*_ we have ${\mathbb{P}}_{u_{1}}\left\{{\text{lim}}_{t\to \infty }z_{1}(t)=0\right\}\geq 1-\epsilon$. From the ODE analysis in [23], we can easily show that if $\underset{t\to \infty }{\text{lim}}z_{1}(t,\omega )=0\;$ for any *ω* ∈ Ω then $\underset{t\to \infty }{\text{lim}}(x(t,\omega ),y(t,\omega ))=\left(x_{1}^{*},y_{1}^{*}\right)$. Therefore (21) holds for any *u*_1_ ∈ *W*_*δ*_. So part (I) is proved.

For part (II), use Theorem 3.9 p89 in [15], we construct a nonnegative twice differentiable function *V*_6_ = *V*_6_(*x*, *y*, *z*_1_) and a compact1 set $\tilde{K}$ in $D_{1}^{\circ }$ such that ℒ*V*_6_ < 0 for all $\left(x,y,z_{1}\right)\in {\tilde{K}}^{c}$. Indeed, consider $V_{6}\left(x,y,z_{1}\right)=x+y+\frac{l_{1}}{e_{1}}z_{1}$, then $ℒV_{6}=rx(1-x-y)-y-\frac{l_{1}d_{1}}{e_{1}}z_{1}\leq r-\frac{l_{1}d_{1}}{e_{1}}z_{1}$. Let $R=\frac{re_{1}}{l_{1}d_{1}}+1$, then set $\tilde{K}≔\left\{\left(x,y,z_{1}\right)\in D_{1}^{\circ }:x+y+z_{1}\leq R\right\}$. So for $\left(x,y,z_{1}\right)\in {\tilde{K}}^{c}$ we have *x* + *y* + *z*_1_ > *R* which follows that $z_{1}>R-x-y\geq R-1=\frac{re_{1}}{l_{1}d_{1}}$. Thus ℒ*V*_6_ < 0.

Next, by Claim 3.4, for any $u_{1}=\left(x,y,z_{1}\right)\in \tilde{K}$ we can choose a control *ϕ*(·) and a time $T_{u_{1}}>0$ such that

$$\left(x_{\phi }\left(T_{u_{1}},u_{1}\right),y_{\phi }\left(T_{u_{1}},u_{1}\right),z_{1\phi }\left(T_{u_{1}},u_{1}\right)\right)\in W_{\delta }.$$

In light of the support theorem for any $u_{1}\in \tilde{K}$, there exists a $T_{u_{1}}>0$ so that

$${\mathbb{P}}_{u_{1}}\left\{\left(x\left(T_{u_{1}}\right),y\left(T_{u_{1}}\right),z_{1}\left(T_{u_{1}}\right)\right)\in W_{\delta }\right\}=2\rho^{u_{1}}>0.$$

Using the Markov-Feller property of (*x*(*t*), *y*(*t*), *z*_1_(*t*)), there exists a neighborhood $V_{u_{1}}∋u_{1}$ so that for all $u_{1}^{\prime }\in V_{u_{1}}$

$${\mathbb{P}}_{u_{1}^{\prime }}\left\{\left(x\left(T_{u_{1}}\right),y\left(T_{u_{1}}\right),z_{1}\left(T_{u_{1}}\right)\right)\in W_{\delta }\right\}>\rho^{u_{1}}.$$

Since $\tilde{K}$ is compact, there is a finite number of such neighborhoods $V_{u_{1}^{i}}(i=1,\ldots ,n)$ so that $\tilde{K}\subset \overset{n}{\underset{i=1}{\cup }}V_{u_{1}^{i}}$.

Put $T^{*}=\underset{i=1,\ldots ,n}{\text{max}}T_{u_{1}^{i}}$ and $\rho^{*}=\underset{i=1,\ldots ,n}{\text{min}}\rho^{u_{1}^{i}}$. For $u_{1}\in D_{1}^{\circ }$, set

$$\tau_{\delta }^{u_{1}}=\text{inf}\left\{t>0:U_{1}^{u_{1}}(t)\in W_{\delta }\right\}.$$

Then, for any $u_{1}\in \tilde{K}$, since the event $\tau_{\delta }^{u_{1}}<T^{*}$ is followed from the fact that there exists a $u_{1}^{i}$ such that $U_{1}^{u_{1}}\left(T_{u_{1}^{i}}\right)\in W_{\delta }$,

(22) $$\mathbb{P}\left\{\tau_{\delta }^{u_{1}}<T^{*}\right\}\geq \mathbb{P}\left\{U_{1}^{u_{1}}\left(T_{u_{1}^{i}}\right)\in W_{\delta }\right\}\geq \rho^{*}>0.$$

Since $U_{1}^{u_{1}}(t)$ is recurrent relative to $\tilde{K}$, we define a sequence of finite stopping times

$$\zeta_{0}=0,\zeta_{1}=\text{inf}\left\{t>T^{*}:U_{1}^{u_{1}}(t)\in \tilde{K}\right\},\cdots ,$$

$$\zeta_{k}=\text{inf}\left\{t>\zeta_{k-1}+T^{*}:U_{1}^{u_{1}}(t)\in \tilde{K}\right\},$$

$$\zeta_{k+1}=\text{inf}\left\{t>\zeta_{k}+T^{*}:U_{1}^{u_{1}}(t)\in \tilde{K}\right\},\cdots$$

Consider the event

$$A_{k}=\left\{U_{1}^{u_{1}}(t)\notin W_{\delta }\forall t\in \left[\zeta_{k},\zeta_{k}+T^{*}\right]\right\},k\in \mathbb{N}$$

It follows from (22) that ${\mathbb{P}}_{u_{1}}\left(A_{k}^{c}\right)=\mathbb{P}\left\{\tau_{\delta }^{{\bar{u}}_{1}}<T^{*}\right\}\geq \rho^{*}$ for all k∈ℕ where ${\bar{u}}_{1}=U_{1}^{u_{1}}\left(\zeta_{k}\right)\in \tilde{K}$. So ${\mathbb{P}}_{u_{1}}\left(A_{k}\right)\leq 1-\rho^{*}$ for all k∈ℕ. Using the strong Markov property of $U_{1}^{u_{1}}(t)$, we get

$${\mathbb{P}}_{u_{1}}\left(A_{1}\cap A_{2}\right)={\mathbb{P}}_{u_{1}}\left(A_{1}\right){\mathbb{P}}_{U_{1}^{u_{1}}\left(\zeta_{2}\right)}\left(A_{2}\right)\leq {\left(1-\rho^{*}\right)}^{2}$$

and, by induction, we obtain

$${\mathbb{P}}_{u_{1}}\left(\overset{n}{\underset{k=1}{\cap }}A_{k}\right)\leq {\left(1-\rho^{*}\right)}^{n}\to 0\text{ as }n\to \infty .$$

As a result, ${\mathbb{P}}_{u_{1}}\left(\overset{\infty }{\underset{k=1}{\cup }}A_{k}\right)=0$. In other words,

(23) $${\mathbb{P}}_{u_{1}}\left(\tau_{\delta }^{u_{1}}<\infty \right)=1.$$

Again, by the strong Markov property of $U_{1}^{u_{1}}(t)$, (21) and (23) imply that, for any $u_{1}\in D_{1}^{\circ }$,

$$\mathbb{P}\left\{\underset{t\to \infty }{\text{lim}}U_{1}^{u_{1}}(t)=\left(x_{1}^{*},y_{1}^{*},0\right)\right\}\geq 1-\epsilon .$$

Letting *ϵ* → 0 gives

$$\mathbb{P}\left\{\underset{t\to \infty }{\text{lim}}U_{1}^{u_{1}}(t)=\left(x_{1}^{*},y_{1}^{*},0\right)\right\}=1\text{ for all }u_{1}\in D_{1}^{\circ }.$$

Moreover, by the last equation of (4),

$$\frac{\text{log }z_{1}(t)}{t}=\frac{\text{log }z_{1}}{t}+\frac{1}{t}\overset{t}{\underset{0}{\int }}\left(e_{1}y(s)-d_{1}-\frac{1}{2}\tau_{1}^{2}\right)ds+\tau_{1}\frac{W_{1}(t)}{t}.$$

Thus

$$\underset{t\to \infty }{\text{lim}}\frac{\text{log }z_{1}(t)}{t}=\lambda_{1}<0\text{ a}.\text{s}.\;$$

This completes the proof. □

### 3.2. Proof of Theorem 2.2

Proof of Theorem 2.2 is similar to proving Theorem 2.1. Because there are several modifications needed, we will state without proofs all lemmas and claims that are necessary for the proofs of Theorem 3.3 and Theorem 3.4. Finally, we only sketch the main points of these two theorems’ proofs and the details are left to reader. Notice that we always suppose that *a* > 1.

First, we rewrite the system (5) in the Stratonovich form

(24) $$dx=\left[rx(1-x-y)-axy-l_{2}xz_{2}\right]dt,\;dy=(axy-y)dt,\;dz_{2}=\left(e_{2}y-d_{2}-\frac{\tau_{2}^{2}}{2}\right)z_{2}dt+\tau_{2}z_{2}\circ dW_{2}.$$

Let

$$\bar{A}=\left(\begin{array}{c} {\bar{A}}_{1}\left(u_{2}\right)\\ {\bar{A}}_{2}\left(u_{2}\right)\\ {\bar{A}}_{3}\left(u_{2}\right) \end{array}\right)=\left(\begin{array}{c} rx(1-x-y)-axy-l_{2}xz_{2}\\ axy-y\\ e_{2}yz_{2}-d_{2}z_{2}-\frac{1}{2}\tau_{2}^{2}z_{2} \end{array}\right)\text{ and }\bar{B}=\left(\begin{array}{c} {\bar{B}}_{1}\left(u_{2}\right)\\ {\bar{B}}_{2}\left(u_{2}\right)\\ {\bar{B}}_{3}\left(u_{2}\right) \end{array}\right)=\left(\begin{array}{c} 0\\ 0\\ \tau_{2}z_{2} \end{array}\right).$$

By computation, we can check that $\bar{B}$, $\left[\bar{A},\bar{B}\right]$ and $\left[\bar{A},[\bar{A},\bar{B}]\right]$ span ${\mathbb{R}}^{3}$ for every point $u_{2}\in D_{2}^{\circ }$. Hence we get the following lemma.

**Lemma 3.3.** *The solution* $U_{2}^{u_{2}}(t)$ *to the system* (5) *in* $D_{2}^{\circ }$ *satisfies Hörmander’s condition*. *Therefore its transition probability P*(*t*, *u*_20_, ·) *has density p*(*t*, *u*_20_, *u*_2_) *which is smooth in* $\left(u_{20},u_{2}\right)\in D_{2}^{\circ }\times D_{2}^{\circ }$.

Next we consider the control system corresponding to the system (24)

(25) $${\dot{x}}_{\phi }=rx_{\phi }\left(1-x_{\phi }-y_{\phi }\right)-ax_{\phi }y_{\phi }-l_{2}x_{\phi }z_{2\phi },\;{\dot{y}}_{\phi }=ax_{\phi }y_{\phi }-y_{\phi },\;{\dot{z}}_{2\phi }=\left(e_{2}y_{\phi }-d_{2}-\frac{\tau_{2}^{2}}{2}+\tau_{2}\phi \right)z_{2\phi },$$

where *ϕ* = *ϕ*(*t*) is from the set of piecewise continuous real-valued functions defined on ${\mathbb{R}}_{+}$. Let (*x*_*ϕ*_(*t*, *u*_2_), *y*_*ϕ*_(*t*, *u*_2_), *z*_2*ϕ*_(*t*, *u*_2_)) be the solution to the system (25) with control *ϕ* and initial value $u_{2}=\left(x,y,z_{2}\right)\in D_{2}^{\circ }$. The dynamics of the system (25) is listed in the following claims in which the first two claims help determine exactly the support of the unique invariant probability measure ${\tilde{\mu }}_{4}$ of the system (5) in $D_{2}^{\circ }$ while the last two claims are used in the proofs of Theorem 3.3 and Theorem 3.4.

**Claim 3.5.** *Let* $\left(x_{0},y_{0},z_{20}\right)\in D_{2}^{\circ }$ *and z*_21_ ∈ (0, ∞). *Then*, *for any ϵ* > 0, *there are a control ϕ*(·) *and a time T* > 0 *such that*

$$\left|x_{\phi }\left(T,x_{0},y_{0},z_{20}\right)-x_{0}\right|<\epsilon ,$$

$$\left|y_{\phi }\left(T,x_{0},y_{0},z_{20}\right)-y_{0}\right|<\epsilon ,\;and$$

$$z_{2\phi }\left(T,x_{0},y_{0},z_{20}\right)=z_{21}.$$

**Claim 3.6.** *Let* (*x*_0_, *y*_0_) ∈ Δ° *and z*_20_ ∈ (0, ∞).

- *If z*_20_ ∈ (0, z**), *where* $z^{**}≔\frac{r(a-1)}{al_{2}}$, *then for any ϵ* > 0 *there exist a control ϕ*(·) *and a time T* > 0 *so that*
$$\left|x_{\phi }\left(T,x_{0},y_{0},z_{20}\right)-x^{**}\right|<\epsilon ,$$
$$\left|y_{\phi }\left(T,x_{0},y_{0},z_{20}\right)-y^{**}\right|<\epsilon ,\;and$$
$$z_{2\phi }\left(t,x_{0},y_{0},z_{20}\right)=z_{20}\;\forall t\in [0,T]$$
  *in which* $x^{**}≔\frac{1}{a}$ *and* $y^{**}≔y_{1}^{*}-\frac{l_{2}z_{20}}{a+r}$.
- *If* $z_{20}\in \left[z^{**},\frac{r}{l_{2}}\right)$ *then for each ϵ* > 0 *there are a control ϕ*(·) *and a time T* > 0 *such that*
$$\left|x_{\phi }\left(T,x_{0},y_{0},z_{20}\right)-\hat{x}\right|<\epsilon ,$$
$$y_{\phi }\left(T,x_{0},y_{0},z_{20}\right)<\epsilon ,\;and$$
$$z_{2\phi }\left(t,x_{0},y_{0},z_{20}\right)=z_{20}\;\forall t\in [0,T]$$
  *where* $\hat{x}≔\frac{r-l_{2}z_{20}}{r}$.
- *If* $z_{20}\geq \frac{r}{l_{2}}$ *then for each ϵ* > 0 *there are a control ϕ*(·) *and a time T* > 0 *such that*
$$x_{\phi }\left(T,x_{0},y_{0},z_{20}\right)<\epsilon ,$$
$$y_{\phi }\left(T,x_{0},y_{0},z_{20}\right)<\epsilon ,\;and$$
$$z_{2\phi }\left(t,x_{0},y_{0},z_{20}\right)=z_{20}\;\forall t\in [0,T].$$

**Claim 3.7.** *For any* $u_{2}=\left(x,y,z_{2}\right)\in D_{2}^{\circ }$, *there is a point* $\left(x_{3}^{*},y_{3}^{*},z_{2}^{*}\right)\in D_{2}^{\circ }$ *with the following properties: if* $0<\delta <\text{min}\left\{x_{3}^{*},y_{3}^{*},\frac{1}{\sqrt{2}}\left(x_{3}^{*}+y_{3}^{*}\right),z_{2}^{*}\right\}$ *and let*

$$V_{\delta }^{\prime }≔\left(x_{3}^{*}-\delta ,x_{3}^{*}+\delta \right)\times \left(y_{3}^{*}-\delta ,y_{3}^{*}+\delta \right)\times \left(z_{2}^{*}-\delta ,z_{2}^{*}+\delta \right),\text{ then}$$

- *there are a control ϕ*(·) *and a time T* > 0 *so that* $\left(x_{\phi }\left(T,u_{2}\right),y_{\phi }\left(T,u_{2}\right),z_{2\phi }\left(T,u_{2}\right)\right)\in V_{\delta }^{\prime }$,
- *there exist a neighborhood* $S_{\delta }^{\prime }\subset V_{\delta }^{\prime }$ *and a control ϕ*(·) *such that* $S_{\delta }^{\prime }$ *is invariant under the system* (25), *that is*, *for all t* ≥ 0 *and* $u_{2}\in S_{\delta }^{\prime }$, $\left(x_{\phi }\left(t,u_{2}\right),y_{\phi }\left(t,u_{2}\right),z_{2\phi }\left(t,u_{2}\right)\right)\in S_{\delta }^{\prime }$.

**Claim 3.8.** *For any* $u_{2}=\left(x,y,z_{2}\right)\in D_{2}^{\circ }$ *and for any* $0<\delta <\text{min}\left\{x_{1}^{*},y_{1}^{*},\frac{1}{\sqrt{2}}\left(x_{1}^{*}+y_{1}^{*}\right)\right\}$, *there are a control ϕ*(·) *and a time T* > 0 *such that*

$$\left(x_{\phi }\left(T,u_{2}\right),y_{\phi }\left(T,u_{2}\right),z_{2\phi }\left(T,u_{2}\right)\right)\in W_{\delta }≔\left(x_{1}^{*}-\delta ,x_{1}^{*}+\delta \right)\times \left(y_{1}^{*}-\delta ,y_{1}^{*}+\delta \right)\times (0,\delta ).$$

To prove Theorem (3.3), we also need the following lemma.

**Lemma 3.4.** *The solution process* $U_{2}^{u_{2}}(t)$ *of the system* (5) *is a T-process*. *Moreover*, *every compact set* $K\subset D_{2}^{\circ }$ *is petite for the Markov chain* (*x*(*n*), *y*(*n*), *z*_2_(*n*)), n∈ℕ.

The proof of Lemma 3.4 is completely similar to that of Lemma 3.2. The first conclusion follows from Lemma 3.3 while we can use Claim 3.7 to derive the second one. Now we map out key points in the proofs of Theorem 3.3 and Theorem 3.4.

**Proof of Theorem 3.3.** First, since $\lambda_{2}=e_{2}y_{1}^{*}-d_{2}-\frac{1}{2}\tau_{2}^{2}>0$, there is a *q* ∈ (0, 1) such that $e_{2}y_{1}^{*}-d_{2}-\frac{1}{2}\tau_{2}^{2}(q+1)>0$. Next, we show that there exists a δ** > 0 such that $\underset{t\to \infty }{\text{lim}}x(t)\geq \delta^{**}$ a.s. and $\underset{t\to \infty }{\text{lim}}z_{2}(t)\geq \delta^{**}$ a.s. Then consider the system (5) in the invariant domain ${ℳ}_{2}=\left\{\left(x,y,z_{2}\right)\in D_{2}^{\circ }:x\geq \delta^{**}\text{ and }z_{2}\geq \delta^{**}\right\}$ and consider the function *V*_7_ on ℳ_2_ defined by

$$V_{7}\left(x,y,z_{2}\right)=\frac{e_{2}}{l_{2}\delta^{**}}x+z_{2}^{-q}+z_{2}+1.$$

It is easy to show that *V* is a positive norm-like function on ℳ_2_ that satisfies ℒ*V*_7_ ≤ *θ*_5_*V*_7_ + *θ*_6_ for some *θ*_5_ < 0 and *θ*_6_ < ∞. By Lemma 3.4, applying Theorem 4 in Appendix A.3 together with Claims 3.5, 3.6, and 3.7 gives part (i) and part (ii) of Theorem 3.3. Part (iii) follows from Lemma 3.4 and Theorem 3 in Appendix A.3. □

**Proof of Theorem 3.4.** The proof can be carried out in 3 main steps.

- Show $\left(x_{1}^{*},y_{1}^{*},0\right)$ is an asymptotically stable in probability equilibrium for the system (5) using Lyapunov function method.
- Prove $U_{2}^{u_{2}}(t)$ is recurrent relative to some compact set $\bar{K}$ in $D_{2}^{\circ }$ by considering the function *V*_8_ on $D_{2}^{\circ }$ given by $V_{8}\left(x,y,z_{2}\right)=\frac{1}{R}x+y+\frac{1}{e_{2}}\text{log}\left(1+z_{2}\right)$ where *R* is chosen so that $\frac{d_{2}}{e_{2}}-\frac{r}{R}>0$ Then it is easy to show that ℒ*V*_8_ < 0 for any $\left(x,y,z_{2}\right)\in {\bar{K}}^{c}$ in which $\bar{K}=\left\{\left(x,y,z_{2}\right)\in D_{2}^{\circ }:y+\left(\frac{d_{2}}{e_{2}}-\frac{r}{R}\right)z_{2}\leq \frac{r}{R}\right\}$.
- Use the strong Markov property of $U_{2}^{u_{2}}(t)$, the support theorem, and Claim 3.8 to derive the desired result. □

### 3.3. Proof of Theorem 2.3

As in subsection 3.2, we present all lemmas and claims without their proofs that are necessary for proving Theorem 3.5 and Theorem 3.6. Since the proof of Theorem 3.6 is similar as that of Theorem 3.2, we skip details and only sketch main points in the proof of Theorem 3.5. Note that we always assume *a* > 1.

First, we rewrite the system (3) in the Stratonovich form

(26) $$dx=\left[rx(1-x-y)-axy-l_{2}xz_{2}\right]dt,\;dy=\left(axy-l_{1}yz_{1}-y\right)dt,\;dz_{1}=\left(e_{1}y-d_{1}-\frac{\tau_{1}^{2}}{2}\right)z_{1}dt+\tau_{1}z_{1}\circ dW_{1},\;dz_{2}=\left(e_{2}y-d_{2}-\frac{\tau_{2}^{2}}{2}\right)z_{2}dt+\tau_{2}z_{2}\circ dW_{2}.$$

Let

$$\bar{f}=\left(\begin{array}{c} rx(1-x-y)-axy-l_{2}xz_{2}\\ axy-l_{1}yz_{1}-y\\ e_{1}yz_{1}-d_{1}z_{1}-\frac{1}{2}\tau_{1}^{2}z_{1}\\ e_{2}yz_{2}-d_{2}z_{2}-\frac{1}{2}\tau_{2}^{2}z_{2} \end{array}\right),g_{1}=\left(\begin{array}{c} 0\\ 0\\ \tau_{1}u_{1}\\ 0 \end{array}\right)\text{ and }g_{2}=\left(\begin{array}{c} 0\\ 0\\ 0\\ \tau_{2}u_{2} \end{array}\right).$$

By computation, we can check that *g*_1_, *g*_2_, $\left[\bar{f},g_{1}\right]$, and $\left[\bar{f},g_{2}\right]$ span ${\mathbb{R}}^{4}$ for every point *u* = (*x*, *y*, *z*_1_, *z*_2_) ∈ *D*°. Hence we get the following lemma.

**Lemma 3.5.** *The solution U*^*u*^(*t*) *to the system* (3) *in D*° *satisfies Hörmander’s condition*. *Therefore its transition probability P*(*t*, *u*_0_, ·) *has density p*(*t*, *u*_0_, *u*) *which is smooth in* (*u*_0_, *u*) ∈ *D*° × *D*°.

Next we consider the control system corresponding to the system (26)

(27) $${\dot{x}}_{\phi }=rx_{\phi }\left(1-x_{\phi }-y_{\phi }\right)-ax_{\phi }y_{\phi }-l_{2}x_{\phi }z_{2\phi },\;{\dot{y}}_{\phi }=ax_{\phi }y_{\phi }-l_{1}y_{\phi }z_{1\phi }-y_{\phi },\;{\dot{z}}_{1\phi }=\left(e_{1}y_{\phi }-d_{1}-\frac{\tau_{1}^{2}}{2}+\tau_{1}\phi_{1}\right)z_{1\phi },\;{\dot{z}}_{2\phi }=\left(e_{2}y_{\phi }-d_{2}-\frac{\tau_{2}^{2}}{2}+\tau_{2}\phi_{2}\right)z_{2\phi },$$

where *ϕ* = *ϕ*(*t*) = (*ϕ*_1_(*t*), *ϕ*_2_(*t*)) is from the set of piecewise continuous functions defined on ${\mathbb{R}}_{+}$ taking values on ${\mathbb{R}}^{2}$. Let (*x*_*ϕ*_(*t*, *u*), *y*_*ϕ*_(*t*, *u*), *z*_1*ϕ*_(*t*, *u*), *z*_2*ϕ*_(*t*, *u*)) be the solution to the system (27) with control *ϕ* = (*ϕ*_1_, *ϕ*_2_) and initial value *u* = (*x*, *y*, *z*_1_, *z*_2_) ∈ *D*°.

Now we assume that $\frac{d_{1}}{e_{1}}+\frac{\tau_{1}^{2}}{2e_{1}}=\frac{d_{2}}{e_{2}}+\frac{\tau_{2}^{2}}{2e_{2}}$. From the last two equations of (27), we get

$$\frac{{\dot{z}}_{2\phi }}{z_{2\phi }}=\frac{e_{2}}{e_{1}}\frac{{\dot{z}}_{1\phi }}{z_{1\phi }}+e_{2}\left(\frac{d_{1}}{e_{1}}+\frac{\tau_{1}^{2}}{2e_{1}}-\frac{d_{2}}{e_{2}}-\frac{\tau_{2}^{2}}{2e_{2}}\right)+\tau_{2}\phi_{2}-\frac{e_{2}}{e_{1}}\tau_{1}\phi_{1}.$$

Set $\rho =\frac{e_{2}}{e_{1}}$, then we obtain $\frac{{\dot{z}}_{2\phi }}{z_{2\phi }}=\rho \frac{{\dot{z}}_{1\phi }}{z_{1\phi }}+\tau_{2}\phi_{2}-\rho \tau_{1}\phi_{1}$. Integrating both sides from 0 to *t* gives

$$z_{2\phi }(t)=kz_{1\phi }^{\rho }(t)\text{ exp }\left\{\overset{t}{\underset{0}{\int }}\left[\tau_{2}\phi_{2}(s)-\rho \tau_{1}\phi_{1}(s)\right]ds\right\},$$

where $k≔z_{2\phi }(0)/\left(z_{1\phi }^{\rho }(0)\right)$ Given *ϕ*_1_, we choose the control *ϕ*_2_ such that $\phi_{2}\equiv \rho \frac{\tau_{1}}{\tau_{2}}\phi_{1}$. Then $z_{2\phi }(t)=kz_{1\phi }^{\rho }(t)$ for all *t* ≥ 0. Thus, with the choice of control *ϕ*_2_ above, for each *k* ∈ (0, ∞) fixed the system (27) is reduced to a 3-dim control system with one control *ϕ*_1_

(28) $${\dot{x}}_{\phi }=rx_{\phi }\left(1-x_{\phi }-y_{\phi }\right)-ax_{\phi }y_{\phi }-kl_{2}x_{\phi }z_{1\phi }^{\rho },\;{\dot{y}}_{\phi }=ax_{\phi }y_{\phi }-l_{1}y_{\phi }z_{1\phi }-y_{\phi },\;{\dot{z}}_{1\phi }=\left(e_{1}y_{\phi }-d_{1}-\frac{\tau_{1}^{2}}{2}+\tau_{1}\phi_{1}\right)z_{1\phi }.$$

We denote by (*x*_*ϕ*_(*t*, *u*), *y*_*ϕ*_(*t*, *u*), *z*_1*ϕ*_(*t*, *u*)) the solution of the system (28) with control *ϕ*_1_ and initial value $u=\left(x,y,z_{1}\right)\in D_{1}^{\circ }$. The dynamics of the system (28) is presented in the following claims in which the first two claims determine exactly the supports of the collection of invariant probability measures {*π*(*k*^)^}_*k*∈(0,∞)_ of the system (3) in *D*° while the last two claims are used in the proofs of Theorem 3.5 and Theorem 3.6.

**Claim 3.9.** *Let* $\left(x_{0},y_{0},z_{10}\right)\in D_{1}^{\circ }$ *and z*_11_ ∈ (0, ∞). *Then*, *for any ϵ* > 0, *there are a control ϕ*_1_(·) *and a time T* > 0 *such that*

$$\left|x_{\phi }\left(T,x_{0},y_{0},z_{10}\right)-x_{0}\right|<\epsilon ,$$

$$\left|y_{\phi }\left(T,x_{0},y_{0},z_{10}\right)-y_{0}\right|<\epsilon ,$$

$$z_{1\phi }\left(T,x_{0},y_{0},z_{10}\right)=z_{11}.$$

**Claim 3.10.** *Let* (*x*_0_, *y*_0_) ∈ Δ° *and z*_10_ ∈ (0, ∞). *Let*

$$\Theta ≔\left\{z_{10}\in (0,\infty ):l_{1}z_{10}+\frac{kal_{2}}{r}z_{10}^{\rho }<a-1\right\}.$$

- *If z*_10_ ∈ Θ *then for any ϵ* > 0 *there exist a control ϕ*_1_(·) *and a time T* > 0 *so that*
$$\left|x_{\phi }\left(T,x_{0},y_{0},z_{10}\right)-X^{*}\left(k,z_{10}\right)\right|<\epsilon ,$$
$$\left|y_{\phi }\left(T,x_{0},y_{0},z_{10}\right)-Y^{*}\left(k,z_{10}\right)\right|<\epsilon ,$$
$$z_{1\phi }\left(t,x_{0},y_{0},z_{10}\right)=z_{10}\forall t\in [0,T],$$
  *where* $X^{*}\left(k,z_{10}\right)≔\frac{1+l_{1}z_{10}}{a}$, *and* $Y^{*}\left(k,z_{10}\right)≔y_{1}^{*}-\frac{rl_{1}z_{10}}{a(a+r)}-\frac{kl_{2}z_{10}^{\rho }}{a+r}$.
- *Assume that z*_10_ ∉ Θ. *Then*
  (iia) *if* $\left(z_{10}\geq z^{*},kz_{10}^{\rho }<\frac{r}{l_{2}}\right)\vee \left(z_{10}<z^{*},z^{**}\leq kz_{10}^{\rho }<\frac{r}{l_{2}}\right)$ *then for each ϵ* > 0 *there are a control ϕ*_1_(·) *and a time T* > 0 *such that*
$$\left|x_{\phi }\left(T,x_{0},y_{0},z_{10}\right)-\bar{x}\right|<\epsilon ,$$
$$y_{\phi }\left(T,x_{0},y_{0},z_{10}\right)<\epsilon ,$$
$$z_{1\phi }\left(t,x_{0},y_{0},z_{10}\right)=z_{10}\forall t\in [0,T],$$
  *where* $\bar{x}=1-\frac{kl_{2}z_{1}^{\rho }}{r}$.
  (iib) *if* $kz_{10}^{\rho }\geq \frac{r}{l_{2}}$ *then for each ϵ* > 0 *there are a control ϕ*_1_(·) *and a time T* > 0 *such that*
$$x_{\phi }\left(T,x_{0},y_{0},z_{10}\right)<\epsilon ,$$
$$y_{\phi }\left(T,x_{0},y_{0},z_{10}\right)<\epsilon ,$$
$$z_{1\phi }\left(t,x_{0},y_{0},z_{10}\right)=z_{10}\;\forall t\in [0,T].$$

**Claim 3.11.** *For any* $u=\left(x,y,z_{1}\right)\in D_{1}^{\circ }$, *there is a point* $\left(x_{4}^{*},y_{4}^{*},z_{3}^{*}\right)\in D_{1}^{\circ }$ *with the following properties: if* $0<\delta <\text{min }\left\{x_{4}^{*},y_{4}^{*},\frac{1}{\sqrt{2}}\left(x_{4}^{*}+y_{4}^{*}\right),z_{3}^{*}\right\}$ *and let*

$$V_{\delta }^{\prime \prime }≔\left(x_{4}^{*}-\delta ,x_{4}^{*}+\delta \right)\times \left(y_{4}^{*}-\delta ,y_{4}^{*}+\delta \right)\times \left(z_{3}^{*}-\delta ,z_{3}^{*}+\delta \right),\text{ then}$$

- *there are a control ϕ*_1_(·) *and a time T* > 0 *so that* $\left(x_{\phi }(T,u),y_{\phi }(T,u),z_{1\phi }(T,u)\right)\in V_{\delta }^{\prime \prime }$,
- *there exist a neighborhood* $S_{\delta }^{\prime \prime }\subset V_{\delta }^{\prime \prime }$ *and a control ϕ*_1_(·) *such that* $S_{\delta }^{\prime \prime }$ *is invariant under the system* (28), *that is*, *for all t* ≥ 0 *and* $u\in S_{\delta }^{\prime \prime }$, $\left(x_{\phi }(t,u),y_{\phi }(t,u),z_{1\phi }(t,u)\right)\in S_{\delta }^{\prime \prime }$.

**Claim 3.12.** *For any* $u=\left(x,y,z_{1}\right)\in D_{1}^{\circ }\;$ *and for any* $0<\delta <\text{min}\left\{x_{1}^{*},y_{1}^{*},\frac{1}{\sqrt{2}}\left(x_{1}^{*}+y_{1}^{*}\right)\right\}$, *there are a control ϕ*_1_(·) *and a time T* > 0 *such that*

$$\left(x_{\phi }(T,u),y_{\phi }(T,u),z_{1\phi }(T,u)\right)\in W_{\delta }≔\left(x_{1}^{*}-\delta ,x_{1}^{*}+\delta \right)\times \left(y_{1}^{*}-\delta ,y_{1}^{*}+\delta \right)\times (0,\delta ).$$

Now we sketch the proof of Theorem 3.5 with key points.

**Proof of Theorem 3.5.** Since $\frac{d_{1}}{e_{1}}+\frac{\tau_{1}^{2}}{2e_{1}}=\frac{d_{2}}{e_{2}}+\frac{\tau_{2}^{2}}{2e_{2}}$, the last two equations of (3) imply (8). For each *k* ∈ (0, ∞) fixed, due to (8) the long-term behavior of (3) is reduced to that of the following 3-dim SDE system

(29) $$dx=\left[rx(1-x-y)-axy-kl_{2}xz_{1}^{\rho }e^{\tau_{2}W_{2}-\rho \tau_{1}W_{1}}\right]dt,\;dy=\left(axy-l_{1}yz_{1}-y\right)dt,\;dz_{1}=\left(e_{1}yz_{1}-d_{1}z_{1}\right)dt+\tau_{1}z_{1}dW_{1},$$

with the a.s. invariant domain *D*_1_. Let $U_{3}^{u_{3}}(t)=\left(x(t),y(t),z_{1}(t)\right)$ be the solution to (29) with initial condition $u_{3}=\left(x,y,z_{1}\right)\in D_{1}^{\circ }$. With the same reasoning as in the proof of Lemma 3.2, $U_{3}^{u_{3}}(t)$ is a *T*-process and every compact set in $D_{1}^{\circ }$ is petite for the Markov chain $U_{3}^{u_{3}}(n)(n\in \mathbb{N})$. Next, we can show that $U_{3}^{u_{3}}(t)$ is recurrent relative to some compact set in $D_{1}^{\circ }$ by looking at the positive function $V_{9}\left(x,y,z_{1}\right)=x+y+\frac{l_{1}}{e_{1}}z_{1}$. Lastly, since $\lambda =y_{1}^{*}-h>0$, $e_{1}y_{1}^{*}-d_{1}-\frac{1}{2}\tau_{1}^{2}>0$. So, with the same reasoning as in Theorem 3.1, we can derive all the conclusions of Theorem 3.5. □

## 4. Numerical study and discussion

### 4.1. Numerical demonstrations of stochastic bifurcations without parameters

From our analysis, our stochastic model has a collection of invariant probability measures indexed by a real number between 0 and ∞ for which each invariant probability measure is supported by an open line segment in 4-dimensional space. It suggests that our stochastic model should undergo some kind of stochastic bifurcation that is similar to the Poincare-Andronov-Hopf bifurcation without parameters in our deterministic setting [23]. In the book [1], there are two types of stochastic bifurcations that are studied so far. The first type is phenomenological bifurcation (or P-bifurcation) which is concerned with the change in the shape of density functions of a family of invariant probability measures in a stochastic system as one of its parameter changes. The second one is dynamical bifurcation (or D-bifurcation) which is characterized by sign changes of Lyapunov exponents of a family of invariant probability measures in a stochastic system as one of its parameters changes. To study a P-bifurcation, we compute the density functions of invariant probability measures of the stochastic system by solving the corresponding Fokker-Planck equations, and observe their shape change when one of the system parameters changes. If the density functions’ shape switches from one peak into crater, then the stochastic system admits a stochastic Hopf bifurcation in phenomenological sense [1,11]. For a D-bifurcation, we can verify it by computing the system’s Lyapunov exponents. If there is a stochastic Hopf bifurcation in dynamical sense, then it is necessary that one Lyapunov exponent has to pass through zero [14]. Furthermore, if one of the invariant probability measures of the stochastic system loses its stability and becomes unstable, and the global attractors of the stochastic system change from a single-point set into a random topological disk, then the stochastic Hopf bifurcation in dynamical sense is admitted [11].

Due to the high dimensionality of our stochastic system, solving its corresponding Fokker-Planck system explicitly is almost impossible, and the collection of invariant probability measures in Theorem 2.3 can not be found explicitly. Thus, in this subsection, we aim to demonstrate numerically stochastic Hopf bifurcations without parameters for our stochastic system in both phenomenological and dynamical point of views. To do so, we consider the non-dimensionalized stochastic system (3) and assume that $\frac{d_{1}}{e_{1}}+\frac{\tau_{1}^{2}}{2e_{1}}=\frac{d_{2}}{e_{2}}+\frac{\tau_{2}^{2}}{2e_{2}}≕h$. Notice that *λ* = 0 is equivalent to $m(a)≔a^{2}+r\left(1-\frac{1}{h}\right)a+\frac{r}{h}=0$. If *h* < 1 and $r>\frac{4h}{{\left(1-h\right)}^{2}}$ then the discriminant of *m*(*a*) is positive and hence *m*(*a*) = 0 has 2 positive roots

$$a_{1,2}≔\frac{1}{2}r\left(\frac{1}{h}-1\right)\mp \frac{1}{2}\sqrt{r^{2}{\left(\frac{1}{h}-1\right)}^{2}-\frac{4r}{h}}$$

with 1 < *a*_1_ < *a*_2_. Hence *λ* = 0 is equivalent to either *a* = *a*_1_ or *a* = *a*_2_. From now on, we fix all parameters of the stochastic system (3) such that *h* < 1, $r>\frac{4h}{{\left(1-h\right)}^{2}}$, and *a* ∈ (*a*_1_, *a*_2_). This makes sure that *λ* > 0 and, by Theorem 3.5, the system (3) has a collection of exponentially ergodic invariant probability measures {*π*(*k*)}_*k*∈(0,∞)_ in which each *π*(*k*) is supported by

$$S(k)≔\left\{\left(\frac{l_{1}z_{1}+1}{a},\frac{r(a-1)}{a(a+r)}-\frac{rl_{1}z_{1}}{a(a+r)}-\frac{l_{2}kz_{1}^{\rho }}{a+r},z_{1},kz_{1}^{\rho }\right):z_{1}\in \left(0,\frac{a-1}{l_{1}}\right)\right\}.$$

We utilize some data from our previous research [27] to simulate our stochastic system. After non-dimensionalization, the parameters of the stochastic system (3) are *r* = 0.36, *a* = 5, *l*_1_ = *l*_2_ = 0.48, *e*_1_ = *e*_2_ = 10, *d*_1_ = *d*_2_ = 0.4, and *τ*_1_ = *τ*_2_ = 0.01. By computation, we obtain *h* = 0.04 < 1, $r-\frac{4h}{{\left(1-h\right)}^{2}}=0.1864>0$, *a*_1_ = 1.2116, *a*_2_ = 7.4273, and *λ* = 0.0137 > 0. Hence all the conditions for the existence of the collection of invariant probability measures *π*(*k*) are guaranteed. Since we conduct numerical simulations based on the non-dimensionalized system (3), the units of two types of tumor cells and two types of immune cells are not absolute number densities but relative numbers. So the quantities such as *x* and *y* are the percentage of the number densities of uninfected tumor cells and infected tumor cells, respectively. While the quantities such as *z*_1_ and *z*_2_ are the portion of the number densities of innate immune cells and adaptive immune cells over the tumor carrying capacity, respectively, and so we indicate them as relative innate and adaptive immune cells. For the time, it can be considered as relative time since *T* = *δt*. In all the figures below, the solution paths of the stochastic system (3) are simulated with initial values (0.5, 0.5, 0.01*k*, 0.01) for different values of *k* in [0.01, 10].

To demonstrate a stochastic Hopf P-bifurcation without parameters, we approximate stationary distributions of exponentially ergodic invariant probability measures {*π*(*k*)} for the system (3) by computing single trajectories for a long time with different values of *k*. Note that the initial value of each single trajectory depends on *k* and, for each *k*, the transition probability of each single trajectory converges weakly to an ergodic invariant probability measure *π*(*k*). Then, by strong law of large numbers, for *A* ∈ ℬ(*D*) we get

$$\mathbb{P}\left\{\underset{t\to \infty }{\text{lim}}\frac{1}{t}\overset{t}{\underset{0}{\int }}1_{A}\left(U^{u}(s)\right)ds=\pi (k)(A)\right\}=1.$$

**Fig. 1.**
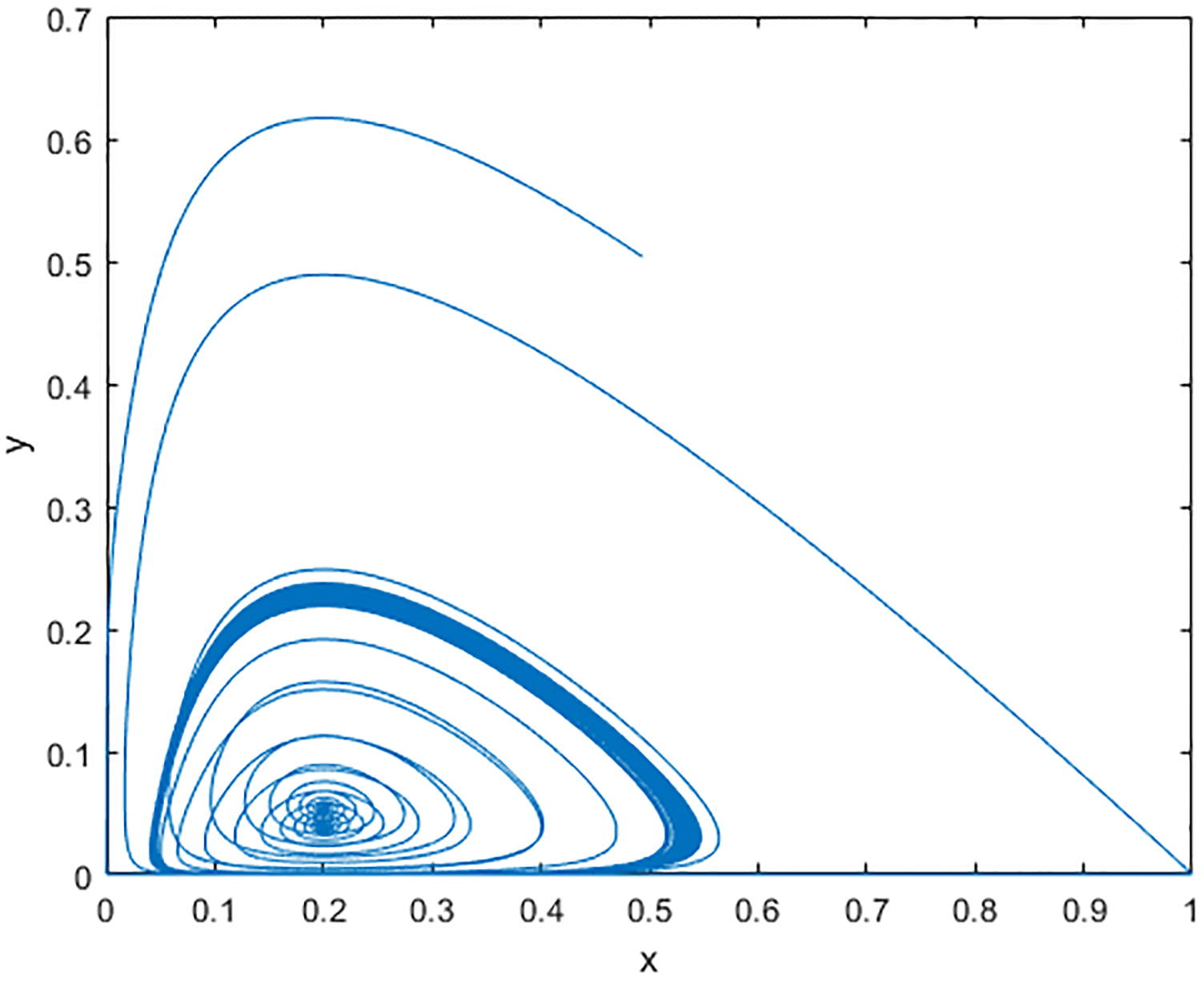

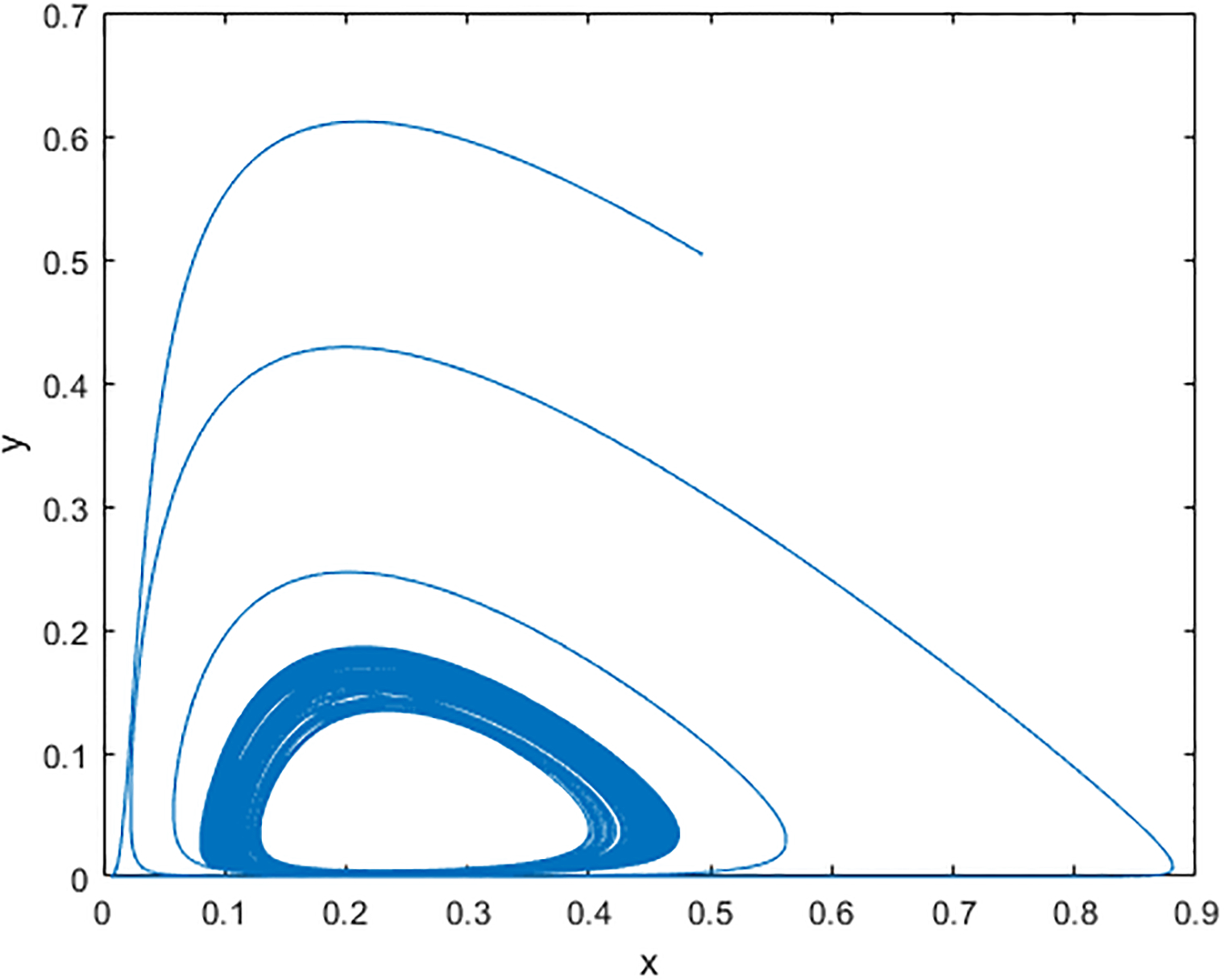

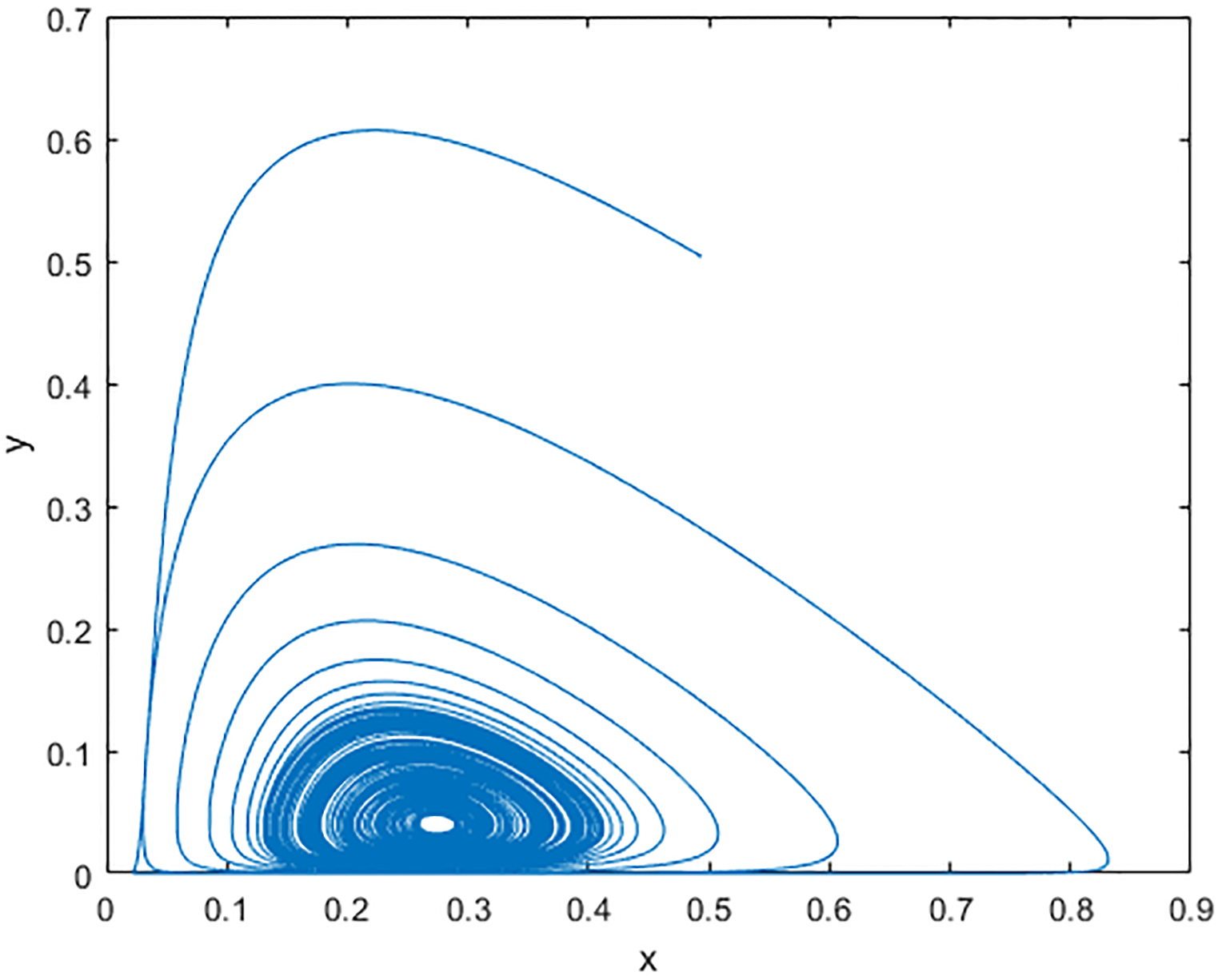

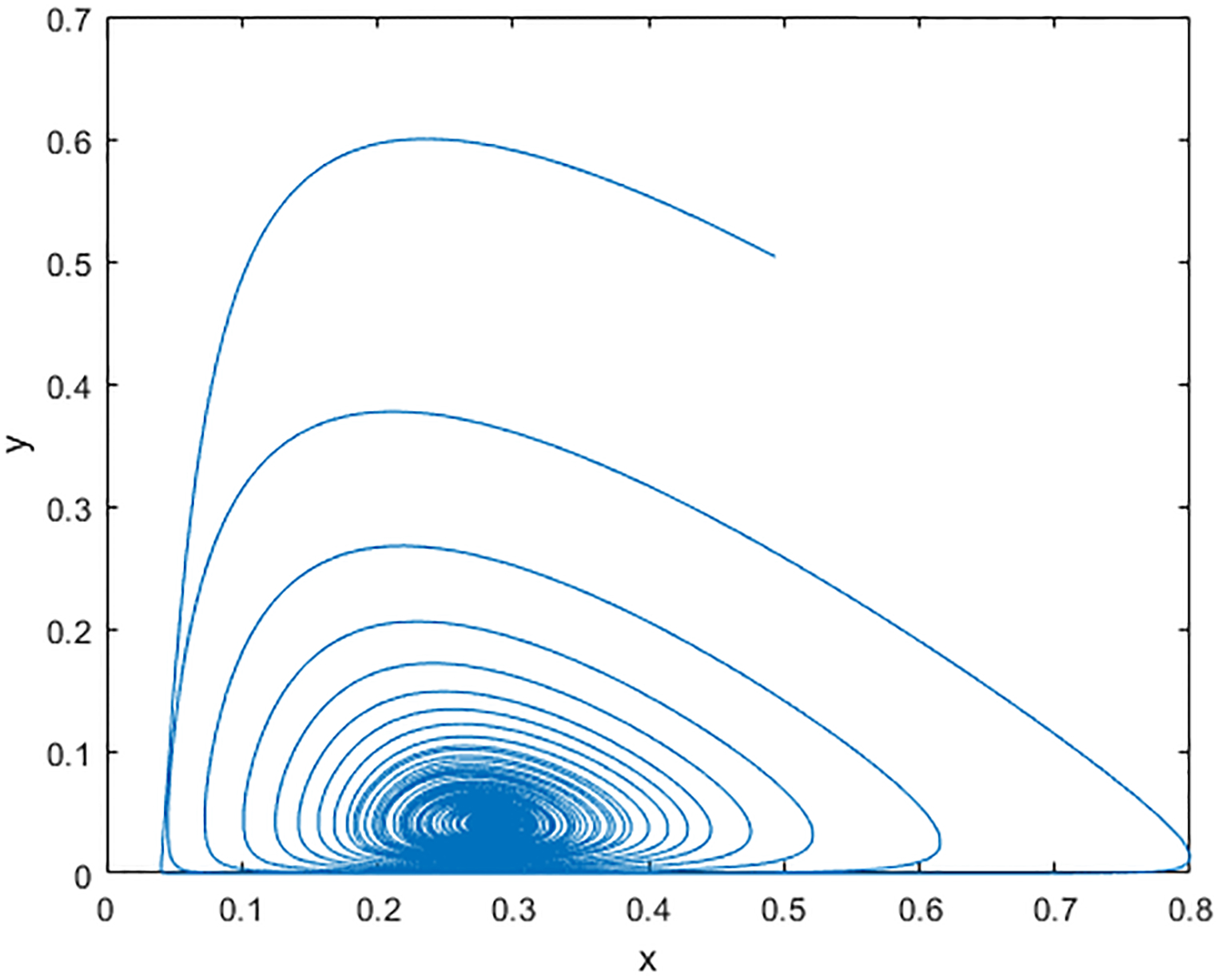
The *xy*-component solution path is simulated within a long time interval [0, 2000] when it starts at (0.5, 0.5, 0.01*k*, 0.01) with parameters *a* = 5, *r* = 0.36, *l*_1_ = *l*_2_ = 0.48, *e*_1_ = *e*_2_ = 10, *d*_1_ = *d*_2_ = 0.4, and *τ*_1_ = *τ*_2_ = 0.01.

**Fig. 2.**
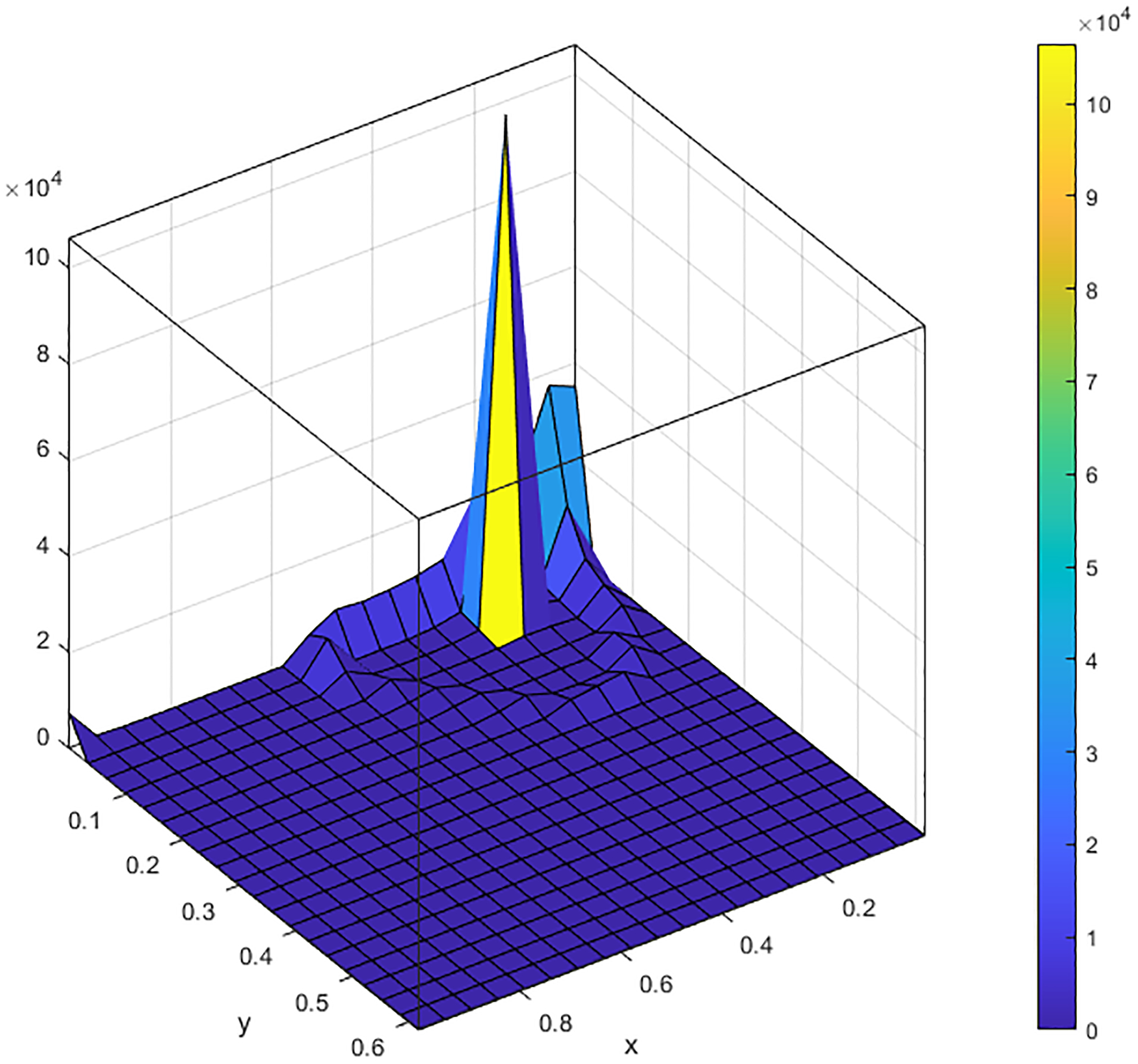

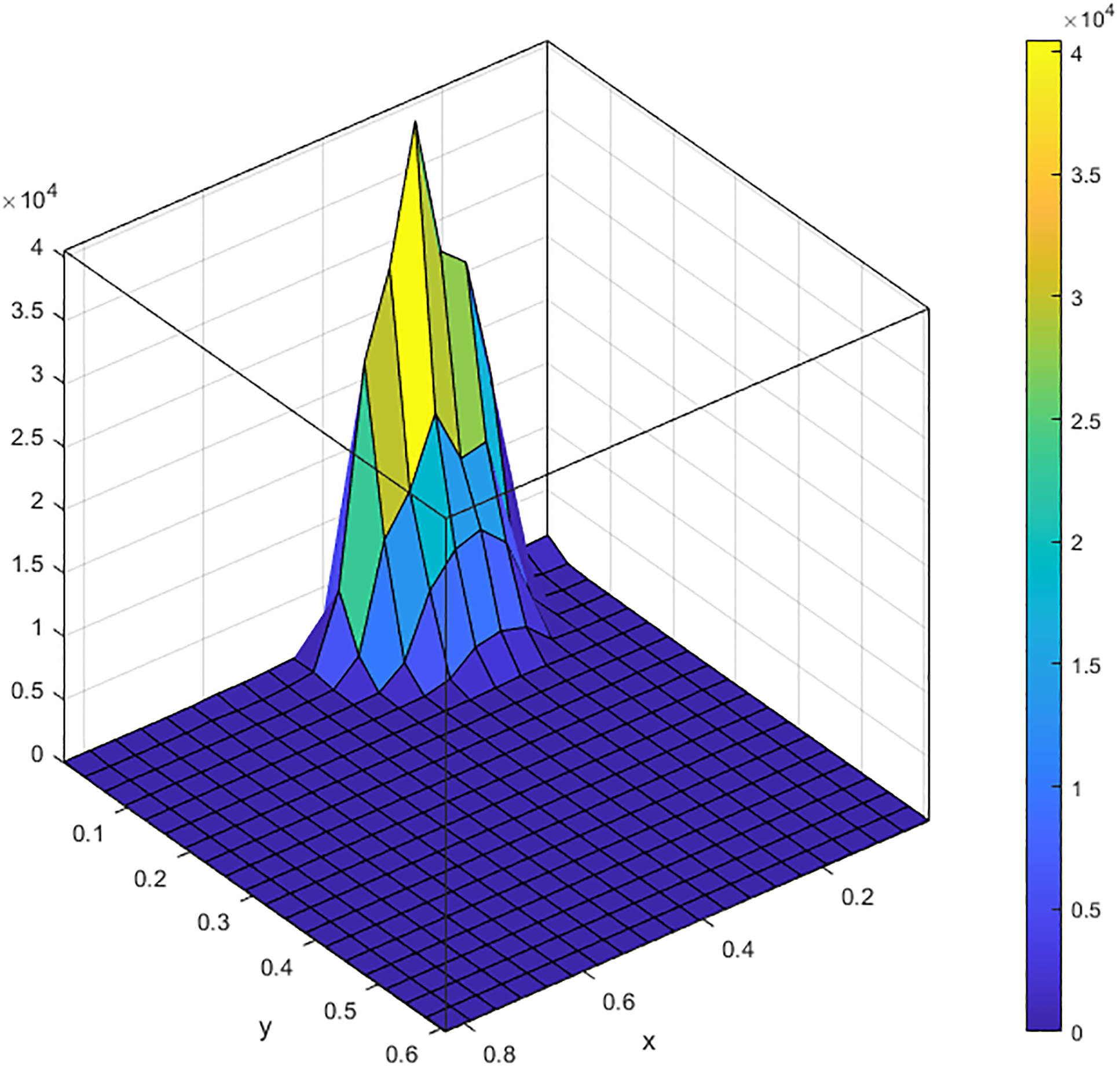

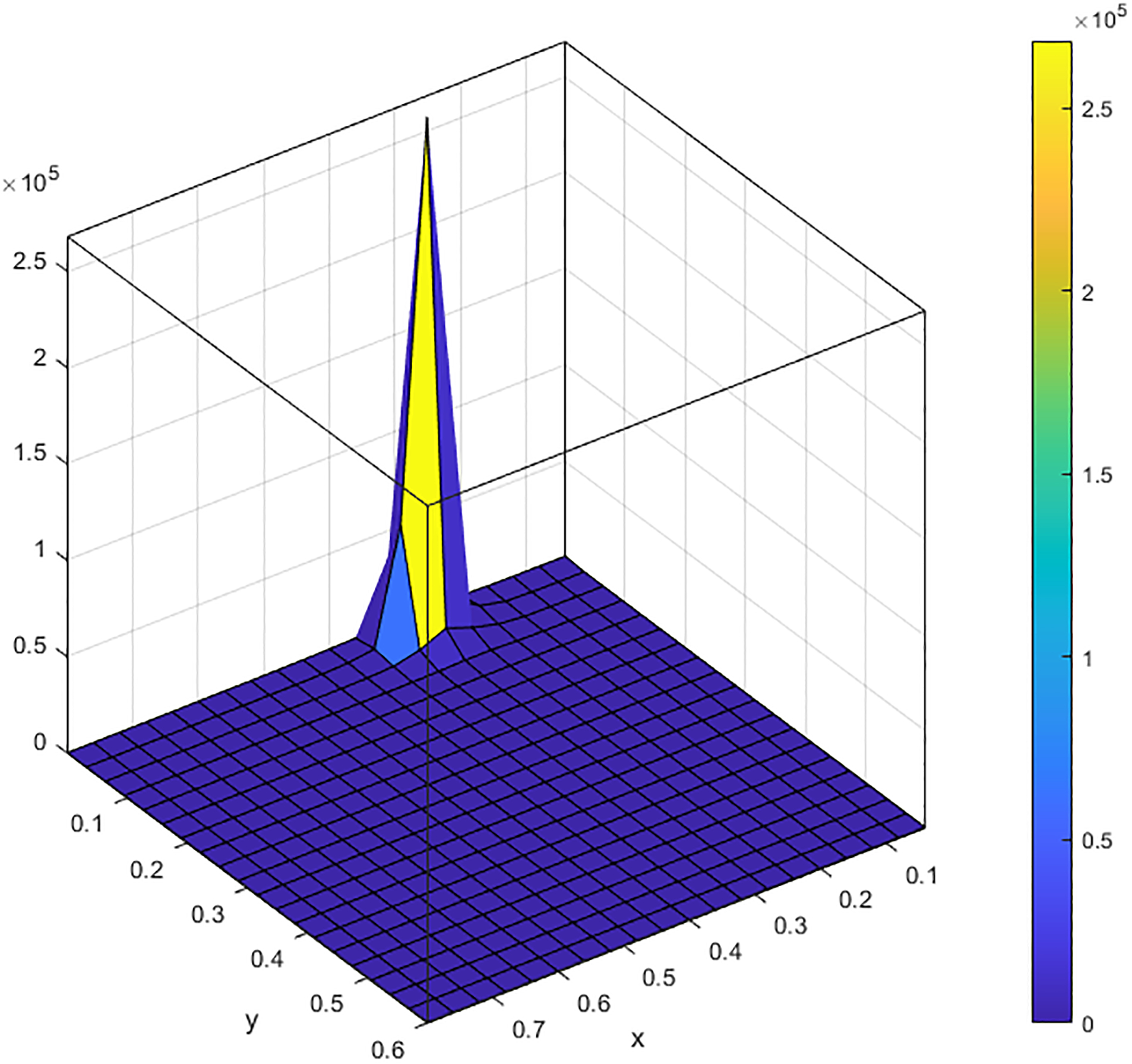

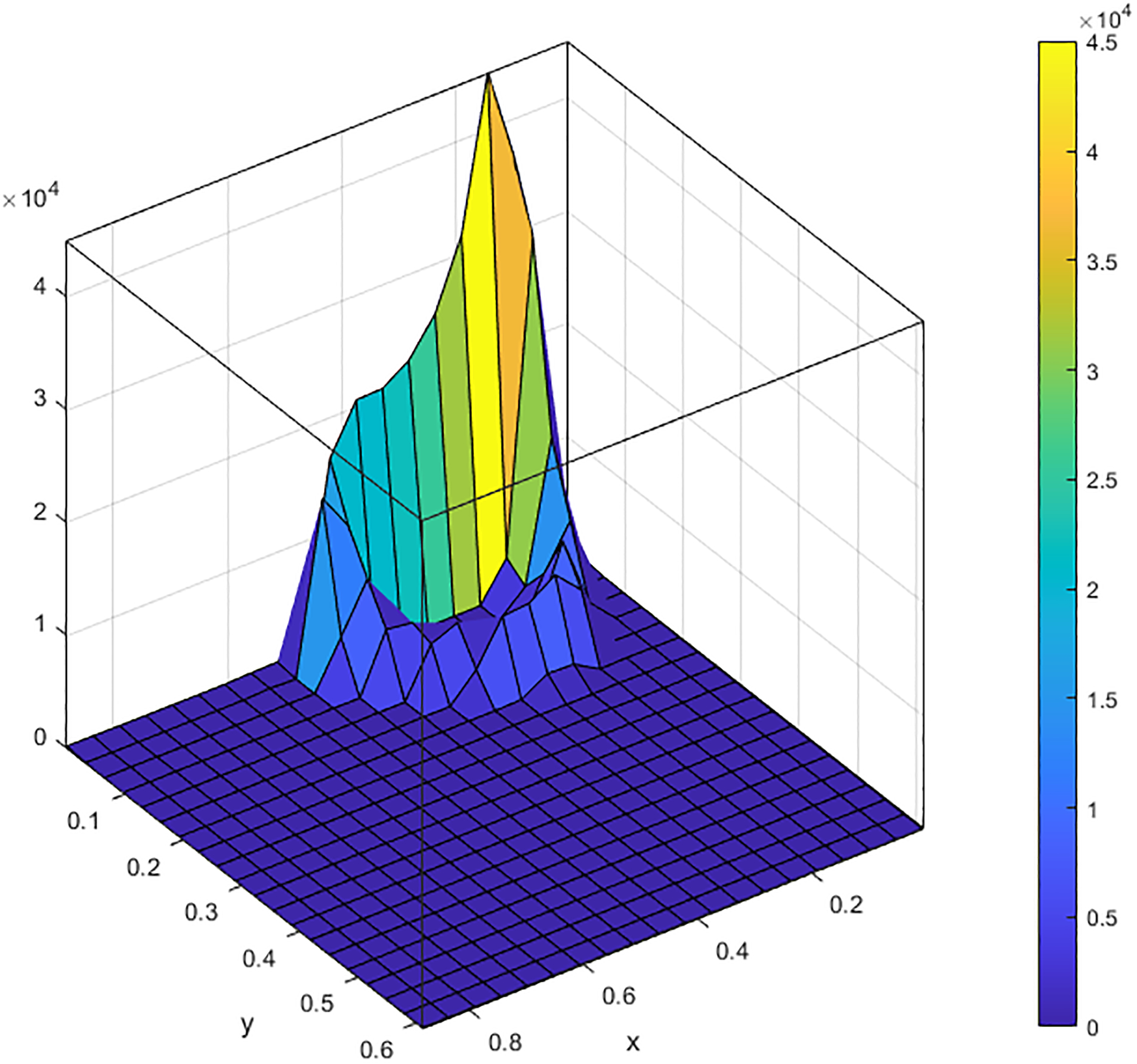
The solution path is simulated within a long time interval [0, 2000] when it starts at (0.5, 0.5, 0.01*k*, 0.01) with parameters *a* = 5, *r* = 0.36, *l*_1_ = *l*_2_ = 0.48, *e*_1_ = *e*_2_ = 10, *d*_1_ = *d*_2_ = 0.4, and *τ*_1_ = *τ*_2_ = 0.01. Four above figures are the corresponding histograms as a projection onto the *xy* components of the solution.

This means that, as time *t* is large enough, the relative occupation time of one single trajectory will approximate the density of the stationary distribution of *π*(*k*). Fig. 1 shows the *xy*-component projections of the solution paths within a long time interval [0, 2000] with 4 different values of *k*. Fig. 2 shows the corresponding histograms of these *xy*-component projections, which typically represent the densities of ergodic invariant measures *π*(*k*) with 4 different values of *k*. When *k* = 0.01, the shape of the invariant probability measure *π*(*k*) is one-peak mountain which means that the solution spends a lot of time around a small neighborhood of this peak. When we increase *k* to 2.5, the shape of *π*(*k*) looks like a crater. It explains that the solutions wander around some big region without ending up at one point. If we keep increasing *k* to 5, it is difficult to figure out that the shape of *π*(*k*) is one peak or crater-like mountain in Fig. 2(c) but, looking at Fig. 1(c), the behavior of the solution path is similar to that as *k* = 2.5. The solution path wanders around a small region without approaching a single point. When *k* = 9, we again obtain the one peak mountain shape of *π*(*k*). This demonstrates that the stochastic system (3) undergoes the stochastic Hopf P-bifurcation without parameters.

Next, to demonstrate a stochastic D-bifurcation without parameters, we numerically compute the Lyapunov exponents of all solution components of the system (3) when *k* changes between 0.01 and 10. Notice that, for each *k* ∈ [0.01, 10], when the solution path of the system (3) gets close to the support *S*(*k*), it will concentrate around this support for a long time. Hence the Lyapunov exponents with respect to *π*(*k*) can be computed as

(30) $$\lambda_{1}(\pi (k))\approx \frac{\text{ln }x(t)}{t},\lambda_{2}(\pi (k))\approx \frac{\text{ln }y(t)}{t},\lambda_{3}(\pi (k))\approx \frac{\text{ln }z_{1}(t)}{t},\text{ and }\lambda_{4}(\pi (k))\approx \frac{\text{ln }z_{2}(t)}{t}$$

for *t* large enough with the initial value (0.5, 0.5, 0.01*k*, 0.01).

**Fig. 3.**
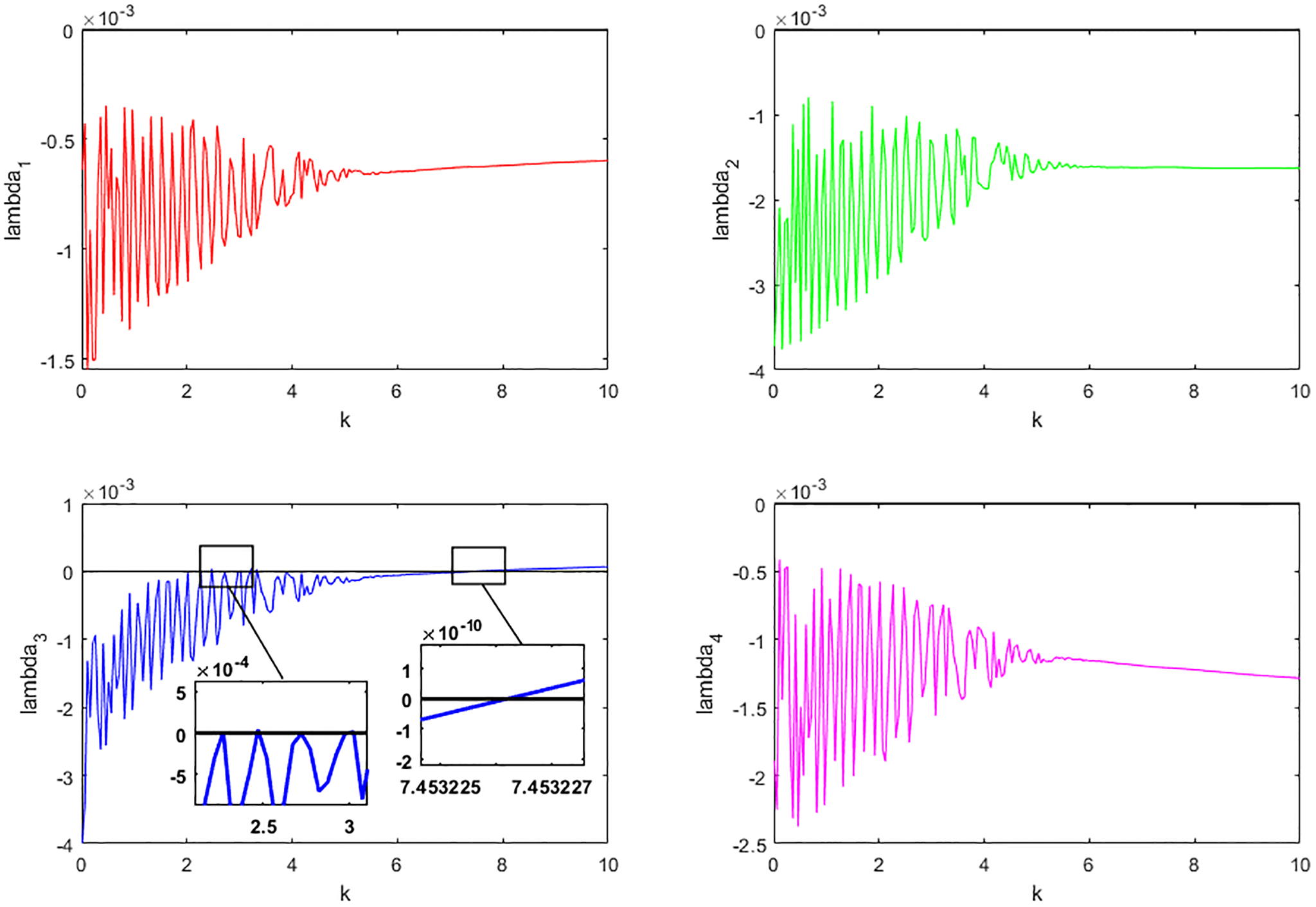
The Lyapunov exponents *λ*_*i*_ (*i* = 1, 2, 3, 4) of the corresponding component *x*, *y*, *z*_1_, and *z*_2_ of the solution are numerically computed as *k* changes from 0.01 to 10. The parameters are *a* = 5, *r* = 0.36, *l*_1_ = *l*_2_ = 0.48, *e*_1_ = *e*_2_ = 10, *d*_1_ = *d*_2_ = 0.4, and *τ*_1_ = *τ*_2_ = 0.01. The initial values are (0.5, 0.5, 0.01*k*, 0.01).

Fig. 3 shows the behavior of Lyapunov exponents *λ*_*i*_(*π*(*k*)) (*i* = 1, 2, 3, 4) of the four components of the solution to the system (3) when *k* runs through between 0.01 and 10. We see that three Lyapunov exponents *λ*_1_(*π*(*k*)), *λ*_2_(*π*(*k*)), and *λ*_4_(*π*(*k*)) are always below zero. However, the exponent *λ*_3_(*π*(*k*)), the blue curve, crosses zero several times at various values of *k* between 2 and 4. Then, it goes below zero until it hits zero again at some value of *k* between 6 and 8. Afterwards it lies above zero. This means that the invariant probability measure *π*(*k*) loses its stability and becomes unstable when *k* increases from 0.01 to 10. It shows that a stochastic Hopf D-bifurcation without parameters occurs in our stochastic setting.

To simulate numerically the non-dimensionalized system (2) with given initial values as in Fig. 1 and Fig. 2, we utilize the algorithm of stochastic Runge-Kutta method of strong order 1 proposed by Rößler (2010) [24]. Recall that an algorithm for simulating a stochastic differential equation (SDE) system can be derived from stochastic Taylor expansion of that SDE system by applying Ito’s formula to its drift term and diffusion term. The expansion includes four stochastic double integrals. If we cut off these four double integrals in that expansion then we obtain Euler-Maruyama (EM) method by discretizing the time interval and formulating the SDE system as a recursive algorithm. This method has strong convergence order 1/2 and weak convergence order 1. When we keep applying the Ito’s formula to the integrand of the last stochastic double integral, we obtain an expansion which includes one term with iterated Ito’s integral, three stochastic double integrals, and two stochastic triple integrals. If we discard these three double integrals and two triple integrals then we get the so-called Milstein method for the SDE system. The Milstein method has strong convergence order 1 and weak convergence order 1 (see Chapter 10 in [16]). However, to utilize this method, we have to compute all closed-form partial derivatives as well as iterated and cross-term Ito’s integrals arising from stochastic Taylor expansion. This makes the computation extremely expensive. Rößler (2010) developed a general class of stochastic Runge-Kutta schemes of strong order 1 in which computing these partial derivatives and the iterated Ito’s integrals are avoided in the scheme formulation and these Ito’s integrals only appear in the supporting values. We used a particular case from the general class to solve numerically our stochastic model (2). Furthermore, to make this algorithm more robust, we utilized EM method to simultaneously approximate all the four iterated Ito’s integrals in the formulation of the algorithm with sufficient accuracy. Also, we developed an algorithm of simulating the Lyapunov exponent for each solution component based on (30) when the value of *k* changes to produce Fig. 3. The details of these two algorithms and their Matlab codes can be found in the Supplemental Materials.

### 4.2. Discussion

In this part II of our research, we analyze our stochastic model. Stochasticities of our system come from immune cells and their microenvironments as the way we construct the model. For the parameters which are not affected by these stochasticities, for example, the virus infectivity constant *a*, they still play similar roles in overall dynamics of the stochastic system as in the deterministic counterpart. For 0 < *a* < 1, the ergodic invariant probability measures *μ*_1_, ${\bar{\mu }}_{1}$, and ${\tilde{\mu }}_{1}$ are global attractors, which means the treatments fail completely. For *a* > 1, these stochasticities or uncertainties come to play roles. Instead the relative immune clearance rates $\frac{d_{i}}{e_{i}}$ in the deterministic model, the stochastic relative immune clearance rates $h_{i}=\frac{d_{i}}{e_{i}}+\frac{\tau_{i}^{2}}{2e_{i}}$ classify overall dynamics into three cases, the system with only innate immune response, the system with only adaptive immune response, and the system with both innate and immune responses. Because *h*_*i*_ is a sum of two positive terms, one is the relative immune clearance rate and one contains uncertainty variances, this provides some ranges for estimated parameter values of immune clearance rate *c*_*i*_ and immune stimulation rates *s*_*i*_. In this sense, the classification of overall dynamics of our deterministic model is stable or robust with respect to microenvironmental noises and uncertainties from immune responses.

In each case or stochastic sub-model, there is a quantity, called the infection value *θ*, which determines long-term outcomes of the model. This quantity is actually the infected tumor cell component of the immune-free equilibrium state in the deterministic model. For example, when the stochastic relative immune clearance rates dominate *θ* in three cases with only innate immune response, with only adaptive immune response, and with both innate and adaptive immune responses, the immune-free ergodic invariant probability measure in each case is globally attractive in its domain; when the stochastic relative immune clearance rates are below *θ* in each of these three cases, each of three sub-models shows persistence in the sense that all cell populations never die out. We see that this quantity of the infection value *θ* is universal in the sense that it serves as a critical value to classify asymptotical behaviors of two stochastic sub-models and full model. Therefore, this quantity should indicate some intrinsic property of oncolytic viral therapy. The medical implications may be that, we should make the infection value *θ* as small as possible, so we can reduce the total tumor burden although we cannot completely eradicate the tumor. A simple analysis of the infection value shows that it is related to viral burst size [26]. Viral burst sizes can be genetically changed [5]. Hence, changing the infection value is medically feasible. This is one aspect that the stochastic model can provide more insights and medical implications.

Our stochastic model also displays some new mathematical features. One is what we called stochastic Hopf bifurcation without parameters. As we mentioned in part I, there is no common physical mechanism for Hopf bifurcation without parameters in deterministic systems. In cancer viral therapy, we might regard immune cells as predators where innate immune cells prey on infected tumor cells and adaptive immune cells prey on tumor cells. We may also consider infected tumor cells to be predators who prey on tumor cells. It is well known that there exist periodic solutions or interactions in predator-prey systems when the system parameters satisfy some conditions. We also know that immune clearance rates are not fixed constants and they change according to on-site immune cell density and cellular signals [8,21]. This gives some possibilities for parameter changes in viral therapy. From our stochastic model, it seems to be easier to explain why viral therapy has many outcomes. Both innate and adaptive immune cells may have different clearance rates and stimulation rates and, furthermore, they are subject to influences from microenvironmental noises and uncertainties. Thus, stochastic periodic solutions appear when these rates change. This may provide some explanations for occurrences of Hopf bifurcations without parameters in both deterministic and stochastic models.

It is obvious that there are many microenvironmental noises and uncertainties in cancer virotherapy such as uncertainties related to tumor cells, uncertainties related to interactions between tumor cells and viruses, etc. These require further studies. We will consider them in the future.

## Supplementary Material

supp-material

## Appendix A

We review some technical concepts and results in the theory of Markov processes, extracted from [4,10, 18–20], that are used in Section 3.

### A.1. Hypoellipticity and Hörmander’s condition

Let *X*(*t*) = (*X*_1_(*t*), …, *X*_*n*_(*t*))^*T*^ be the solution to the Stratonovich SDE system

(31)
dX=f(X)dt+g(X)∘dW

where *f*(*x*) = *f*(*x*), …, *fn*(*x*))^*T*^ and time *gt*(*x*) = (*g*_*ij*_(*x*))_*n*×*m*_ are *C*^∞^ and such that (31) has global solutions (i.e. solutions of (31) exist for all time *t* ≥0). Notice that *W* = (*W*_1_, …, *W*_*m*_)^*T*^ is a standard *m*-dimensional Brownian Motion. We equip (31) with a complete filtered probability space $\left(\Omega ,ℱ,{\left\{{ℱ}_{t}\right\}}_{t\geq 0},\mathbb{P}\right)$. The transition probability function of the solution *X*(*t*) to the system (31)

$$P(t,x,\cdot )=\mathbb{P}\left\{X(t)\in \cdot |X(0)=x\right\}≕{\mathbb{P}}_{x}\left\{X(t)\in \cdot \right\}$$

induces a family of operators *T*_*t*_ given by

$$T_{t}f(x)=\underset{{\mathbb{R}}^{n}}{\int }P(t,x,dy)f(y)=E_{x}[f(X(t))]$$

Denote by $C_{b}\left({\mathbb{R}}^{n}\right)$ the space of bounded continuous functions on ${\mathbb{R}}^{n}$. If the operator *T*_*t*_ maps $C_{b}\left({\mathbb{R}}^{n}\right)$ to $C_{b}\left({\mathbb{R}}^{n}\right)$ then the transition probability function *P*(*t*, *x*, ·) is called *Feller*. Then we say that the solution *X*(*t*) has the Feller property. If *T*_*t*_ maps the space of bounded measurable functions on ${\mathbb{R}}^{n}$ to $C_{b}\left({\mathbb{R}}^{n}\right)$ then we say that the solution *X*(*t*) has the strong Feller property.

By Chapman-Kolmogorov’s equation, *T*_*t*+*s*_ = *T*_*t*_*T*_*s*_ for all *t* > 0 and *s* > 0. Hence *T*_*t*_ is a semigroup on $C_{b}\left({\mathbb{R}}^{n}\right)$. The generator of the semigroup *T*_*t*_ is given, on sufficiently smooth functions, by the 2^nd^-order differential operator

$$ℒV(x)=\underset{i}{\sum }f_{i}(x)\frac{\partial V(x)}{\partial x_{i}}+\frac{1}{2}\underset{i,j}{\sum }a_{ij}(x)\frac{\partial^{2}V(x)}{\partial x_{i}\partial x_{j}}=f(x)\cdot V_{x}+\frac{1}{2}\text{trace}\left(g(x)V_{xx}g{\left(x\right)}^{T}\right)$$

where *a*_*ij*_(*x*) = (*g*(*x*)*g*(*x*)^*T*^)_*ij*_. The adjoint operator ℒ* of ℒ is given by

$${ℒ}^{*}V(x)=-\underset{i}{\sum }\frac{\partial }{\partial x_{i}}\left(V(x)f_{i}(x)\right)+\frac{1}{2}\underset{i,j}{\sum }\frac{\partial^{2}}{\partial x_{i}\partial x_{j}}\left(V(x)a_{ij}(x)\right)$$

which is called the Fokker-Planck operator. Now we write

(32)
P(t,x,dy)=p(t,x,y)dy

Even though, in general, *P*(*t*, *x*, *dy*) does not necessarily have a density with respect to the Lebesgue measure, (32) can be understood in the sense of distribution. The density *p*(*t*, *x*, *y*) in (32) satisfies the Fokker-Planck equation

(33)
$$\frac{\partial }{\partial t}p(t,\cdot ,y)={ℒ}^{*}p(t,\cdot ,y).$$

We say that the system (31) is *non-degenerate* if the generator ℒ corresponding to (31) is *elliptic*, that is, the matrix *A*(*x*) = (*a*_*ij*_(*x*)) is positive definite. It is well-known that if ℒ is elliptic and hence ℒ* is also elliptic, then the equation (33) has a smooth solution. In other words, *P*(*t*, *x*, ·) has *C*^∞^ density *p*(*t*, *x*, *y*).

However, in many interesting physical applications, the generator fails to be elliptic. There is a theorem due to Hörmander which gives a useful criterion to obtain the regularity of *p*(*t*, *x*, *y*). Note that the system (31) can be written as

$$dX=g_{0}(X)dt+\overset{m}{\underset{i=1}{\sum }}g_{i}(x)\circ dW_{i}$$

where *g*_0_(*x*) = *f*(*x*) and *g*_*i*_(*x*) is the *i*th column of the matrix *g*(*x*). We say that the solution *X*(*t*) to the system (31) is said to satisfy *Hörmander’s condition* [10] if the set of vector fields

$${\left\{g_{i}\right\}}_{i=1}^{m},{\left\{\left[g_{i},g_{j}\right]\right\}}_{i,j=0}^{m},{\left\{\left[g_{i},\left[g_{j},g_{k}\right]\right]\right\}}_{i,j,k=0}^{m},\ldots$$

spans ${\mathbb{R}}^{n}$ at every point *x* in which the operator [·, ·] is the Lie Bracket defined as follows: if *A* = (*A*_1_(*x*), …, *A*_*n*_(*x*))^*T*^ and *B* = (*B*_1_(*x*), …, *B*_*n*_(*x*))^*T*^ are 2 vector fields in ${\mathbb{R}}^{n}$ then

$$[A,B]={\left({\left[A,B\right]}_{1},\ldots ,{\left[A,B\right]}_{n}\right)}^{T}\text{ where }{\left[A,B\right]}_{j}=\overset{n}{\underset{k=1}{\sum }}\left(A_{k}\frac{\partial B_{j}}{\partial x_{k}}-B_{k}\frac{\partial A_{j}}{\partial x_{k}}\right)$$

for *j* = 1, …, *n*. Then the generator corresponding to the system (31) that has solution satisfying Hörmander’s condition is called *hypoelliptic*. The following theorem (see [25], [4], and [20]) is used in the proof of Theorem 2.1.

**Theorem 1** (*A version of Hörmander Theorem*). *If the generator of the solutions X*(*t*) *to the system* (31) *is hypoelliptic* (*i.e*. *the solutions X*(*t*) *satisfies Hörmander’s condition*) *then the transition probability P*(*t*, *x*, ·) *of X*(*t*) *has density p*(*t*, *x*, *y*) *which is C*^∞^ *function of* (*t*, *x*, *y*) *and the semigroup T*_*t*_ *is strong Feller*.

### A.2. Control theory and support theorem

Let *X*(*t*) = (*X*_1_(*t*), …, *X*_*n*_(*t*))^*T*^ be the solution to the Ito SDE system

(34)
dX=f(X)dt+g(X)dW,X(0)=x,

where *f*(*x*) = (*f*_1_(*x*), …, *f*_*n*_(*x*))^*T*^ and *g*(*x*) = (*g*_1_(*x*), …, *g*_*n*_(*x*))^*T*^ with *g*_*i*_(*x*) = (*g*_*i*1_(*x*), …, *g*_*im*_(*x*)) are such that the system (34) has global solutions. *W* = (*W*_1_, …, *W*_*m*_)^*T*^ is a standard *m*-dimensional Brownian Motion. To study the ergodic property of *X*(*t*), we need to establish which sets can be reached by *X*(*t*) from a point *x* in time *t*, that is, determine when *P*(*t*, *x*, *A*) > 0 for some Borel set *A* in ${\mathbb{R}}^{n}$. Now replace the Brownian Motion *W* = *W*(*t*) by a piecewise polygonal approximation

$$W_{t}^{\left(N\right)}=W\left(\frac{k}{N}\right)+N\left(t-\frac{k}{N}\right)\left(W\left(\frac{k+1}{N}\right)-W\left(\frac{k}{N}\right)\right),\;\frac{k}{N}\leq t\leq \frac{k+1}{N}.$$

Then its derivative $\frac{dW_{t}^{\left(N\right)}}{dt}$ is piecewise constant. It can be shown that the solutions *X*^(*N*)^ = *X*^*(N)*^(*t*) to the Ito SDE system

(35)
$$dX^{\left(N\right)}=f\left(X^{\left(N\right)}\right)dt+g\left(X^{\left(N\right)}\right)dW_{t}^{\left(N\right)},\;X^{\left(N\right)}(0)=x,$$

converge a.s. to $\bar{X}$ uniformly on any compact interval [*t*_1_, *t*_2_] where $\bar{X}=\bar{X}(t)$ is the solution to the Ito SDE system

(36)
$$dX=\left[f(X)+\frac{1}{2}c(X)\right]dt+g(X)dW$$

where *c*(*X*) = (*c*_1_(*X*), …, *c*_*n*_(*X*))^*T*^ and $c_{i}(X)=\sum_{j=1}^{n}\frac{\partial g_{i}(X)}{\partial X_{j}}g_{j}{\left(X\right)}^{T}(i=1,\ldots ,n)$. The system (36) is equivalent to the Stratonovich SDE system

(37)
dX=f(X)dt+g(X)∘dW.

Since any piecewise continuous function can be approximated by a sequence of piecewise constant functions, the system (35) can be approximated by the following ODE system when *N* is large enough

(38)
$$\dot{X}=f(X)+g(X)u$$

in which *u* = *u*(*t*) = (*u*_1_(*t*), …, *u*_*m*_(*t*))^*T*^ is a piecewise continuous on ${\mathbb{R}}_{+}$ and takes values in ${\mathbb{R}}^{m}$. This is an ordinary non-autonomous differential equation system. The function *u* is called a *control* and the system (38) a *control system* corresponding to the system (37). So we can derive several properties of the system (37) by studying the control system (38).

Denote by ${𝒮}_{x}^{\left[0,t\right]}$ the support of the diffusion *X*(*t*) of the system (37), i.e., ${𝒮}_{x}^{\left[0,t\right]}$ is the smallest closed (in the uniform topology) subset of $\left\{h\in 𝒞\left([0,t],{\mathbb{R}}^{n}\right):h(0)=x\right\}$ such that

$$\mathbb{P}\left\{X(s)\in {𝒮}_{x}^{\left[0,t\right]}\forall s\in [0,t]\right\}=1.$$

Next, denote by 𝒰 the set of all piecewise continuous functions *u*. We say that a point *y* is accessible from *x* in time *t* if there exists a control *u* ∈ 𝒰 such that the solution *X*(*t*, *u*) to the system (38) satisfies *X*(0, *u*) = *x* and *X*(*t*, *u*) = *y*. Let *A*_*t*_(*x*) be the set of all points $y\in {\mathbb{R}}^{n}$ such that *y* is accessible from *x* in time *t* and ${𝒞}_{x}^{\left[0,t\right]}(𝒰)$ the set of all solutions *X*(*t*, *u*) to the system (38) starting at *x* with control *u* ∈ 𝒰. It is clear that ${𝒞}_{x}^{\left[0,t\right]}(𝒰)$ is a subset of $\left\{h\in 𝒞\left([0,t],{\mathbb{R}}^{n}\right):h(0)=x\right\}$. The following theorem, generally called the support theorem (see [4] and [12]), helps connect the properties of solutions to the Stratonovich SDE system (37) and solutions to the corresponding control system (38).

**Theorem 2** (*Stroock-Varadhan support theorem*).

$${𝒮}_{x}^{\left[0,t\right]}=\bar{{𝒞}_{x}^{\left[0,t\right]}(𝒰)}$$

*where the bar indicates the closure in the uniform topology*.

As a consequence, if we denote by supp *μ* the support of a measure *μ* on ${\mathbb{R}}^{n}$ then we obtain

$$\text{supp }P(t,x,\cdot )=\bar{A_{t}(x)}.$$

### A.3. Exponential ergodicity

Let *X* be a locally compact separable metric space, and ℬ(*X*) the Borel *σ*-algebra on *X*. Let Φ = {Φ_*t*_: *t* ≥ 0} be a non-explosive homogeneous Markov process with state space (*X*, ℬ(*X*)) and *P*(*t*, *x*, ·) its transition probability. Consider the process Φ on a probability space $\left(\Omega ,ℱ,{\left\{{\mathbb{P}}_{x}\right\}}_{x\in X}\right)$ where the probability measure ${\mathbb{P}}_{x}$ satisfies ${\mathbb{P}}_{x}\left\{\Phi_{t}\in A\right\}=P(t,x,A)$ for all *x* ∈ *X*, *t* ≥ 0, and *A* ∈ ℬ(*X*). Suppose further that Φ is a Feller process. For a probability measure *a* on ${\mathbb{R}}_{+}$, define a sampled Markov transition function *K*_*a*_ of Φ by

$$K_{a}(x,B)=\overset{\infty }{\underset{0}{\int }}P(t,x,B)a(dt).$$

*K*_*a*_ is said to possess *an everywhere nontrivial continuous component* if there is a kernel $T:(X,ℬ(X))\to {\mathbb{R}}_{+}$ satisfying

- For each *B* ∈ ℬ(*X*) fixed, the function *T*(·, *B*) is lower semi-continuous, that is, for all *x* ∈ *X*
  $$\underset{y\to x}{\text{lim inf}}\;T(y,B)\geq T(x,B).$$
- For each *x* ∈ *X* fixed, *T*(*x*, ·) is a nontrivial measure (i.e. *T*(*x*, *X*) > 0) satisfying *K*_*a*_(*x*, *B*) ≥ *T*(*x*, *B*) for all *B* ∈ ℬ(*X*).

The process Φ is called a *T-process* if for some probability measure *a*, *K*_*a*_ admits an everywhere nontrivial continuous component. A subset *A* ∈ ℬ(*X*) is said to be *petite* for the *δ*-skeleton $\left\{\Phi_{n\delta }:n\in \mathbb{N}\right\}$ of Φ if there exists a probability measure *b* on ℕ and a nontrivial measure *ψ*(·) on *X* such that for all *x* ∈ *A* and *B* ∈ ℬ(*X*)

$$K_{b}(x,B)≔\overset{\infty }{\underset{n=1}{\sum }}P(n\delta ,x,B)b(n)\geq \psi (B).$$

The process Φ is called *bounded in probability on average* if for all *x* ∈ *X* and *ϵ* > 0, there is a compact set *C*_*ϵ*,*x*_ satisfying

$$\underset{t\to \infty }{\text{lim inf}}\;\frac{1}{t}\overset{t}{\underset{0}{\int }}P\left(s,x,C_{\epsilon ,x}\right)ds\geq 1-\epsilon .$$

We say that Φ is *ergodic* with respect to an invariant probability measure *π* if the transition probability *P*(*t*, *x*, ·) of Φ converges to *π* in total variation norm, that is,

$$∥P(t,x,\cdot )-\pi (\cdot ){∥}_{TV}\to 0\text{ as }t\to \infty \text{. }$$

We say that Φ is *exponentially ergodic* with respect to an invariant probability measure *π* if there exist a constant *β* ∈ (0, 1) and a finite-valued function $B(x):X\to {\mathbb{R}}_{+}$ such that for all *t* > 0 and *x* ∈ *X*

$$∥P(t,x,\cdot )-\pi (\cdot ){∥}_{TV}\leq B(x)\beta^{t}$$

where ∥·∥_*TV*_ is the total variation norm on *X*.

Finally, we state two theorems that are used in the proof of Theorem 3.1.

**Theorem 3** (*see Theorem 8.1 in* [18]). *Suppose that* Φ *is bounded in probability on average and* Φ *is a T-process*. *If π is an invariant probability measure for* Φ *then for any π-integrable function f and x* ∈ *X we have*

$${\mathbb{P}}_{x}\left\{\underset{t\to \infty }{\text{lim}}\frac{1}{t}\overset{t}{\underset{0}{\int }}f\left(\Phi_{s}\right)ds=\underset{X}{\int }fd\pi \right\}=1.$$

**Theorem 4** (*A criterion for exponential ergodicity*, *see Theorem 6.1 in* [19]). *Suppose that all compact sets are petite for some skeleton chain and there exists a positive norm-like function* $V(x):X\to {\mathbb{R}}_{+}$ *such that* ℒ*V* (*x*) ≤ −*cV* (*x*) + *d for all x* ∈ *X and for some constants c* > 0, *d* < ∞. *Then there is a unique invariant probability measure π and* Φ *is exponentially ergodic with respect to π*.
