# Supplementary material for: Hopf bifurcation without parameters in deterministic and stochastic modeling of cancer virotherapy, part II
Source: J Math Anal Appl. Author manuscript; Available in PMC 2023 Mar 30. (PMC10062412; doi:10.1016/j.jmaa.2022.126444)
Supplement: supp-material [file NIHMS1883942-supplement-supp-material.pdf]

Supplementary Material for Hopf bifurcation without parameters in deterministic and stochastic modeling of cancer virotherapy, part II

Tuan Anh Phan[1] Jianjun Paul Tian[2]

[1] Institute for Modeling Collaboration and Innovation, The University of Idaho
Moscow, Idaho 83844, USA

[2] Department of Mathematical Sciences, New Mexico State University
Las Cruces, New Mexico 88001, USA

E-mail addresses: tphan@uidaho.edu, jtian@nmsu.edu

In this supplementary material, we illustrate in detail the algorithms and MATLAB codes that are used to compute numerically the solution paths of the 4-dimensional stochastic cancer virotherapy model as well as to demonstrate the occurrence of Hopf bifurcation without parameters by computing numerically the Lyapunov exponents of all solution components. The material is organized into 3 parts: (1) We derive a general class of stochastic Runge-Kutta methods of strong order 1.0 and a particular case from this class that we used to solve numerically our 4-dim virotherapy model; (2) We present the algorithm of stochastic Runge-Kutta method of strong order 1 to approximate the solution paths of our model and the algorithm of simulating the Lyapunov exponents of the solution components; (3) We provide in detail the MATLAB codes for two above algorithms.

1 Strong stochastic Runge-Kutta scheme of order 1

First of all, we present briefly the Ito's formula for a system of SDEs with the usage of differential operators. By this way, we can easily keep track of the stochastic Taylor expansion of an SDE system as we apply the Ito's formula for the drift term and the diffusion term of the SDE system.

1.1 Ito's formula for a SDE system

Let $X(t) = (X^1(t), \cdots, X^d(t))^T$ solve the d-dimensional SDE system with m independent Brownian Motions

$$
\begin{aligned}
dX^1(t) &= g^{1,0}(t, X^1(t), \cdots, X^d(t))dt + \sum_{k=1}^{m} g^{1,k}(t, X^1(t), \cdots, X^d(t))dW^k(t),\\
&\vdots \\
dX^d(t) &= g^{d,0}(t, X^1(t), \cdots, X^d(t))dt + \sum_{k=1}^{m} g^{1,k}(t, X^1(t), \cdots, X^d(t))dW^k(t).
\end{aligned} \tag{1}
$$

An equivalent shorthand vector form of (1) is

$$dX(t) = g^0(t, X(t))dt + \sum_{k=1}^{m} g^k(t, X(t))dW^k(t), \tag{2}$$

where each $g^k : [0,T] \times \mathbb{R}^d \to \mathbb{R}^d$, $k = 0, 1, \cdots, m$, has the form $g^k = (g^{1,k}, \cdots, g^{d,k})^T$. We assume that all the functions g^k are smooth enough so that the solution $X(t)$ to the system (1) exists and is unique. Let $U : [0,T] \times \mathbb{R}^d \to \mathbb{R}$ be a scalar-valued function with continuous partial derivatives

$$\frac{\partial U}{\partial t}, \quad \frac{\partial U}{\partial x_j}, \quad \frac{\partial^2 U}{\partial x_i \partial x_j} \quad \text{for } i, j = 1, \cdots, d.$$

Then the scalar process $Y(t) := U(t, X(t))$ satisfies the following Ito's formula in differential form

$$\begin{aligned} dY(t) = &\left\{ \frac{\partial U}{\partial t} + \sum_{j=1}^{d} g^{j,0} \frac{\partial U}{\partial x_j} + \frac{1}{2} \sum_{k=1}^{m} \sum_{i,j=1}^{d} g^{j,k} g^{i,k} \frac{\partial^2 U}{\partial x_i \partial x_j} \right\} dt \\ &+ \sum_{k=1}^{m} \sum_{i=1}^{d} g^{i,k} \frac{\partial U}{\partial x_i} dW^k(t), \end{aligned} \tag{3}$$

where all the coefficient functions and partial derivatives are evaluated at $(t, X(t))$. The Ito's formula (3) can be written in the compact form

$$dY(t) = \mathbf{L}^0 U(t, X(t))dt + \sum_{k=1}^{m} \mathbf{L}^k U(t, X(t))dW^k(t) \tag{4}$$

using the differential operators

$$\mathbf{L}^0 := \frac{\partial}{\partial t} + \sum_{j=1}^{d} g^{j,0} \frac{\partial}{\partial x_j} + \frac{1}{2} \sum_{k=1}^{m} \sum_{i,j=1}^{d} g^{j,k} g^{i,k} \frac{\partial^2}{\partial x_i \partial x_j}$$

and

$$\mathbf{L}^k := \sum_{i=1}^{d} g^{i,k} \frac{\partial}{\partial x_i} \qquad k = 1, \cdots, m.$$

The integral form of the system (2) can be expressed as

$$X(t) = X(t_0) + \int_{t_0}^{t} g^0(\tau, X(\tau))d\tau + \sum_{k=1}^{m} \int_{t_0}^{t} g^k(\tau, X(\tau))dW^k(\tau). \tag{5}$$

Now, for each $k = 0, 1, \cdots, m$, applying the Ito's formula to the function $g^k(\tau, X(\tau))$ gives

$$g^k(\tau, X(\tau)) = g^k(t_0, X(t_0)) + \int_{t_0}^{\tau} \mathbf{L}^0 g^k(s, X(s))ds + \sum_{v=1}^{m} \int_{t_0}^{\tau} \mathbf{L}^v g^k(s, X(s))dW^v(s). \tag{6}$$

By (5) and (6), it follows that

$$\begin{aligned} X(t) = &X(t_0) + g^0(t_0, X(t_0))(t - t_0) + \sum_{v=1}^{m} g^v(t_0, X(t_0))(W^v(t) - W^v(t_0)) \\ &+ \int_{t_0}^{t} \int_{t_0}^{\tau} \mathbf{L}^0 g^0(s, X(s))dsd\tau + \sum_{v=1}^{m} \int_{t_0}^{t} \int_{t_0}^{\tau} \mathbf{L}^v g^0(s, X(s))dW^v(s)d\tau \\ &+ \sum_{k=1}^{m} \int_{t_0}^{t} \int_{t_0}^{\tau} \mathbf{L}^0 g^k(s, X(s))dsdW^k(\tau) + \sum_{k=1}^{m} \sum_{v=1}^{m} \int_{t_0}^{t} \int_{t_0}^{\tau} \mathbf{L}^v g^k(s, X(s))dW^v(s)dW^k(\tau) \end{aligned} \tag{7}$$

Then (7) is called the stochastic Taylor expansion of the solution $X(t)$ to the system (5).

1.2 Euler-Maruyama for a SDE system

If we keep the first 3 terms and discard four double integrals in (7) then we obtain the Euler-Maruyama (EM) method for the SDE system (2) given by

$$X_{n+1} = X_n + \Delta t\, g^0(t_n, X_n) + \sum_{k=1}^{m} g^k(t_n, X_n)\Delta W_n^k \tag{8}$$

where the Brownian increments ΔW_n^k $(k = 1, \cdots, m)$ are independent $\sqrt{\Delta t}\, N(0,1)$ distributed random variables. The EM method (8) has strong convergence order 1/2 and weak convergence order 1 (see Chapter 8 in [1] for the definition of strong and weak convergence). When the SDE system has additive noise, i.e. all the g^k are independent of the state variables, the strong convergence order of the EM method increases to 1.

1.3 Milstein for a SDE system

If we continue to apply the Ito's formula for the integrand of the last double integral in (7) then we obtain the following expansion

$$\begin{aligned} X_{n+1} = X_n &+ \Delta t\, g^0(t_n, X_n) + \sum_{k=1}^{m} g^k(t_n, X_n)\Delta W_n^k \\ &+ \sum_{k=1}^{m}\sum_{v=1}^{m} \mathbf{L}^v g^k(t_n, X_n) \int_{t_n}^{t_{n+1}} \int_{t_n}^{\tau} dW^v(s)\, dW^k(\tau) + R_1(t_{n+1}, t_n) \end{aligned} \tag{9}$$

in which the remainder $R_1(t_{n+1}, t_n)$ includes 3 double integrals and 2 triple integrals that are not written here due to their complexity. Then, discarding this remainder in (9), we obtain the so-called Milstein method for the SDE system (2) given by

$$\begin{aligned} X_{n+1} = X_n &+ \Delta t\, g^0(t_n, X_n) + \sum_{k=1}^{m} g^k(t_n, X_n)\Delta W_n^k \\ &+ \sum_{k=1}^{m}\sum_{v=1}^{m} \mathbf{L}^v g^k(t_n, X_n) \int_{t_n}^{t_{n+1}} \int_{t_n}^{\tau} dW^v(s)\, dW^k(\tau) \end{aligned} \tag{10}$$

The Milstein method (10) has strong order 1 and weak order 1. Next, let's consider the stochastic double integrals in (10)

$$I_{(v,k),n} := \int_{t_n}^{t_{n+1}} \int_{t_n}^{\tau} dW^v(s)\, dW^k(\tau). \tag{11}$$

If $v = k$ then we can easily compute $I_{(k,k),n} = \frac{1}{2}\left[(\Delta W_n^k)^2 - \Delta t\right]$. Then (10) can be written as

$$\begin{aligned} X_{n+1} = X_n &+ \Delta t\, g^0(t_n, X_n) + \sum_{k=1}^{m} g^k(t_n, X_n)\Delta W_n^k \\ &+ \frac{1}{2}\sum_{k=1}^{m} \mathbf{L}^k g^k(t_n, X_n) \left[(\Delta W_n^k)^2 - \Delta t\right] + \sum_{\substack{k,v=1 \\ k\neq v}}^{m} \mathbf{L}^v g^k(t_n, X_n) I_{(v,k),n} \end{aligned} \tag{12}$$

If the SDE system (2) has commutative noises, i.e.

$$\mathbf{L}^v g^k(t,x) \equiv \mathbf{L}^k g^v(t,x) \quad \text{for } 1 \le k \ne v \le m$$

then it is easy to show that for $k \ne v$

$$I_{(v,k),n} + I_{(k,v),n} = \Delta W_n^v \Delta W_n^k$$

and hence the Milstein method (12) reads

$$\begin{aligned} X_{n+1} = X_n + \Delta t\, g^0(t_n, X_n) + \sum_{k=1}^m g^k(t_n, X_n)\Delta W_n^k \\ + \frac{1}{2}\sum_{k=1}^m \mathbf{L}^k g^k(t_n, X_n)\left[(\Delta W_n^k)^2 - \Delta t\right] + \frac{1}{2}\sum_{\substack{k,v=1\\k\ne v}}^m \mathbf{L}^v g^k(t_n, X_n)\Delta W_n^v\,\Delta W_n^k. \end{aligned} \tag{13}$$

In the noncommutative case, the stochastic double integral $I_{(v,k),n}$ can be approximated numerically by EM method. Without loss of generality, suppose we wish to approximate

$$I_{(2,1),n} = \int_{t_n}^{t_{n+1}} \int_{t_n}^{\tau} dW^2(s)\, dW^1(\tau).$$

It is not difficult to see that the system of SDEs

$$\begin{aligned} dY^1(u) &= Y^2(u) dW^1(u) \\ dY^2(u) &= dW^2(u) \end{aligned} \tag{14}$$

with $u \in [t_n, t_{n+1}]$ and initial condition $Y^1(t_n) = Y^2(t_n) = 0$ has a solution for which $Y^1(t_{n+1}) = I_{(2,1),n}$. We apply the EM method to the system (14) with a suitably small timestep δt. Write $\delta t = \dfrac{t_{n+1} - t_n}{K}$, we let $t'_k = t_n + k\delta t$ be a general subgrid point with $\delta W_{n,k}^j := W^j(t'_{k+1}) - W^j(t'_k)$ $(j = 1, 2)$ denoting the corresponding Brownian increments. Then the EM method for the system (14) takes the form

$$\begin{aligned} Y^1(0) = Y^2(0) = 0 \text{ and} \\ Y_{k+1}^1 = Y_k^1 + Y_k^2\, \delta W_{n,k}^1 \\ Y_{k+1}^2 = Y_k^2 + \delta W_{n,k}^2 \end{aligned} \tag{15}$$

for $0 \le k \le K - 1$. The order 1/2 strong convergence rate of the EM method (15) ensures

$$\mathbb{E}\left(|Y_k^1 - I_{(2,1),n}|\right) \le C\sqrt{\delta t}$$

for small δt and a suitable constant C. Hence we can approximate $I_{(2,1),n}$ in the Milstein method (12) by Y_k^1 with $\delta t \approx (\Delta t)^2$ without affecting the overall strong convergence rate of 1.

1.4 Runge-Kutta for a SDE system

To utilize the Milstein method (10) for the SDE system (2), we have to compute all the partial derivatives $\mathbf{L}^v g^k$ $(k, v = 1, \cdots, m)$ and all the iterated and cross-term Ito's integrals $I_{(v,k),n}$. This makes the computation of the formulation (10) extremely expensive. Rößler (2010) derived a general class of stochastic Runge-Kutter schemes of strong order 1 (see Section 6.2 in [2]), in which

computing the partial derivatives $\mathbf{L}^v g^k$ and the Ito's integrals $I_{(v,k),n}$ are avoided and these Ito's integrals only appear in the supporting values. The general schemes are as follows.

Start from $X_0 = X(0)$ and divide the integration interval $[0,T]$ into N steps $t_0 < t_1 < t_2 < \cdots < t_N = T$ such that $\Delta t = t_{n+1} - t_n = \frac{T}{N}$ for all $n = 0, 1, \cdots, N-1$. The integration method is characterized by its extended Butcher tableau

$$
\begin{array}{c|c|c|c}
\mathbf{c}^{(0)} & \mathbf{A}^{(0)} & \mathbf{B}^{(0)} & \\
\hline
\mathbf{c}^{(1)} & \mathbf{A}^{(1)} & \mathbf{B}^{(1)} & \\
\hline
 & \boldsymbol{\alpha}^T & [\boldsymbol{\gamma}^{(1)}]^T & [\boldsymbol{\gamma}^{(2)}]^T
\end{array}
$$

which, on each step n, approximate the solution as follows:

$$
\begin{aligned}
X_{n+1} = X_n &+ \sum_{i=1}^{s} \alpha_i \, g^0 \left(t_n + c_i^{(0)} \Delta t, \tilde{X}_i^{(0)}\right) \Delta t \\
&+ \sum_{i=1}^{s} \sum_{k=1}^{m} \left(\gamma_i^{(1)} \Delta W_n^k + \gamma_i^{(2)} \sqrt{\Delta t}\right) g^k \left(t_n + c_i^{(1)} \Delta t, \tilde{X}_i^{(k)}\right)
\end{aligned} \tag{16}
$$

with the supporting values

$$
\tilde{X}_i^{(0)} = X_n + \sum_{j=1}^{s} A_{ij}^{(0)} g^0 \left(t_n + c_j^{(0)} \Delta t, \tilde{X}_j^{(0)}\right) \Delta t + \sum_{j=1}^{s} \sum_{l=1}^{m} B_{ij}^{(0)} g^l \left(t_n + c_j^{(1)} \Delta t, \tilde{X}_j^{(l)}\right) \Delta W_n^l, \tag{17}
$$

$$
\tilde{X}_i^{(k)} = X_n + \sum_{j=1}^{s} A_{ij}^{(1)} g^0 \left(t_n + c_j^{(0)} \Delta t, \tilde{X}_j^{(0)}\right) \Delta t + \sum_{j=1}^{s} \sum_{l=1}^{m} B_{ij}^{(1)} g^l \left(t_n + c_j^{(1)} \Delta t, \tilde{X}_j^{(l)}\right) \frac{I_{(l,k),n}}{\sqrt{\Delta t}}, \tag{18}
$$

for $i = 1, \cdots, s$ and $k = 1, \cdots, m$. Recall that, in (17) and (18),

$$
\Delta W_n^l := \int_{t_n}^{t_{n+1}} dW^l(\tau) = W^l(t_{n+1}) - W^l(t_n), \tag{19}
$$

$$
I_{(l,k),n} := \int_{t_n}^{t_{n+1}} \int_{t_n}^{\tau_2} dW^l(\tau_1) dW^k(\tau_2). \tag{20}
$$

For our model simulation, we use a stochastic Runge-Kutta method with the following extended Butcher tableau

$$
\begin{array}{c|ccc|ccc|ccc}
0 & 0 & 0 & 0 & 0 & 0 & 0 & & & \\
1 & 1 & 0 & 0 & 0 & 0 & 0 & & & \\
0 & 0 & 0 & 0 & 0 & 0 & 0 & & & \\
\hline
0 & 0 & 0 & 0 & 0 & 0 & 0 & & & \\
1 & 1 & 0 & 0 & 1 & 0 & 0 & & & \\
1 & 1 & 0 & 0 & -1 & 0 & 0 & & & \\
\hline
 & \frac{1}{2} & \frac{1}{2} & 0 & 1 & 0 & 0 & 0 & \frac{1}{2} & -\frac{1}{2}
\end{array}
$$

which corresponds to the following iterative scheme

$$
\begin{aligned}
X_{n+1} = X_n &+ \frac{1}{2}\left[g^0(t_n, X_n) + g^0\left(t_n + \Delta t, \tilde{X}_2^{(0)}\right)\right] \Delta t \\
&+ \sum_{k=1}^{m} \left\{ \Delta W_n^k \, g^k(t_n, X_n) + \frac{1}{2}\sqrt{\Delta t}\left[g^k\left(t_n + \Delta t, \tilde{X}_2^{(k)}\right) - g^k\left(t_n + \Delta t, \tilde{X}_3^{(k)}\right)\right] \right\}
\end{aligned} \tag{21}
$$

with the supporting values

$$\begin{aligned}\tilde{X}_2^{(0)} &= X_n + g^0(t_n, X_n)\Delta t,\\ \tilde{X}_2^{(k)} &= \tilde{X}_2^{(0)} + \sum_{l=1}^{m} g^l(t_n, X_n)\frac{I_{(l,k),n}}{\sqrt{\Delta t}},\\ \tilde{X}_3^{(k)} &= \tilde{X}_2^{(0)} - \sum_{l=1}^{m} g^l(t_n, X_n)\frac{I_{(l,k),n}}{\sqrt{\Delta t}},\end{aligned} \tag{22}$$

for $k = 1, \cdots, m$.

2 Algorithms

2.1 Simulating the solution paths

Our 4-dim virotherapy model can be written in the vector form

$$dU = f(U)dt + g_1(U)dW_1 + g_2(U)dW_2,\ U(0) = U_0,\ t \in [0, T] \tag{23}$$

in which

$$U = \begin{bmatrix} x \\ y \\ z_1 \\ z_2 \end{bmatrix},\ f(U) = \begin{bmatrix} rx(1-x-y) - axy - l_2xz_2 \\ axy - l_1yz_1 - y \\ e_1yz_1 - d_1z_1 \\ e_2yz_2 - d_2z_2 \end{bmatrix},\ g_1(U) = \begin{bmatrix} 0 \\ 0 \\ \tau_1 z_1 \\ 0 \end{bmatrix},\ g_2(U) = \begin{bmatrix} 0 \\ 0 \\ 0 \\ \tau_2 z_2 \end{bmatrix},$$

and $(W_1, W_2)^T$ is a 2-dim standard Brownian Motion.

We use the stochastic Runge-Kutta method with the extended Butcher tableau corresponding to (21) and (22) to compute one single path of the system (23). The scheme is as follows: Start from $U_0 = U(0)$ and divide the time interval $[0, T]$ into N steps $0 = t_0 < t_1 < \cdots < t_N = T$ such that $\Delta t = t_{n+1} - t_n = \frac{T}{N}$ for all $n = 0, 1, \cdots, N-1$. Then the iterative method takes the form

$$\begin{aligned}U_{n+1} = U_n &+ \frac{1}{2}\left[f(U_n) + f\left(\tilde{U}_2^{(0)}\right)\right]\Delta t\\ &+ \Delta W_{1,n}\, g_1(U_n) + \frac{1}{2}\sqrt{\Delta t}\left[g_1\left(\tilde{U}_2^{(1)}\right) - g_1\left(\tilde{U}_3^{(1)}\right)\right]\\ &+ \Delta W_{2,n}\, g_2(U_n) + \frac{1}{2}\sqrt{\Delta t}\left[g_2\left(\tilde{U}_2^{(2)}\right) - g_2\left(\tilde{U}_3^{(2)}\right)\right]\end{aligned} \tag{24}$$

with the supporting values

$$\begin{aligned}\tilde{U}_2^{(0)} &= U_n + f(U_n)\Delta t,\\ \tilde{U}_2^{(1)} &= \tilde{U}_2^{(0)} + g_1(U_n)\frac{\Delta W_{(1,1),n}}{\sqrt{\Delta t}} + g_2(U_n)\frac{\Delta W_{(2,1),n}}{\sqrt{\Delta t}},\\ \tilde{U}_2^{(2)} &= \tilde{U}_2^{(0)} + g_1(U_n)\frac{\Delta W_{(1,2),n}}{\sqrt{\Delta t}} + g_2(U_n)\frac{\Delta W_{(2,2),n}}{\sqrt{\Delta t}},\\ \tilde{U}_3^{(1)} &= \tilde{U}_2^{(0)} - g_1(U_n)\frac{\Delta W_{(1,1),n}}{\sqrt{\Delta t}} - g_2(U_n)\frac{\Delta W_{(2,1),n}}{\sqrt{\Delta t}},\\ \tilde{U}_3^{(2)} &= \tilde{U}_2^{(0)} - g_1(U_n)\frac{\Delta W_{(1,2),n}}{\sqrt{\Delta t}} - g_2(U_n)\frac{\Delta W_{(2,2),n}}{\sqrt{\Delta t}}.\end{aligned}$$

Note that

$$\begin{aligned}
\Delta W_{(1,1),n} &= \frac{1}{2}\left([\Delta W_{1,n}]^2 - \Delta t\right),\\
\Delta W_{(2,1),n} &= \int_{t_n}^{t_{n+1}}\int_{t_n}^{\tau_2} dW_2(\tau_1)dW_1(\tau_2),\\
\Delta W_{(1,2),n} &= \int_{t_n}^{t_{n+1}}\int_{t_n}^{\tau_2} dW_1(\tau_1)dW_2(\tau_2),\\
\Delta W_{(2,2),n} &= \frac{1}{2}\left([\Delta W_{2,n}]^2 - \Delta t\right).
\end{aligned}$$

On each time subinterval $[t_n, t_{n+1}]$, we can approximate $\Delta W_{(2,1),n}$, $\Delta W_{2,n}$, $\Delta W_{(1,2),n}$, and $\Delta W_{1,n}$ simultaneously by EM method. Indeed, let

$$\begin{aligned}
Y_2(u) &= W_2(u) - W_2(t_n) \text{ and}\\
Y_1(u) &= \int_{t_n}^{u} [W_2(\tau_2) - W_2(t_n)]\, dW_1(\tau_2),\ u \in [t_n, t_{n+1}],
\end{aligned}$$

then $(Y_1(u), Y_2(u))^T$ is the solution to the system

$$\begin{aligned}
dY_1(u) &= Y_2(u)dW_1(u)\\
dY_2(u) &= dW_2(u)
\end{aligned}$$

with the initial value $Y_1(t_n) = Y_2(t_n) = 0$. It is clear that $Y_1(t_{n+1}) = \Delta W_{(2,1),n}$ and $Y_2(t_{n+1}) = \Delta W_{2,n}$. Next, let

$$\begin{aligned}
Z_2(u) &= W_1(u) - W_1(t_n) \text{ and}\\
Z_1(u) &= \int_{t_n}^{u} [W_1(\tau_2) - W_1(t_n)]\, dW_2(\tau_2),\ u \in [t_n, t_{n+1}],
\end{aligned}$$

then $(Z_1(u), Z_2(u))^T$ is the solution to the system

$$\begin{aligned}
dZ_1(u) &= Z_2(u)dW_2(u)\\
dZ_2(u) &= dW_1(u)
\end{aligned}$$

with the initial value $Z_1(t_n) = Z_2(t_n) = 0$. It is clear that $Z_1(t_{n+1}) = \Delta W_{(1,2),n}$ and $Z_2(t_{n+1}) = \Delta W_{1,n}$. Now let $\delta t = \dfrac{t_{n+1} - t_n}{K}$ and $t'_m = t_n + m\delta t$ with $m = 0, 1, \cdots, K-1$. Denote $\delta W_j^{(n,m)} = W_j(t'_{m+1}) - W_j(t'_m)$ $(j = 1, 2)$. Then the EM method for simultaneously approximating the solutions of two above systems takes the form

$$\begin{aligned}
Y_1^0 &= Y_2^0 = 0,\ Z_1^0 = Z_2^0 = 0,\\
Y_1^{m+1} &= Y_1^m + Y_2^m\, \delta W_1^{(n,m)},\\
Y_2^{m+1} &= Y_2^m + \delta W_2^{(n,m)},\\
Z_1^{m+1} &= Z_1^m + Z_2^m\, \delta W_2^{(n,m)},\\
Z_2^{m+1} &= Z_2^m + \delta W_1^{(n,m)}.
\end{aligned}$$

Note that we can choose $\delta t = (\Delta t)^2$ and then $K = \dfrac{N}{T}$.

2.2 Simulating the Lyapunov exponents

Consider the system (23). Assume that $a > 1$ and $\frac{d_1}{e_1} + \frac{\tau_1^2}{2e_1} = \frac{d_2}{e_2} + \frac{\tau_2^2}{2e_2} =: h < 1$. Then $\lambda := \frac{r(a-1)}{a(a+r)} - h = 0$ iff either $a = a_1$ or $a = a_2$ in which $a_{1,2} = \frac{r}{2}\left(\frac{1}{h} - 1\right) \mp \frac{1}{2}\sqrt{r^2\left(\frac{1}{h} - 1\right)^2 - \frac{4r}{h}}$. We fix $a \in (a_1, a_2)$, then $\lambda > 0$. So, by Theorem 2.3, the system (23) has a collection of exponentially ergodic invariant probability measures $\{\Pi(k)\}_{k=0}^{\infty}$ where each $\Pi(k)$, $k \in (0, \infty)$, is supported by

$$S(k) = \left\{ \left(\frac{l_1 z_1 + 1}{a}, \frac{r(a-1-l_1 z_1)}{a(a+r)} - \frac{k l_2 z_1^{\rho}}{a+r}, z_1, k z_1^{\rho} \right) : z_1 \in \left(0, \frac{a-1}{l_1}\right) \right\}.$$

To demonstrate stochastic Hopf bifurcation without parameters, we numerically compute the Lyapunov exponents $\lambda_i(\Pi(k))$, $i = 1, 2, 3, 4$, of the corresponding solution components x, y, z_1, z_2 of the system (23) when k changes between 0 and ∞ to see if any of them could cross 0 or not. Note that, as k changes, the initial value $U_0(k) = (x_0, y_0, z_{10}, k z_{10}^{\rho})$ also changes. For each $k \in (0, \infty)$, when the solution of the system (23) starts close to the support $S(k)$, it will concentrate around this support for a long time. So for large $T > 0$

$$\lambda_1(\Pi(k)) \approx \frac{\log x(T)}{T}, \quad \lambda_2(\Pi(k)) \approx \frac{\log y(T)}{T}, \quad \lambda_3(\Pi(k)) \approx \frac{\log z_1(T)}{T}, \quad \text{and } \lambda_4(\Pi(k)) \approx \frac{\log z_2(T)}{T}.$$

3 MATLAB codes

Listings

1 Simulating the solution paths of 4d virotherapy model 8
2 Simulating the Lyapunov exponents of the solution components 11
3 Bivariate histogram function 14

Listing 1: Simulating the solution paths of 4d virotherapy model

```
function RK1_4d_viro
%% Stochastic Runge-Kutta method of strong order 1 for the 4-dim viro model
% dx = [r*x*(1 - x - y) - a*x*y - l2*x*z2]*dt
% dy = (a*x*y - l1*y*z1 - y)*dt
% dz1 = (e1*y*z1 - d1*z1)*dt + tau1*z1*dW1
% dz2 = (e2*y*z2 - d2*z2)*dt + tau2*z2*dW2
% with the initial condition [0.5; 0.5; 0.01; 0.01] and time t in [0,T]

%% parameters of the model
% r = 0.36;
% l1 = 0.48;
% l2 = 0.48;
% e1 = 10; d1 = 0.4;
% e2 = 10; d2 = 0.4;
% a = 5;
% tau1 = 0.01;
% tau2 = 0.01;

%****************************************************************
```

```matlab
%% Stochastic Runge-Kutta Algorithm
% initial value Uzero, the end time T
% the time step Dt for Runge-Kutta method,
% the time step dt for EM method
rng(250)
clf
Uzero = [0.5;0.5;0.01;0.01];
T = 2000;
Dt = 5*10^(-3); dt = Dt^2;
n = T/Dt; N = Dt/dt;

Urk = zeros(4,n);
Utilde = zeros(4,5);
Ufinal = Uzero;

for k = 1:n
    % EM method for dW21, dW2, dW12, and dW1
    Y1 = 0; Y2 = 0; Z1 = 0; Z2 = 0;
    for m = 1:N
        dW1 = sqrt(dt)*randn;
        dW2 = sqrt(dt)*randn;
        Y1 = Y1 + Y2*dW1;
        Y2 = Y2 + dW2;
        Z1 = Z1 + Z2*dW2;
        Z2 = Z2 + dW1;
    end
    % Runge-Kutta method for the system
    U = Ufinal;
    Utilde(:,1) = U + f_drift(U)*Dt;
    Utilde(:,2) = Utilde(:,1) + g1_diff(U)*0.5*(Z2^2-Dt)/sqrt(Dt)...
                              + g2_diff(U)*Y1/sqrt(Dt);
    Utilde(:,3) = Utilde(:,1) + g1_diff(U)*Z1/sqrt(Dt)...
                              + g2_diff(U)*0.5*(Y2^2-Dt)/sqrt(Dt);
    Utilde(:,4) = Utilde(:,1) - g1_diff(U)*0.5*(Z2^2-Dt)/sqrt(Dt)...
                              - g2_diff(U)*Y1/sqrt(Dt);
    Utilde(:,5) = Utilde(:,1) - g1_diff(U)*Z1/sqrt(Dt)...
                              - g2_diff(U)*0.5*(Y2^2-Dt)/sqrt(Dt);
    Ufinal = U + 0.5*(f_drift(U)+f_drift(Utilde(:,1)))*Dt...
               + Z2*g1_diff(U)...
               + 0.5*sqrt(Dt)*(g1_diff(Utilde(:,2))-g1_diff(Utilde(:,4)))...
               + Y2*g2_diff(U)...
               + 0.5*sqrt(Dt)*(g2_diff(Utilde(:,3))-g2_diff(Utilde(:,5)));
    Urk(:,k) = Ufinal;
end
%% plot the solution paths

% subplot(221)
% plot3(Urk(1,:),Urk(2,:),Urk(3,:),'r-','markersize',10), hold on
```

```
% xlabel('x'); ylabel('y'); zlabel('z1');
% view(20,30);
% axis square;
% axis tight;
% box on; ax = gca;
% ax.BoxStyle = 'full';
%
% subplot(222)
% plot3(Urk(1,:),Urk(2,:),Urk(4,:),'b–','markersize',10), hold on
% xlabel('x'); ylabel('y'); zlabel('z2');
% view(20,30);
% axis square;
% axis tight;
% box on; ax = gca;
% ax.BoxStyle = 'full';
%
% hist2d([Urk(1,:); Urk(2,:)]), hold on
% xlabel('x'); ylabel('y');
% box on; ax = gca;
% ax.BoxStyle = 'full';

%hist2d([Urk(2,:); Urk(3,:)]), hold on
%box on; ax = gca;
%ax.BoxStyle = 'full';

%hist2d([Urk(1,:); Urk(4,:)]), hold on
%box on; ax = gca;
%ax.BoxStyle = 'full';

subplot(221)
plot([0:Dt:T],[Uzero(1),Urk(1,:)],'r–','markersize',10), hold on
xlabel('relative time'); ylabel('x');
%axis square; axis tight;

subplot(222)
plot([0:Dt:T],[Uzero(2),Urk(2,:)],'b–','markersize',10), hold on
xlabel('relative time'); ylabel('y');
%axis square; axis tight;
%hist2d([Urk(1,:);Urk(2,:)])

subplot(223)
plot([0:Dt:T],[Uzero(3),Urk(3,:)],'g–','markersize',10), hold on
xlabel('relative time'); ylabel('z1');
%axis square; axis tight;

subplot(224)
plot([0:Dt:T],[Uzero(4),Urk(4,:)],'m–','markersize',10), hold on
xlabel('relative time'); ylabel('z2');
```

```
%axis square; axis tight;
end

%% Local functions f_drift
function y = f_drift(U)
r = 0.36;
l1 = 0.48;
l2 = 0.48;
e1 = 10; d1 = 0.4;
e2 = 10; d2 = 0.4;
a = 5;
y = [r*U(1)*(1 - U(1) - U(2)) - a*U(1)*U(2) - l2*U(1)*U(4)
     a*U(1)*U(2) - l1*U(2)*U(3) - U(2)
     e1*U(2)*U(3) - d1*U(3)
     e2*U(2)*U(4) - d2*U(4)];
end

%% Local function g1_diff
function y = g1_diff(U)
tau1 = 0.01;
y = [0;0;tau1*U(3);0];
end

%% Local function g2_diff
function y = g2_diff(U)
tau2 = 0.01;
y = [0;0;0;tau2*U(4)];
end
```

Listing 2: Simulating the Lyapunov exponents of the solution components

```
%% plot the Lyapunov exponents of the solution as k changes

k1 = 0.01;
k2 = 10;
n = 200;
k = linspace(k1, k2, n);
dk = (k2 - k1)/(n-1);
lambda = zeros(4,n);
z = zeros(1,n);

for i = 1:n
    lambda(:,i) = Lyapunov_exponents(k1+(i-1)*dk);
end

subplot(221)
plot(k,lambda(1,:)','r-','markersize',10), hold on
plot(k,z,'k-','markersize',10), hold on
```

```matlab
xlabel('k'); ylabel('lambda_1');
%axis square; axis tight;

subplot(222)
plot(k,lambda(2,:)','g-','markersize',10), hold on
plot(k,z,'k-','markersize',10), hold on
xlabel('k'); ylabel('lambda_2');
%axis square; axis tight;

subplot(223)
plot(k,lambda(3,:)','b-','markersize',10), hold on
plot(k,z,'k-','markersize',10), hold on
xlabel('k'); ylabel('lambda_3');
%axis square; axis tight;

subplot(224)
plot(k,lambda(4,:)','m-','markersize',10), hold on
plot(k,z,'k-','markersize',10), hold on
xlabel('k'); ylabel('lambda_4');
%axis square; axis tight;

%% Compute Lyapunov exponents of the solution
function lambda = Lyapunov_exponents(k)

rng(250)

Uzero = [0.5;0.5;k*0.01;0.01]; % Note that rho = e2/e1 = 1.
T = 2000;
Dt = 5*10^(-3); dt = Dt^2;
n = T/Dt; N = Dt/dt;

Urk = zeros(4,n);
Utilde = zeros(4,5);
Ufinal = Uzero;

for k = 1:n
    % EM method for dW21, dW2, dW12, and dW1
    Y1 = 0; Y2 = 0; Z1 = 0; Z2 = 0;
    for m = 1:N
        dW1 = sqrt(dt)*randn;
        dW2 = sqrt(dt)*randn;
        Y1 = Y1 + Y2*dW1;
        Y2 = Y2 + dW2;
        Z1 = Z1 + Z2*dW2;
        Z2 = Z2 + dW1;
    end
    % Runge-Kutta method for the system
    U = Ufinal;
```

```matlab
    Utilde(:,1) = U + f_drift(U)*Dt;
    Utilde(:,2) = Utilde(:,1) + g1_diff(U)*0.5*(Z2^2-Dt)/sqrt(Dt)...
                              + g2_diff(U)*Y1/sqrt(Dt);
    Utilde(:,3) = Utilde(:,1) + g1_diff(U)*Z1/sqrt(Dt)...
                              + g2_diff(U)*0.5*(Y2^2-Dt)/sqrt(Dt);
    Utilde(:,4) = Utilde(:,1) - g1_diff(U)*0.5*(Z2^2-Dt)/sqrt(Dt)...
                              - g2_diff(U)*Y1/sqrt(Dt);
    Utilde(:,5) = Utilde(:,1) - g1_diff(U)*Z1/sqrt(Dt)...
                              - g2_diff(U)*0.5*(Y2^2-Dt)/sqrt(Dt);
    Ufinal = U + 0.5*(f_drift(U)+f_drift(Utilde(:,1)))*Dt...
                + Z2*g1_diff(U)...
                + 0.5*sqrt(Dt)*(g1_diff(Utilde(:,2))-g1_diff(Utilde(:,4)))...
                + Y2*g2_diff(U)...
                + 0.5*sqrt(Dt)*(g2_diff(Utilde(:,3))-g2_diff(Utilde(:,5)));
    Urk(:,k) = Ufinal;
end
lambda = [log(Urk(1,n))/T
          log(Urk(2,n))/T
          log(Urk(3,n))/T
          log(Urk(4,n))/T];
end

%% Local functions f_drift
function y = f_drift(U)
r = 0.36;
l1 = 0.48;
l2 = 0.48;
e1 = 10; d1 = 0.4;
e2 = 10; d2 = 0.4;
a = 5;
y = [r*U(1)*(1 - U(1) - U(2)) - a*U(1)*U(2) - l2*U(1)*U(4)
     a*U(1)*U(2) - l1*U(2)*U(3) - U(2)
     e1*U(2)*U(3) - d1*U(3)
     e2*U(2)*U(4) - d2*U(4)];
end

%% Local function g1_diff
function y = g1_diff(U)
tau1 = 0.01;
y = [0;0;tau1*U(3);0];
end

%% Local function g2_diff
function y = g2_diff(U)
tau2 = 0.01;
y = [0;0;0;tau2*U(4)];
end
```

Listing 3: Bivariate histogram function

```
% function Hout = hist2d(D,Xn,Yn,[Xlo Xhi],[Ylo Yhi])
%
% Calculates and returns the 2 Dimensional Histogram of D.
%
% Counts number of points in the bins defined by
% X = linspace(Xlo,Xhi,Xn) and
% Y = linspace(Ylo,Yhi,Yn)
%
% D must be a 2 column or 2 row matrix or an array of complex numbers
%
% [Xlo Xhi],[Ylo Yhi] are optional and default to the min and max of
% the input data
% Xn and Yn are optional and default to 20
%
% Example:
%  hist2d([randn(1,10000); randn(1,10000)])
%
function Hout = hist2d(D,Xn,Yn,Xrange,Yrange)
% first supply optional arguments
if nargin<3
    Yn=20;
end
if nargin<2
    Xn=20;
end
if ~isreal(D)
    D=[real(D(:)) imag(D(:))];
end

if (size(D,1)<size(D,2) && size(D,1)>1)
    D=D.';
end
if size(D,2)~=2;
    error('The input data matrix must have 2 rows or 2 columns');
end
if nargin<4
    Xrange=[min(D(:,1)),max(D(:,1))];
end
if nargin<5
    Yrange=[min(D(:,2)),max(D(:,2))];
end
%
Xlo = Xrange(1) ; Xhi = Xrange(2) ;
Ylo = Yrange(1) ; Yhi = Yrange(2) ;
X = linspace(Xlo,Xhi,Xn)' ;
Y = linspace(Ylo,Yhi,Yn)' ;
```

```
Dx = D(:,1) ; Dy = D(:,2) ;
n = length(D) ;

H = zeros(Yn,Xn) ;

for i = 1:n
    x = dsearchn(X,Dx(i)) ;
    y = dsearchn(Y,Dy(i)) ;
    H(y,x) = H(y,x) + 1 ;
end ;

figure , surf(X,Y,H) ;
% Xmid = 0.5*(X(1:end-1)+X(2:end)) ;
% Ymid = 0.5*(Y(1:end-1)+Y(2:end)) ;
% figure , pcolor(Xmid,Ymid,H) ;
colorbar ;
%shading flat ;
axis square tight ;
if nargout>0
    Hout=H;
end
```

References

[1] D. J. Higham, P. E. Kloeden, *An introduction to the Numerical Simulation of Stochastic Differential Equations*, 2021, SIAM.

[2] S. Särkkä , A. Solin, *Lecture Notes on Applied Stochastic Differential Equations*, 2014.
